# Specific Evolution and Gene Family Expansion of Complement 3 and Regulatory Factor H in Fish

**DOI:** 10.3389/fimmu.2020.568631

**Published:** 2020-12-14

**Authors:** Babak Najafpour, João C. R. Cardoso, Adelino V. M. Canário, Deborah M. Power

**Affiliations:** Comparative Endocrinology and Integrative Biology, Centre of Marine Sciences, Universidade do Algarve, Faro, Portugal

**Keywords:** complement system, environment, evolution, fish, innate immunity, liver, skin

## Abstract

The complement system comprises a large family of plasma proteins that play a central role in innate and adaptive immunity. To better understand the evolution of the complement system in vertebrates and the contribution of complement to fish immunity comprehensive *in silico* and expression analysis of the gene repertoire was made. Particular attention was given to C3 and the evolutionary related proteins C4 and C5 and to one of the main regulatory factors of C3b, factor H (Cfh). Phylogenetic and gene linkage analysis confirmed the standing hypothesis that the ancestral *c3*/*c4*/*c5* gene duplicated early. The duplication of *C3* (*C3.1* and *C3.2*) and *C4* (*C4.1* and *C4.2*) was likely a consequence of the (1R and 2R) genome tetraploidization events at the origin of the vertebrates. In fish, gene number was not conserved and multiple *c3* and *cfh* sequence related genes were encountered, and phylogenetic analysis of each gene generated two main clusters. Duplication of *c3* and *cfh* genes occurred across the teleosts in a species-specific manner. In common, with other immune gene families the *c3* gene expansion in fish emerged through a process of tandem gene duplication. Gilthead sea bream (*Sparus aurata*), had nine *c3* gene transcripts highly expressed in liver although as reported in other fish, extra-hepatic expression also occurs. Differences in the sequence and protein domains of the nine deduced C3 proteins in the gilthead sea bream and the presence of specific cysteine and N-glycosylation residues within each isoform was indicative of functional diversity associated with structure. The diversity of C3 and other complement proteins as well as Cfh in teleosts suggests they may have an enhanced capacity to activate complement through direct interaction of C3 isoforms with pathogenic agents.

## Introduction

In vertebrates, the innate and adaptive immune response provides protection from pathogens. The innate immune system was the first defense mechanism to evolve and includes a range of non-specific mechanical and physiological barriers (e.g. skin, mucous, pH, lysozyme, etc), and granulocytes, macrophage and dendritic cells that are responsible for identification and elimination of pathogens (1–3). The adaptive immune response mediated by B and T lymphocytes, first emerged in vertebrates and is characterized by its specific recognition of pathogens and antigens, immunological memory and “self and non-self” recognition (4). In fish, innate immunity appears to have a dominant role in combatting pathogens (5).

The complement system is part of the innate immune system and consists of a group of plasma proteins produced by the liver that may also act as an effector and signaling mechanism for adaptive immunity (6). More than 30 components of the complement system have been identified in vertebrates and their expression is regulated by a range of stimuli including cytokines and hormones. The complement system can be activated by the antibody dependent-classical pathway, the mannose binding protein (MBP)-lectin pathway and the alternative pathway (7–9). The classical complement pathway is triggered by interaction between the antigen-antibody complex and complement 1q (C1q) and two other effector proteins, complement 4 (C4) and complement 2 (C2) (10). The MBP-lectin pathway is triggered by carbohydrates such as mannans or N-acetylglucosamine (GlcNAc) (11), which are found in the bacterial cell wall (12). The alternative pathway (AP) does not require specific molecular recognition and is activated by hydrolysis of complement 3 (C3) by factor B, factor D and properdin and this “C3 tick-over” mechanism forms the basis of the rapid activation of all the complement pathways (13, 14). Other complement components (C5, C6, C7, C8, and C9) form the membrane-attack complex (MAC) that causes cell lysis.

All three complement pathways converge at C3, which has a central role in complement system function. C3 when enzymatically cleaved generates two protein subunits C3a and C3b, with the latter attaching to the pathogen surface and activating the lytic pathway. C3b is required for the sequential junction of C5b, C6, C7, C8, and C9 proteins to form the membrane-attack complex (MAC), which provokes cell lysis (15). C3, C4, and C5 share sequence similarity and they belong to the α2-macroglobulin (α2M) family (16). The proteins possess a variable central region associated with pathogen recognition and a thioester motif for attachment of complement to the target cells (17). Several plasma and membrane-bound proteins or complement control proteins (CCP) regulate the activity of the complement system. In mammals, complement factor H (CFH) is an important complement system regulator that binds C3b and inhibits the alternative pathway (18, 19). In humans, five CFH-related plasma proteins exist (CFHR1 to 5) (20) and CFH and CFH-related proteins have also been described in a few teleost species (21, 22).

From teleost fish to mammals, the complement pathway appears to have been conserved, although in teleosts it functions at a lower temperatures and the titre of complement components of the alternative pathway in plasma is higher (23, 24). The complement pathway has been characterized in relatively few fish species (trout, *Oncorhynchus mykiss*; sea bream, *Sparus aurata*; carp, *Cyprinus carpio*; medaka, *Oryzias latipes*; two Antarctic teleosts, *Trematomus bernacchii* and *Chionodraco hamatus*; and zebrafish, *Danio rerio*), and studies mainly focused on the multiple copies of the *c3* gene (25–32) and in zebrafish they were shown to have a different transcriptional response to LPS injection (30). There is even less information about complement regulatory factors and so far they have only been characterized in rainbow trout, yellow croaker (*Larimichthys crocea*) and zebrafish and in common with humans are present in multiple gene copies (21, 22, 33). Studies of immune challenged zebrafish, rainbow trout, yellow croaker, winter flounder (*Pseudopleuronectes americanus*) and Japanese flounder (*Paralichthys olivaceus*) indicate *cfh* responds (21, 22, 33–35).

It seems likely that the evolution of the immune system in fish was shaped by their contact with the greater number and diversity of microbes and viruses found in water compared to other vertebrates (36). The barrier function of epithelia such as the skin in vertebrates has a major role in protection and impedes the entry of pathogens. The complement system rapidly responds to pathogens that breach the damaged barrier or adsorb to and colonize the surface (37). To lay the foundation for a better understanding of the physiology of the complement system in teleosts the evolution of complement proteins and Cfh was studied in deuterostomes paying particular attention to the teleosts. The wealth of available fish genomes was exploited to pinpoint specific events in complement evolution by including in the analysis a diversity of phylogenetically informative vertebrate taxa. Considering the proposed importance of the alternative pathway in teleosts (38) and the key role of C3 in the lytic pathway the evolution of C3 isoforms was targeted. The results of the global analysis of the genes of the complement system in vertebrates indicate it was well conserved during evolution. Of note was the highly variable number of *c3* genes in different fish and so this and the related *c4* and *c5* genes were examined in more detail. By conducting a multi-species analysis of *c3*/*c4*/*c5* using evolutionary informative species, with particular emphasis on the most successful group of vertebrates, the teleosts, (39) a consensus evolutionary model was proposed. The model reveals that duplication of *c3* is common across the fishes and that the complement system in fish has been under different evolutionary pressure compared to other vertebrates. Our data confirm that the *c3* gene expansion previously identified (25–32) occurred across the teleosts and that the deduced proteins form two main clusters, C3.1 and C3.2. In common, with other immune gene families the *c3* gene expansion in fish emerged through a process of tandem gene duplication, a process associated with an unstable genomic organization and the appearance of new genes (40, 41). Furthermore, retention of multiple *c3* gene copies was not associated with specialization through tissue specific expression. However, analysis of the differences in the sequence and domains of the nine deduced C3 proteins in the gilthead sea bream (*Sparus aurata*), and the presence of specific cysteine and N-glycosylation residues within each isoform was indicative of potential functional diversity associated with structure. In vertebrates in which *c3* gene expansion occurred, *cfh-like* the C3b regulatory factor also underwent expansion so that a species-specific gene repertoire emerged. Overall, the results indicate that in fish complement signaling and regulatory proteins shared common ancestry with the tetrapod homologues but evolved under distinct pressures.

## Material and Methods

### Screening for the Complement System and Complement Regulatory Factor H in Ray-Finned Fish

In order to analyze and understand the evolution of the complement system (C1-C9) in fish and its regulation, orthologues of human C1QA, C1QB, C1QC, C2, C3, C4 (C4A/C4B), C5, C6, C7, C8 (α/β/δ), and C9 genes and complement regulatory factor (CFH and CFHR) genes were procured in the genomes of several ray-finned fish using tBLASTn (**Supplementary Table 1**) (42). The species analyzed included, the spotted gar (*Lepisosteus oculatus*), a basal ray-finned fish that radiated prior to the teleost expansion, and 12 teleost genomes: the Amazon molly (*Poecilia formosa*), cave fish (*Astyanax mexicanus*), cod (*Gadus morhua*), Fugu (*Takifugu rubripes*), medaka (*Oryzias latipes*), platyfish *(Xiphophorus maculatus)*, stickleback (*Gasterosteus aculeatus*), Tetraodon, (*Tetraodon nigroviridis*), tilapia (*Oreochromis niloticus*), European sea bass (*Dicentrarchus labrax*), Gilthead sea bream (*Sparus aurata)* and zebrafish (*Danio rerio*) (**Supplementary Table 1**). Sequences were identified based on their high sequence similarity (*e-value* ≤ 1e ^-40^) with human homologues and *in silico* genome annotations. The zebrafish complement component and CFH/CFHL sequences retrieved were subsequently used to search against ray-finned fish genomes to retrieve missing/non-annotated hits. Sequence identity was confirmed by searching the NCBI non-redundant protein sequence database using the human filter (taxid:9606) and the isolated fish genes and also by phylogeny (see methods below). The results from the general analysis of the complement system in fish revealed the *c3* genes and the *c4* genes, which belong to the alpha-2 macroglobulin superfamily of thioester containing proteins, underwent a large expansion. For this reason, the members of the alpha-2 macroglobulin superfamily of thioester containing proteins, C3, C4, and C5 were selected for more in-depth analysis. The fish *cfh* and putative *cfhr* family genes, that encode complement regulator proteins, were also further analyzed as multiple genes were found. All searches were performed against the most recent annotated fish genome assemblies available from ENSEMBL or NCBI (**Supplementary Table 1**).

### In Depth Analysis of c3, c4 and c5 and cfh Genes

The deduced proteins of human C3, C4, C5, CFH, and CFHRs were used as the bait for database searches which included the fish listed above and other teleost fish genomes; the flatfish, smooth tongue sole (*Cynoglossus semilaevis*) and Japanese flounder (*Paralichthys olivaceus*), Antarctic black rockcod (*Notothenia coriiceps*), and Atlantic salmon (*Salmo salar*). We also interrogated genomes of fish species that diverged earlier in the vertebrate radiation and thus are considered to possess less rearranged genomes: the lobe-finned coelacanth (*Latimeria chalumnae*), that diverged basal to the tetrapods; two cartilaginous fishes, the elephant shark (*Callorhinchus milii*) and whale shark (*Rhincodon typus*), that are basal to the bony vertebrates and two Agnathans (jawless fish), the sea lamprey (*Petromyzon marinus*) and the inshore hagfish (*Eptatretus burgeri*) which diverged prior to the gnathostomes (**Supplementary Table 1**). For comparative analysis the mouse (*Mus musculus*), the chicken (*Gallus gallus*), the reptile (*Anolis carolinensis*), and the amphibian clawed African toadfish (*Xenopus tropicalis*) were also included in the analysis as well as two basal deuterostomes, the urochordate Ciona (*Ciona intestinalis*) and the cephalochordate Amphioxus (*Branchiostoma floridae*) genomes to infer the likely evolutionary origin of the vertebrate complement proteins and CFH-family members (**Supplementary Table 1**).

### Sequence Comparisons and Phylogenetic Analysis

Multiple sequence alignments were performed using the MUSCLE algorithm (43) in the Aliview platform (44) and conserved regions were identified. Searches in ray-finned fish genomes and transcriptomes identified many incomplete genes/transcripts that encoded for complement protein fragments. When multiple transcripts (of variable sizes) for the same gene were found they were aligned and sequences merged (> 98% sequence identity) to obtain a full-length protein sequence. GeneDoc (http://www.nrbsc.org/gfx/genedoc) was used to calculate identity/similarity between the sequences. Only the deduced protein sequences that encoded full-length C3 proteins were considered for phylogenetic analysis (**Supplementary Table 1**).

Phylogenetic trees were constructed based on alignments of the deduced amino acid (aa) sequence of the fish and tetrapod proteins including the invertebrate deuterostome genes (urochordate and cephalochordate). Sequences were aligned using the MUSCLE algorithm in the AliView platform and the alignment was edited to remove sequence gaps and poorly aligned regions. Phylogenetic trees were constructed using Maximum-Likelihood (ML) and Bayesian Inference (BI) methods and the models that best fit the data were calculated in model test-ng 0.1.5. ML and BI trees were constructed in the CIPRES Science Gateway V.3.3 (45) and run on XSEDE. ML trees were built with the RAxML v8.2.12 (46) method with a WAG matrix and 1000 bootstrap replicates and BI trees using MrBayes (47) with a WAG matrix and 1.000.000 generation sampling and probability values to support tree branching. For the fish Cfh tree a similar strategy to that for complement proteins (outlined above) was used, other Cfh related proteins, CFHR from human and Cfhl from zebrafish (21) were also included in the alignment; the sequence file was manually edited to delete gaps and misaligned sequences before tree building using the BI method with a VT matrix. All trees were displayed in FigTree (http://tree.bio.ed.ac.uk/software/figtree/). The C3/C4/C5 trees were rooted with the deduced protein sequences of human α-macroglobulin (A2M, ENST00000318602.12) and cluster of Differentiation 109 (CD109, ENST00000437994.6). The CFH/CFHR tree was rooted with the predicted protein sequence of human coagulation factor XIII B (ENST00000367412.1). Trees for the fish C1, C2/Cfb, C6, C7, C8, and C9 were also built to confirm sequence identity and were constructed using the BI method and default settings and they were rooted using the human orthologue.

### Neighboring Gene Analysis 

To better characterize the evolution of the multiple gene copies of *c3* and *c4*, the neighboring gene environment of these genes in teleosts along with *c5* and *cfh* genes was characterized. Homologues of human *C3*, *C4*, and *C5* neighboring genes were procured in the coelacanth, spotted gar, elephant shark and four teleost genomes (tetraodon, stickleback, medaka and zebrafish). The species selection was based on the quality of the available genomes and distinctive evolutionary patterns suggested by the number of genes identified. For comparison, the neighboring gene environment of the human, lizard or chicken were also characterized. Fifteen genes upstream and downstream of human *C3*, *C4*, and *C5* gene loci were retrieved and homologues were identified in the other species analyzed using the genome annotations provided by Genomicus software (http://www.genomicus.biologie.ens.fr) and by homology sequence searches in the genomes available from ENSEMBL. The gene environment of the *C4* gene in the chicken was characterized in-depth as phylogenetic analysis suggested that an extra gene copy exists.

### Protein Motif Annotation

To identify changes indicative of potential functional divergence between the vertebrate orthologues, the fish paralogues and the species-specific duplicates, the deduced protein sequence of selected fish C3, C4, and C5 and the multiple C3 isoforms from the gilthead sea bream were annotated and compared with the homologues in human and chicken. Sequence alignments were performed using the MUSCLE algorithm in the Aliview platform and edited in GeneDoc software (http://www.nrbsc.org/gfx/genedoc). The signal peptide was deduced using SignalP 4.1 Server (http://www.cbs.dtu.dk/services /SignalP/) identification of protein domains was carried out based on the crystallographic structure of the human C3 (48) and homology modelling of an Antarctic teleost C3 (32). Sequence alignments of the thioester-bound domain of fish C3 and C4 deduced proteins and the catalytic site for C4 were analyzed in detail. The amino acid residues associated with protein structure, conformation and signaling such as the cysteine residues responsible for disulphide bridges and N-glycosylation sites (N-X-T/S) were mapped in human C3 using Uniprot annotation (https://www.uniprot.org) and identified manually in the gilthead sea bream.

### Transcriptome Analysis

To increase the number of teleost complement sequences available for analysis and to infer the importance and functional divergence of the complement components in teleost skin, expression data was analyzed. Databases of gene transcripts of the European sea bass and gilthead sea bream (*Sparus aurata*) (49) and RNA-seq transcriptome (Illumina platform) assemblies of the European sea bass skin (GFJW00000000) (50), Senegalese sole skin (PRJEB29449) (51) and the *de novo* transcriptome assemblies of the intestine, skin and head-kidney of two Antarctic fish, the black rockcod and marbled rockcod (*Notothenia rossii*, **Supplementary Table 1**), were interrogated. The nucleotide sequences (*e-value* ≤ 1*e* ^-30^) were retrieved and translated into proteins using the Expasy translation tool (https://web.expasy.org/translate/) and annotation was assigned using blastp against the human (taxid:9606) and confirmed by phylogenetic analysis. Skin transcriptome data was complemented with data retrieved from previously published transcriptome and proteome studies of fish skin and mucous.

### RNA Extraction and cDNA Synthesis

To further confirm *c3* gene expression and extend understanding of the persistence in the genome of the multiple gene isoforms, total RNA (tRNA) from the gills, spleen and liver (n= 3) of gilthead sea bream (average weight = 87.09 ± 5.54 g) that was already available in the laboratory and stored at -80°C in the context of a previous study of skin regeneration was used (52). Reactions for cDNA synthesis contained 500 ng of the DNase treated tRNA (denatured at 65°C for 5 min), 10 ng of pd(N)6 random hexamers (Jena Bioscience, Germany), 2 mM dNTPs (ThermoScientific, USA), 100 U of RevertAid Reverse Transcriptase and 8 U Ribolock RNAse inhibitor (ThermoScientific) in a final reaction volume of 20 μl. The time and temperature cycle of the cDNA synthesis reaction was 10 min at 20°C; 50 min at 42°C and 5 min at 70°C. The integrity and quality of the synthetized cDNA was assessed by amplification of the sea bream ribosomal subunit *18s* rRNA using the following thermocycle: 95°C for 10 min followed by 25 cycles of (95°C for 20 s; 60°C for 20 s; 72°C for 20 s) and a final cycle at 72°C for 5 min.

### Quantitative Expression of Sea Bream c3 Isoforms 

Specific primers for each gilthead sea bream *c3* gene isoform were designed and the amplified PCR products were sequenced to confirm their identity. Five of the nine identified gilthead sea bream *c3* gene transcripts (*c3.1.1, c3.1.2, c3.1.3, c3.1.4,* and *c3.2*) were successfully amplified. Difficulties with design of specific and efficient qPCR primers meant that *c3.1.5*, *c3.1.6*, *c3.1.7*, and *c3.1.8* were not quantified. The tissue distribution of the *c3* gene transcripts was analyzed in the gill, spleen and liver (**Table 1**). Quantitative real-time PCR (qPCR) reactions were performed in duplicate (< 5% variation between replicates) using a BioRad CFX Connect Real Time System and SsoFast EvaGreen supermix (Bio-Rad, Portugal) and 96-well plates (Axygen). The gilthead sea bream 18S ribosomal subunit (*18s*) was used as the reference gene as it showed stable expression levels in all samples. The final qPCR reaction volume was 10 µl and contained 200 nM of each primer, 2 µl of the template cDNA (diluted 1:5 target gene, 1:5000 for *18s*) and 8 µl of EvaGreen Supermix (Bio-Rad, Portugal). Thermocycling conditions were 95°C for 30 s and was followed by 39 cycles of (95°C for 5 s and 60°C for 10 s, **Table 1**). To detect non-specific amplification products and primer dimers melting curves were performed. Standard curves were included in PCR plates for each *c3* gene isoform and prepared from serial dilutions of the sequenced and quantified amplicons. Control reactions were included in all runs to confirm the absence of qPCR or genomic contamination. qPCR reaction efficiencies and r2 (coefficient of determination) were all > 90% for each target gene transcript. Expression normalization was performed using *18s* ribosomal RNA (**Table 1**).

**Table 1:** List of primers used to amplify *c3* and *18s* ribosomal RNA genes in gilthead sea bream.

Name	Sequence (5’-3’)	Temp (°C)	Efficiency (%)	R2
Complement gene				
*c3.1.1Fwd*	GATCAGGTTGGAGAACCCAG	60	101.4	0.99
*c3.1.1Rev*	GACCCTGTCTCCTTCAGAAC			
*c3.1.2Fwd*	GTTAAGGTCACTGAGTGATGC	60	92.7	0.99
*c3.1.2Rev*	GCTCACTAAAGTGCCTTTACTC			
*c3.1.3Fwd*	GCTTTGAAACATAAGAACTGCACA	60	91.6	0.99
*c3.1.3Rev*	TCCATTTGCCAGAGGTTAATTTGT			
*c3.1.4Fwd*	GATGAACAGAGTCAGGCGTAC	62	94.3	1
*c3.1.4Rev*	CATCCGTGTTGCGTGTACTTC			
*c3.2Fwd*	GACCTGAGGGACACAGTCAG	60	97.6	0.99
*c3.2Rev*	CACGGTGGACTTGCTGAAGT			
Reference gene				
*18s Fwd*	TGACGGAAGGGCACCACCAG	60	90.3	1
*18s Rev*	AATCGCTCCACCAACTAAGAACGG			

The annealing temperature, primer pairs efficiency (%) and the linearity R^2^ of the standard curve are indicated.

## Results

### Complement System and Regulatory Proteins in Ray-Finned Fish 

**Figure 1:**
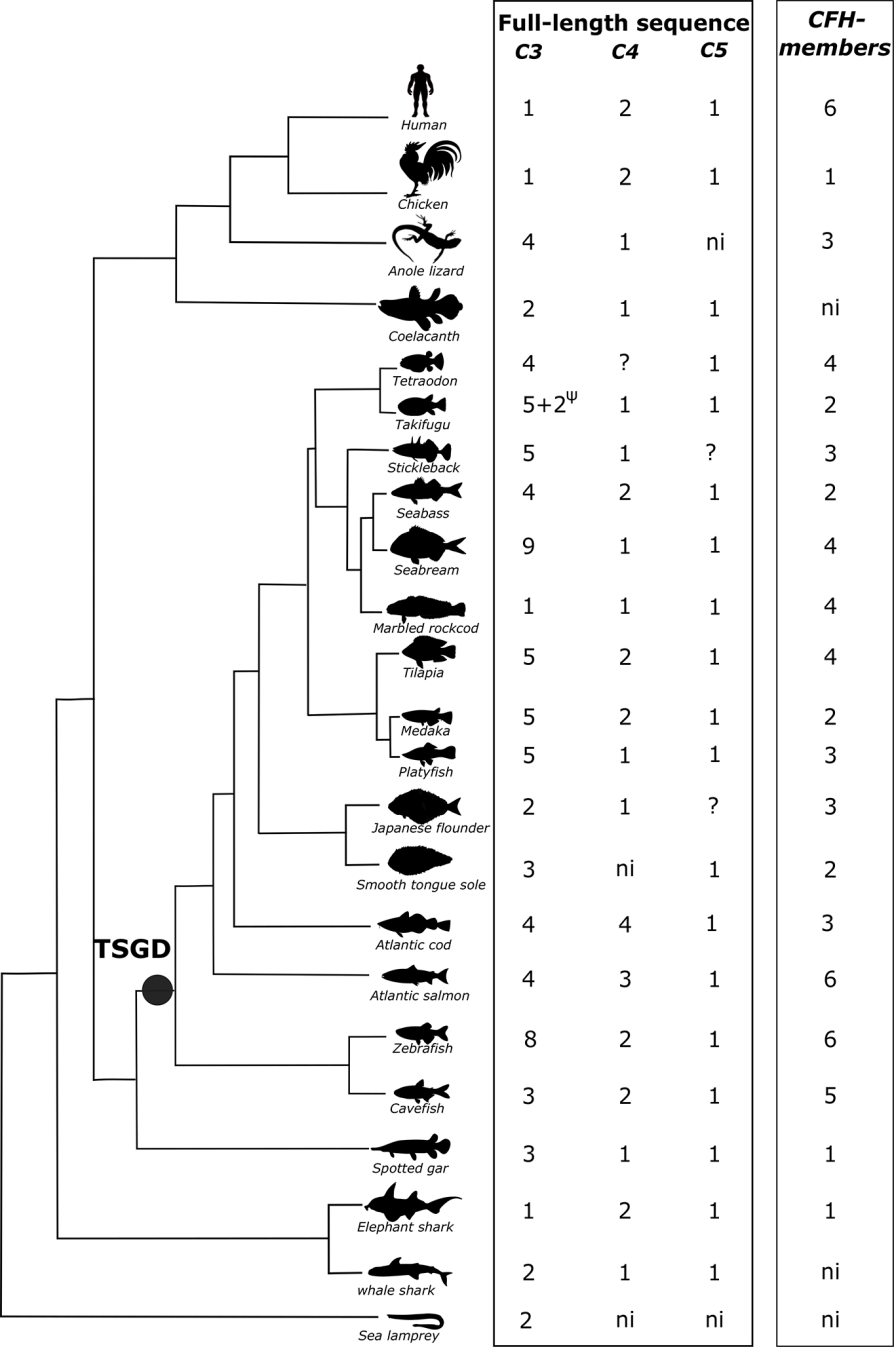
Dendrogram showing the number of predicted C3, C4, C5, and CFH genes and transcripts identified in fish and other vertebrates. The number of full-length/complete C3, C4, and C5 sequences are indicated. Incomplete C3 sequences were also found for stickleback, sea bass, marbled rockcod, medaka, smooth tongue sole and Atlantic salmon (**Supplementary Table 1**). C4 incomplete sequences were found in the whale shark (**Supplementary Table 1**). ?, only incomplete sequences found. ψ, pseudogene; ni, not identified; TSGD, teleost specific genome duplication.

Initial screening for members of the complement cascade revealed that in ray-finned fish some genes of the signaling pathway have duplicated but a larger expansion of the complement 3 (*c3*) gene occurred when compared to human (**Table 2**). In fish, three *c3* genes were retrieved from the spotted gar genome but the number of *c3* genes varied across the analyzed teleost genomes (**Figure 1**). A large expansion of *c3* genes was found in the sea bass genome and many hits corresponded to small incomplete genes. Duplicates of complement genes encoding *c4* and *c6* were identified in some fish genomes and in cod four *c4* genes were found and in all the teleost genomes analyzed duplicate *c7* genes (*c7a*, *c7b*) persisted (**Figure 1**, **Table 2**, **Supplementary Figure 1**). For other complement members the gene number was similar between ray-finned fish and human with some exceptions such as the identification of four *c2/cfb* in zebrafish (*c2/cfb.1.1*, *c2/cfb.1.2*, *c2/cfb.1.3*, *c2/cfb.2*) and two *c9* (*c9.1*, *c9.2*) in the cod genome (**Table 2**, **Supplementary Figure 1**). The *cfh* gene family, in common with the *c3* genes, underwent a large expansion in ray-finned fishes and gene number ranged from a single gene copy in the spotted gar, two copies in the sea bass to five copies in cavefish and six in the zebrafish and Atlantic salmon. This suggests that in addition to the expansion of the *c3* gene, expansion of *cfh* its regulatory factor also occurred in teleosts (**Table 2**).

**Table 2:** Number of genes of the complement system found in ray-finned fishes.

	*C1Q(A, B, C)*	*C2,C2/CFB*	*C4*	*C3*	*C5*	*C6*	*C7*	*C8(α, β, δ)*	*C9*	*CFH members*
Human	3	2	2	1	1	1	1	3	1	1
Spotted gar	3	2	1	3	1	1	1	3	1	1
Amazon molly	2 (A, C)	2	2	7	1	2	2	3	1	1
Cavefish	3	3	2	3	1	1	2	3	1	5
Cod	3	2	4	4	1	1	2	2 (α, β)	2	3
Fugu	3	2	1	5	1	1	2	3	1	2
Medaka	3	2	2	5 (1)	1	1	2	3	1	2
Platyfish	3	2	1	5	1	1	2	3	1	3
Stickleback	1 (C)	2	1	5 (3)	1	1	2	3	1	3
Tetraodon	3	2	2	4	1	1	2	3	1	4
Tilapia	3	2	2	5	1	1	2	3	1	4
Zebrafish	3	4	2	8	1	2	2	3	1	6
Sea bass	3	2	2	4 (10)	1	1	2	3	1	2
Sea bream	3	2	1	9	1	1	2	3	1	4

The identified human gene copies are indicated for comparison. The number of incomplete C3 genes found in teleost genomes is indicated within brackets. Searches included the genes of the three chains of C1Q (A, B, C) and of C8 (α, β, δ). Phylogenetic trees for the fish C1q, C2/Cfb, C6, C7, C8, and C9 are available in **Supplementary Figure 1**.

### The c3, c4, c5, and cfh Family Genes in Fish

#### Agnathan and Cartilaginous Fishes

In the sea lamprey, two *c3* genes (*c3.x.1* and *c3.x.2*) were found and they mapped in tandem in chromosome 14 but in the inshore hagfish genome assembly four putative *c3* genes were identified (**Supplementary Table 1**). The two predicted lamprey C3 proteins are likely to be complete but in hagfish the predicted proteins from the four genes are incomplete and one gene (*c3.y1*) encoded the transcript that was previously reported (53). In hagfish two of the duplicate c3 genes (*c3.y*1 and *c3.y2*) also mapped in close proximity in the same genome region (FYBX02010427.1) suggesting a similar genome structure to that found in the sea lamprey.

For cartilaginous fish, in the elephant shark a single *c3* gene was retrieved but for the whale shark two genes were found (**Figure 1**, **Supplementary Table 1C** **3**). In lamprey and hagfish, no gene homologues of human *C4* or *C5* were identified. In the elephant shark two *c4* genes were found but in the whale shark three hits for putative *c4* genes were obtained but only one (*c4.1.1*) coded for a full-length *c4* protein (**Figure 1**). The remaining sequences (*c4.1.2* and *c4.1.3*) were shorter and they aligned to different regions of the full-length C4 protein. It was not possible to establish if they correspond to different genes or are fragments of the same gene (**Supplementary Table 1 C**4). For *c5* in cartilaginous fish only a single gene hit was obtained and the deduced protein was full-length (**Supplementary Table 1 C**5).

A homologue of the human *CFH* gene was retrieved from the elephant shark but not from the lamprey or whale shark genome assemblies.

#### Ray-Finned Fish 

Searches in ray-finned fish genomes identified multiple putative c3 genes. In spotted gar, three c3 genes and single c4 and c5 gene were retrieved, which were of a similar length to the human orthologues (**Figure 1**). In teleosts, the number of *c3* genes varied from one in the marbled rockcod to nine in the gilthead sea bream. Sequence comparisons revealed that several of the deduced teleost *c3* genes coded for incomplete C3 proteins and that most of them lacked the protein beta-chain domain. However, searches in the NCBI transcript database identified full-transcripts for some of the *c3* genes that were incomplete in the genome assembly of the Amazon molly (*c3.1.3*, *c3.1.4* and *c3.1.5*), cavefish (*c3.1.2*), medaka (*c3.1.4*), zebrafish (*c3.1.5*), and smooth tongue sole (*c3.2*) (**Supplementary Table 1**). In the Fugu genome *c3.1.5* and *c3.1.6* genes are annotated as pseudogenes, if the incomplete genes found in other teleosts are also pseudogenes or the result of poor sequence assembly remains to be established (**Supplementary Table 1**). In the sea bass genome only two full-length *c3* genes were found (*c3.1.1* and *c3.2*) and it is in this species that the largest number of incomplete *c3* genes were retrieved. Searches in the sea bass transcriptome and NCBI database retrieved three full-length transcripts (**Supplementary Table 1 C**3) of which one is the transcript for one of the predicted full-length genes. The other two transcripts did not align (> 98% nucleotide identity) with any genome region suggesting there may be errors in the genome or transcriptome assemblies (**Supplementary Table 1 C**3).

For *c4* and *c5* a single gene was retrieved in most species except for tilapia, medaka, sea bass, cavefish and zebrafish where two *c4* genes were identified and the salmon and cod that had three and four genes, respectively (**Figure 1**). In tetraodon, two putative genes for *c4* (one that maps to chromosome 8 and another that maps to a non-annotated chromosome) were retrieved but sequence alignment of the translated proteins with the full-length C4 from human/fish revealed that they do not overlap and aligned to different regions. If they correspond to two independent genes or are fragments of the same gene remains to be established. In the Japanese flounder genome three hits for a putative *c5* gene were retrieved that most likely represent fragments of a single gene since although they did not overlap, they aligned with different regions of the human/fish full-length homologue proteins.

In the ray-finned fish a single gene for a putative *cfh* family member was found in the spotted gar but in teleosts the gene number varied from two in fugu, medaka, sea bass and smooth tongue sole to five and six in cavefish and zebrafish genomes, respectively (**Figure 1**).

#### Lobe-Finned Fish and Tetrapods

Two *c3* and a single *c4* and *c5* gene were found in the coelacanth but our sequence searches failed to retrieve a putative *cfh* gene from the lobe-finned fish genome. In tetrapods multiple *C3* genes and *Cfh* genes exist. In the amphibian, *Xenopus* five full-length *C3* genes and a single *Cfh* gene were found but for the reptile, the anole lizard four *C3* gene copies were retrieved and three gene copies of *Cfh* also exist (**Supplementary Table 1**, **Figure 1**). In the chicken gene number was similar to human and single *C3* and *C5* genes and duplicate *C4* genes were found but only a single *Cfh* family member was retrieved (**Figure 1**, **Supplementary Table 1**).

### Phylogenetic Analysis

#### C3, C4, and C5 Phylogeny

**Figure 2:**
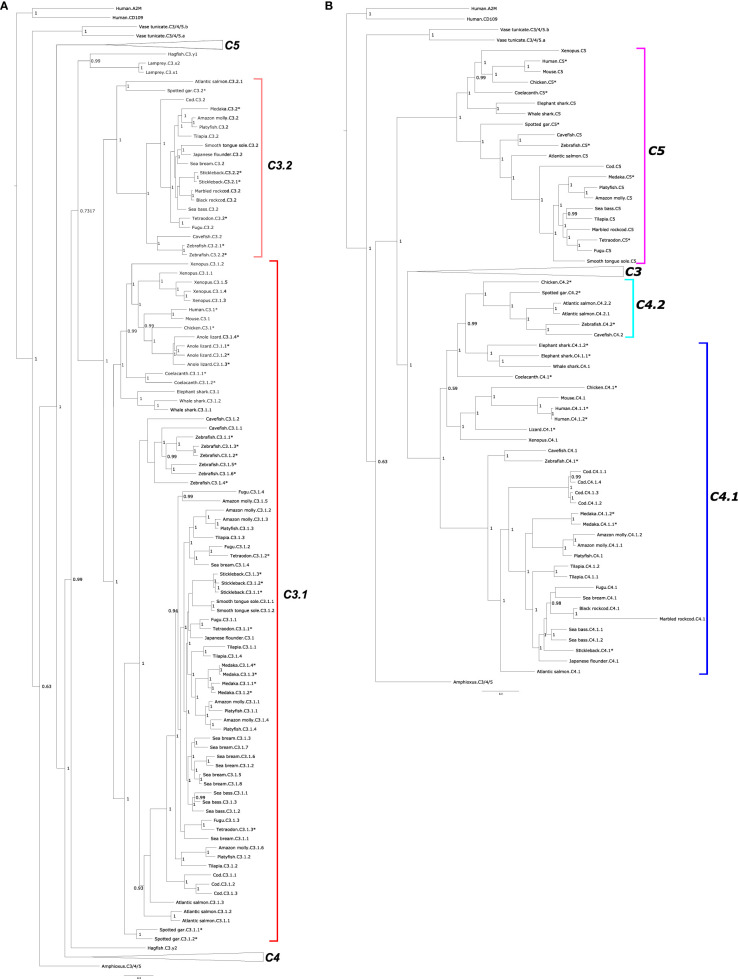
Phylogenetic tree of complement factors C3, C4, and C5 in fish and other vertebrates. The tree was built only with full-length (both β- and α-chain) sequences and homologues from a urochordate (*Ciona intestinalis*) and a cephalochordate (*Branchiostoma floridae*) were also included to understand the evolutionary context. The tree was built with the BI method and branch support values (posterior probability values) are shown. Two subsets of the same phylogenetic tree showing different family members: **(A)** C3 and **(B)** C4 and C5 are represented to facilitate interpretation. In **(A)** the two major C3 clades were named C3.1 (red) and C3.2 (pink). For **(B)** the two C4 clades were named C4.1 (dark blue) and C4.2 (light blue). The teleost C5 clade is boxed in purple. The C4 sequences of the coelacanth and cartilaginous fishes (elephant shark and whale shark) were named C4.1 based on the similarity of their gene environment with the other vertebrate C4.1 genome regions. Duplicated fish *c3* and *c4* genes are numbered arbitrarily. The tree was rooted with the deduced protein sequences from human α-macroglobulin (A2M, ENST00000318602.12) and cluster of Differentiation 109 (CD109, ENST00000437994.6). The species for which gene linkage analysis was performed (**Figures 4**–**6**) are indicated by an asterisk (*). Sequence accession numbers are available in **Supplementary Table 1** and nototheniid deduced protein sequences are available as **Supplementary Data**. A similar tree built with the Maximum Likelihood (ML) method is presented as **Supplementary Figure 2**.

Phylogenetic trees of C3, C4, and C5 suggest that they emerged prior to the vertebrate radiation from a common ancestral gene via gene duplications (**Figures 2A, B** and **Supplementary Figure 2**). According to the tree topology, C5 was the first member to diverge and vertebrate C3 and C4 emerged subsequently. The C3 clade possess the largest number of members (**Figure 1**). The C3 clade was rooted with the lamprey and hagfish C3 proteins and two main sequence clusters were found: one cluster contained C3 from tetrapods, coelacanth and sharks and included C3 from most of the teleosts and was designated C3.1 and the second cluster contained only ray-finned fish sequences and was named C3.2 (**Figure 2A** and **Supplementary Figure 2**). The two spotted gar C3 (C3.1.1 and C3.1.2) sequences clustered within the C3.1 clade and the third clustered within the C3.2 clade indicating that the C3 gene duplication that originated *c.3.1* and *c.3.2* genes occurred prior to the ray-finned fish radiation. The clustering of the multiple ray-finned fish sequences within the C3.1 and C3.2 clusters suggests that they arose through lineage or species-specific duplication events. The tree topology also suggests that the multiple *c3* gene copies found in lizard and *Xenopus* and the tunicate *c3/4/5* sequences also arose through species-specific duplication events.

For C4, the tree topology indicated that two distinct protein clusters existed, and they were designated C4.1 and C.4.2 (**Figure 2B**, **Supplementary Figure 2**). The C4.1 cluster contained sequences from tetrapods and most of the teleost C4 sequences including 4 cod members, C4.1.1, C4.1.2, C4.1.3, C4.1.4, which based on clustering probably arose from a species-specific duplication. The C4.2 cluster contained a C4 sequence from the chicken along with the spotted gar C4 and the deduced protein of one gene from zebrafish and cavefish and two genes from the Atlantic salmon (C4.2.1 and C4.2.2). The lobe-finned fish C4 and the two cartilaginous fish C4s tend to group within the C4.2 cluster but analysis of their neighboring gene environment suggests that they are *c4.1* genes (see *Short-Range Gene Linkage* section). Clustering of the fish C4 sequences suggested that in common with the fish *c3* genes they expanded by lineage and species-specific duplication events. The existence of two *C4* genes in the chicken, which clustered independently in sister clades along with the teleost paralogues suggests that the C4.1 and C4.2 genes resulted from a gene duplication that occurred early in the vertebrate radiation and that the duplicate gene copies only persisted in the genomes of a few ray-finned fish species and in the chicken (**Figure 2B**).

Within the C5 cluster two main branches exist one containing C5 from ray-finned fishes and the other cluster containing C5 from the cartilaginous fish, the coelacanth and the tetrapods (**Figure 2B**, **Supplementary Figure 2**).

#### Cfh Family Member Phylogeny

**Figure 3:**
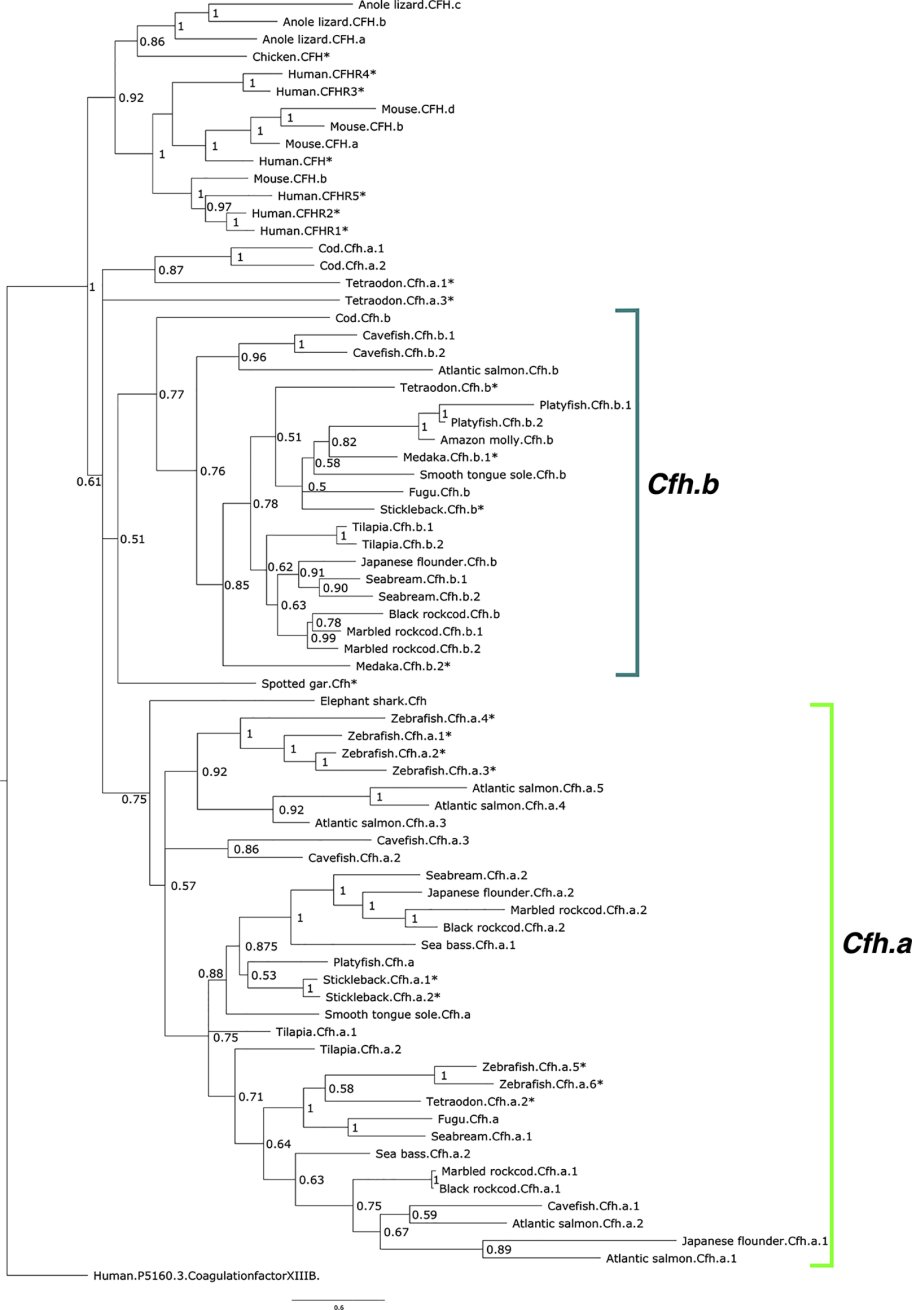
Phylogenetic tree of the fish and other vertebrate complement factor H (CFH). The tree was built with the BI method and branch support values (posterior probability values) are shown. The two major CFH clades in teleosts were named Cfha (light green) and Cfhb (green-blue) and arose from the teleost genome duplication event and the multiple genes found in each species were numbered arbitrarily. The tree was rooted with the predicted protein sequence of human coagulation factor XIII B (ENST00000367412.1). The species for which gene linkage analysis was performed are indicated with an asterisk (*). Accession numbers of the sequences that were used to build the tree are available in **Supplementary Table 1**. The sequence of the *Xenopus* Cfh was not included in the tree to simplify the analysis as clustering indicated its evolution was highly divergent from all other species.

The phylogenetic tree of the deduced proteins of fish Cfh-members suggested that they shared common ancestry with human CFH/CFHR. Fish Cfh-members grouped in two main teleost sequence clusters named, Cfha and Cfhb, that probably arose from the teleost genome duplication event (**Figure 3**). The distribution of the deduced fish Cfh proteins in the tree indicated that evolution of this protein family was complex and the arrangement of the sequences within each cluster suggested they evolved in a lineage and species-specific manner (**Figure 3**). All zebrafish sequences grouped in the Cfha cluster and some sequences clustered in close proximity suggesting they represent recent species-specific duplications. The deduced protein of the two medaka *cfh* genes clustered in the Cfhb clade but the stickleback, cavefish and Tetraodon had *cfh* genes in both clades.

### Short-Range Gene Linkage

**Figure 4:**
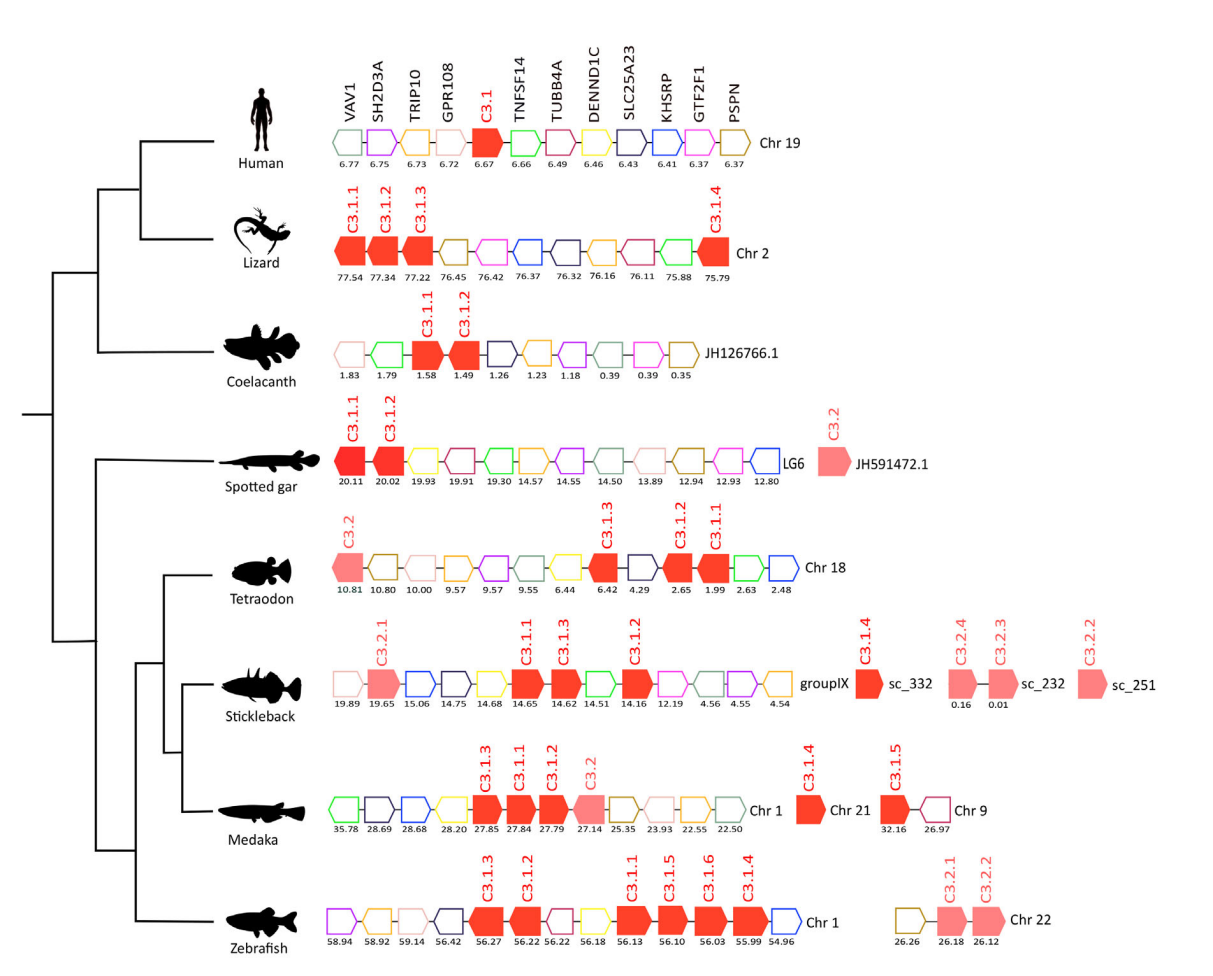
Gene synteny analysis of the *C3* gene environment in tetrapods and fish. Genes predicted in the genome are represented by arrows and the arrowheads indicate the gene orientation. *C3* genes are represented by full-colored arrows: C.3.1 in red and C.3.2 in pink. Neighboring gene families are represented by different colors and gene homologues are indicated in the same color. The genes are mapped on the chromosomes and their actual positions (Mega base pairs, Mbp) is indicated below. Only genes that were conserved in the homologous genome regions in all species compared are represented. Tumor necrosis factor 14 (*TNFSF14*), Tubulin beta-4A chain (*TUBB4A*), DENN domain-containing protein 1C (*DENND1C*), Solute carrier family 25 member 23 (*SLCA25A*), Far upstream element-binding protein 2 (*KHSRP*), General transcription factor IIF subunit 1 (*GTF2F1*), Persephin (*PSPN*), Protein GPR108 (*GPR108*), Cdc42-interacting protein 4 (*TRIP10*), SH2 domain-containing protein 3A (*SH2D3A*), Proto-oncogene-vav (*VAV1*).

**Figure 5:**
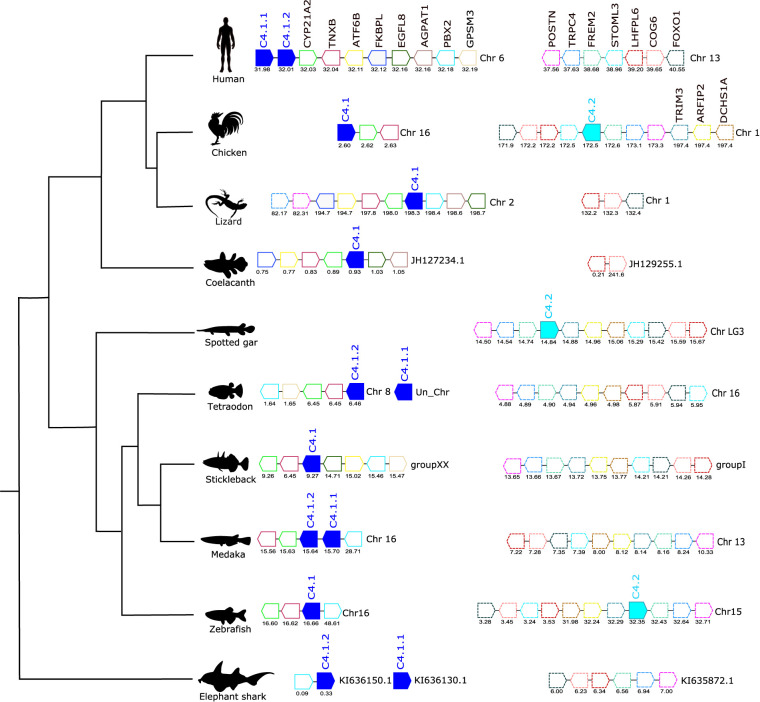
Gene synteny analysis of the *C4* gene environment in tetrapods and fish. Genes predicted in the genome are represented by arrows and the arrowheads indicate the gene orientation. *C4* genes are represented by full- colored arrows: *C.4.1* in dark blue and *C.4.2* in light blue. Neighboring genes families are represented in different colors and by continuous (*C.4.1*) and dashed (*C.4.2*) outlines and homologue genes are indicated in the same color. The genes are mapped on chromosomes based on their actual positions (Mbp) predicted in the genome assemblies. Only genes that were conserved in the homologous genome regions in all species compared are represented. No homologue genome region of human *C.4* was found in the spotted gar genome. Steroid 21-hydroxylase (*CYP21A2*), Tenascin-X (*TNXB*), FRAS1-related extracellular matrix protein 2 (*FREM2*), Cyclic AMP-dependent transcription factor ATF-6 beta (*ATF6B*), FK506-binding protein-like (*FKBPL*), Epidermal growth factor-like protein 8 (*EGFL8*), 1-acyl-sn-glycerol-3-phosphate acyltransferase alpha (*AGPAT1*), Pre-B-cell leukemia transcription factor 2 (*PBX2*), G-protein-signaling modulator 3 (*GPSM3*). FRAS1-related extracellular matrix protein 2 (*FREM2*), Short transient receptor potential channel 4 (*TRPC4*), Periostin (*POSTN*), Dachsous cadherin-related 1a (DCHS1A), Arfaptin-2 (*ARFIP2*), Tripartite motif-containing protein 3 (*TRIM3*), Stomatin-like protein 3 (*STOML3*), LHFPL tetraspan subfamily member 6 protein (*LHFPL6*), Conserved oligomeric Golgi complex subunit 6 (*COG6*), and Forkhead box protein O1 (*FOXO1*).

**Figure 6:**
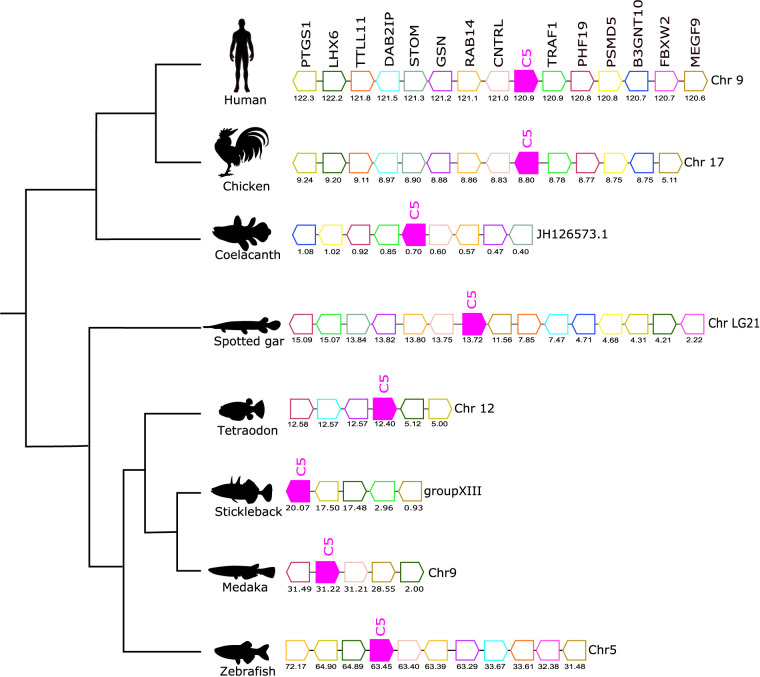
Gene synteny analysis of the *C5* gene environment in tetrapod and fish. Genes predicted in the genome are represented by arrows and the arrowheads indicate the gene orientation. Neighboring genes families are represented by different colors and homologue genes are represented by the same color. The genes are mapped on the chromosomes according to their predicted positions (Mbp). Only genes that were conserved in the homologous genome regions in all species compared are represented. TNF receptor-associated factor 1 (*TRAF1*), PHD finger protein 19 (*PHF19*), 26S proteasome non-ATPase regulatory subunit 5 (*PSMD5*), Hexosyltransferase (*B3GNT10*), F-box/WD repeat-containing protein 2 (*FBXW2*), Multiple epidermal growth factor-like domains protein 9 (*MEGF9*), Centriolin (*CNTRL*), Ras-related protein Rab-14 (*RAB14*), Gelsolin (GSN), Erythrocyte band 7 integral membrane protein (*STOM*), Disabled homolog 2-interacting protein (*DAB2IP*), Tubulin polyglutamylase TTLL11 (*TTLL11*), LIM/homeobox protein Lhx6 (*LHX6*), Prostaglandin G/H synthase 1 (*PTGS1*).

**Figure 7:**
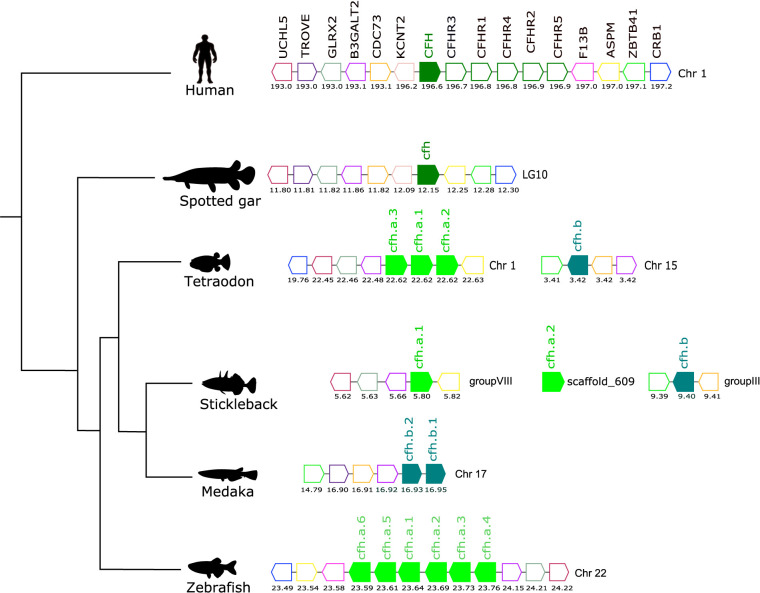
Gene synteny analysis of the *CFH/CFHR* gene environment in tetrapod and fish. Arrows represent genes predicted in the genome and arrowheads indicate the gene orientation. The vertebrate *CFH* genes are represented by full coloured arrows: tetrapod and spotted gar (olive-green) and the teleost duplicates, *cfha* (light-green), and *cfhb* (green-blue). Neighboring genes families are represented by different colours and homologue genes between species are represented by the same coloured arrow. The genes are mapped on chromosomes according to their predicted positions (Mbp). Only genes that were conserved in the homologous genome regions in all species are represented. Coagulation factor XIII B chain (*F13B*), abnormal spindle microtubule assembly (*ASPM*), zinc finger and BTB domain containing 41 (*ZBTB41*), crumbs cell polarity complex component 1 (*CRB1*), potassium sodium-activated channel subfamily T member 2 (*KCNT2*), cell division cycle 73 (*CDC73*), beta-1,3-galactosyltransferase 2 (*B3GALT2*), Ro60, Y RNA binding protein (*TROVE*), Ubiquitin C-Terminal Hydrolase L5 (*UCHL5*).

The gene environment of *C3*, *C4*, *C5*, and *CFH* genes in human was used as a reference to characterize the homologue genome regions in fish (**Figures 4**–**7**). 

#### Complement Genes

In humans, *C3* mapped to chromosome 19 and homologues of at least 11 neighboring genes were found in the genomes of several fish and in the lizard (**Figure 4**). In the human genome, a single *C3* gene persisted but in the lizard four *C3* gene copies were found and all genes mapped to chromosome 2, and three were in very close proximity suggesting that they resulted from a recent tandem duplication event. Many of the duplicate teleost *c3* genes were also arranged in tandem suggesting that they were the result of recent species-specific duplication events (**Figure 4**). In the zebrafish, *c3* genes were distributed on two chromosomes, the *c3.1* genes mapped to chromosome 1 while the *c3.2* genes mapped to chromosome 22. But in the tetraodon the three *c3.1* genes and the single *c3.2* gene mapped to chromosome 18 in close proximity and in stickleback *c3.2.1* was localized on the same chromosome as the *c3.1* genes (group IX). The results suggest that in the teleosts different genome rearrangements occurred for *c3.1* and *c3.2*. The other stickleback *c3.2* genes mapped to small genome fragments (scaffolds) that were poorly annotated. In medaka, three out of the five c3.1 genes found map in tandem with c3.2 on chromosome 1 and the two remaining c3.1s are localized in other chromosomes. The absence of conserved gene linkage for the spotted gar *c3.2* genome region (which maps to a short scaffold JH591472.1) meant gene origin could not be assigned.

The gene environment of *C4* was also conserved across tetrapods and the fishes and *CYP21A2* and *TNXB* genes mapped near to *C4.1* (**Figure 5**). In human, the two *C.4* genes (*C4.1.1* and *C4.1.2*, known as *C4A* and *C4B*) mapped in tandem to chromosome 6 but in the chicken the two *C4* genes were localized on different chromosomes and *c4.1* mapped to chromosome 16 and *c4.2*, which mapped to chromosome 1 had a unique gene environment. In coelacanth, tetraodon and stickleback a single *c4* gene copy existed and the neighboring gene environment was similar to that found in the human *C4* genes (**Figure 5**). In the medaka, the duplicate *c4* genes mapped in tandem (*c4.1.1* and *c4.1.2*) but in zebrafish, *c.4.1* and *c4.2*, mapped to different chromosomes. The spotted gar and zebrafish *c4.2* genome regions shared no gene homologues with the flanking region of the human *C4* genes or other teleost *c4.1* genome regions but they were similar to the flanking region of chicken *c4.2* (**Figure 5**). In human, although a homologue region of the vertebrate *c4.2* gene environment was found on chromosome 13 and also in several fish, tetraodon chromosome 16, stickleback group I, medaka chromosome 13 and elephant shark scaffold KI635872.1 the *c4.2* gene was not identified and was presumably deleted from their genomes (**Figure 5**).

The gene environment of *C5* was conserved across the different species analyzed (**Figure 6**). A homologous gene environment and gene order for at least eight human C5 flanking genes on chromosome 9, *TRAF1*, *PHF19*, *PSMD5*, *B3GNT10*, *CNTRL*, *RAB14*, *GSN*, and *STOM*, was found in fish. Furthermore, the *C5* gene was linked to the *CNTRL* gene in most of the genomes analyzed and this confirmed that in all vertebrates the *C5* gene shared a common origin (**Figure 6**).

### Complement Factor H Members

Comparisons of the gene environment of human *CFH* with ray-finned fish *cfh* regions revealed a conserved gene neighborhood and suggested they shared common ancestry (**Figure 7**). In human, the *CFH* gene mapped to chromosome 1 along with the five adjacent *CFHR* genes and formed a gene block. In fish a similar gene block was found on zebrafish chromosome 22 but in other teleost genomes the gene organization differed. In the tetraodon, three tandem *cfh* genes (*cfha*) were found on chromosome 1 but the fourth gene (*cfhb*) mapped to chromosome 15. In the stickleback the three *cfh* genes mapped to different genome regions. In medaka the two *cfh* genes (*cfhb1* and *cfhb2*) mapped in tandem on chromosome 17 (**Figure 7**). In addition, the teleost *cfha* genes were in linkage with *aspm*, *b3galt2*, *crb1* and *glrx2* genes and the teleost *cfhb* genes mapped in closed proximity to *trove*, *zbtb41* and *cdc73*, that neighbor human *CFH/CFHR* genes, and this provides further evidence that the extra copies of the *cfh* genes in teleosts resulted from the teleost specific whole genome duplication event (**Figure 7**).

### Sequence Comparisons

**Figure 8:**
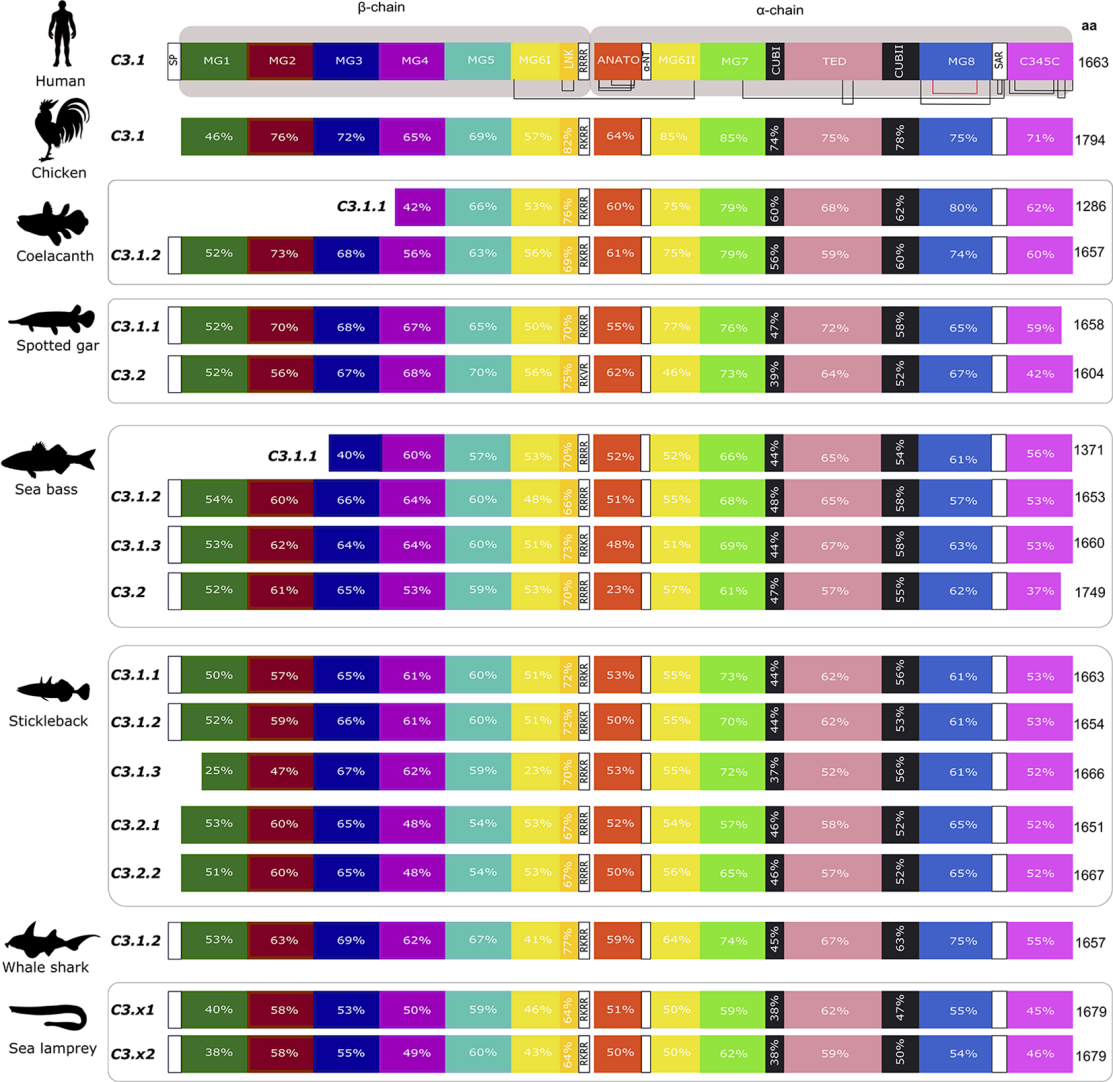
Structural comparison of the fish and tetrapod deduced C3 proteins. Thirteen protein domains were annotated according to the crystallographic structure of human C3 (48) and homology modelling of the predicted structure of an Antarctic teleost C3 (32) and they are represented by blocks with different colors. The two human chains, β- (residues 1–645 aa) and α- (residues 650–1641 aa) are indicated. The homologue regions in fish and chicken were identified using a multiple sequence alignment (**Supplementary Figure 3**). The localization of 13 disulphide bridges in the human C3 are indicated and the position of the 24 cysteine residues are generally conserved in other vertebrate C3’s with the exception of one disulphide bridge within MG8 (annotated in red) that was only maintained in chicken, the coelacanth C3’s, spotted gar C3.2 and in the lamprey C3 sequences (**Supplementary Figure 3**). The percent similarity between the human domains and the homologue regions in other vertebrates is indicated as well as the homologue residues of the four arginine motif (4ARG). The low sequence similarity of the sea bass C3.2 ANATO domain with human is due to the larger length of the fish sequence. The predicted total size (aa) of the different C3 proteins is indicated. SP, signal peptide, MG- macroglobulin domain (MG1-8); LNK, link domain; ANATO, anaphylatoxin domain; α-NT, N-terminal region of the cleaved α-chain; CUB, complement C1r/C1s, Uegf, Bmp1 domain; TED, thioester-containing domain; SAR, short anchor region; C345C, carboxy-terminal domain. A SP was not predicted in some of the sequences.

**Figure 9:**
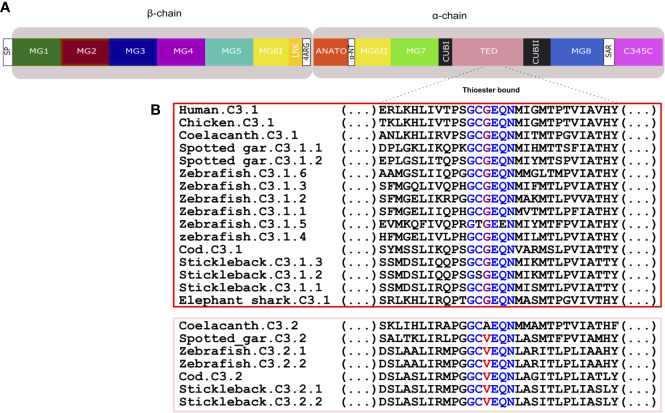
Comparison of the fish and human C3 thioester bond. **(A)** Schematic representation of the thirteen conserved domains of human C3. **(B)** Sequence comparison of the fish, human and chicken C3 thioester bond responsible for attaching the complement proteins to the surface of the pathogen. The six aa motif forming the thioester bond are colored and conserved aa are denoted in the same color. The C3.1 (red) and C3.2 (pink) sequences were grouped to better illustrate the differences within this region. SP, signal peptide; MG, macroglobulin domain (1-8); LNK, link domain; ANATO, anaphylatoxin domain; α-NT, N-terminal region of the cleaved α-chain; CUB, complement C1r/C1s, Uegf, Bmp1 domain; TED, thioester-containing domain; SAR, short anchor region; C345C, carboxy-terminal domain.

**Figure 10:**
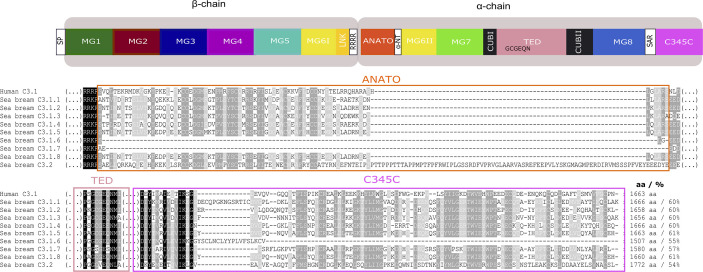
Comparison of the gilthead sea bream C3 isoforms with the human C3. The protein domains in which most differences were found ANATO, TED, and C345C and may cause functional differences between the gilthead sea bream and human and within the gilthead sea bream C3 paralogues are represented. Symbol description is available in the legend of **Figure 8**. The multiple sequence alignment of the full protein is available as **Supplementary Figure 6**. Sequence conservation is denotated by background color gradient (black, total conservation; white, no conservation). The percent of similarity between the nine gilthead sea bream C3 isoforms with human C3.1 is indicated.

**Figure 11:**
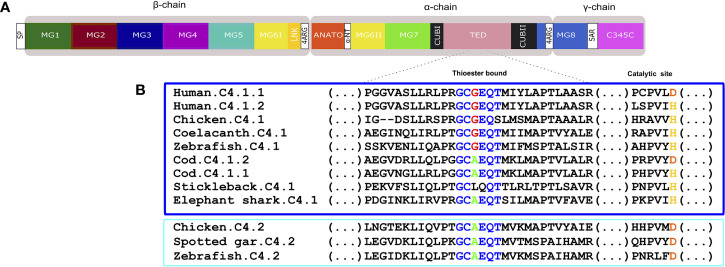
**(A)** Schematic diagram of the thirteen conserved domains including the additional tetra-arginine (RRRR) processing site within MG8 in human C4. **(B)** sequence alignment of the fish, human and chicken C4 thioester bond that is responsible for attaching the complement proteins to the surface of pathogens. The six aa motif forming the thioester bond are colored and conserved aa are denoted in the same color. The C4.1 (dark blue) and C4.2 (light blue) sequences were grouped to better illustrate the differences within this region. The catalytic site is also represented as duplicate vertebrate C4 genes were previously classified based on the substitution of the normally conserved histidine (H) in C4.1 (named as c4-H-type) by an aspartic acid (D) in C4.2 (named as c4-D type) (54).

Sequence comparison between the deduced complement proteins revealed that the fish proteins shared high sequence similarity and conserved functional and structural domains with human homologues (**Figures 8**–**11** and **Supplementary Figure 3**–**5**). The fish complement proteins encoded by duplicate genes also shared a well conserved structure (**Figure 10**, **Supplementary Figure 6**).

The cartilaginous fish C3, C4, and C5 shared 61%–65 %, 52%–54 %, and 65% aa similarity with the human homologues, respectively. The spotted gar C3s shared 64%–67% aa sequence similarity with human C3 while C4 and C5 were less conserved (53% and 58% aa similarity, respectively). Considering only the teleosts a common pattern of aa sequence similarity was found for complement isoforms and is exemplified by the stickleback. The stickleback C3s shared approximately 54% aa similarity with the homologue forms of human complement but the duplicate stickleback C3.1 and C3.2 were only 48% similar to each other, although the C3.1.1 and C3.1.2 isoforms were highly conserved (94% aa similar). The teleost C4 shared approximately 59% aa similarity with human C4 and the duplicate C4.1 and C4.2 isoforms in zebrafish shared 52% aa similarity. The C5 isoform in teleosts shared approximately 60% aa similarity.

#### The fish C3

Thirteen major domains (six within the β-chain, six within the α-chain and one shared between both chains) were characterized in human C3 (48) and homologue conserved regions were found in C3 across the fish (32) (**Figure 8**). The tetra-arginine motif (4ARG, RRRR) is a proteolytic cleavage site that is localized at the end of the β-chain and separates the β-chain from the α-chain during post-translational processing. In human C3, the β-chain consisted of five complete α2-macroglobulin domains (MG1-5) and an α-chain that included an anaphylotoxin-like domain (ANATO), two complete MG domains (MG7 and MG8), a thioester domain region (TED), two parts of a CUB domain (CUBI and CUBII) localized at both sides of the TED domain and the C345C domain which contains the netrin region at the C-terminus of the sequence (**Figure 8**). A sixth MG domain (MG6) was shared between the β-chain and the α-chain (MG6I and MG6II). The deduced fish C3.1 and C3.2 proteins had a similar organization and homologue domains of human C3 exist and they are relatively well conserved in terms of sequence. In general, between human and fish C3, the LNK, MG7 and TED domains are the most conserved domains and MG1, ANATO, and C345C domains are the most variable (**Figure 8**). The 3D structure of human C3 is stabilized by 13 disulphide bonds formed between 26 cysteines, 24 of which are located between MG6I and C345C and have been conserved in fish. The exception is for the cysteines in MG8 that form a disulphide bond in human (**Figure 8** annotated in red, **Supplementary Figure 3**) and, which were only conserved in the coelacanth C3.1.1 and C3.1.2, spotted gar C3.2 and in the lamprey C3.x1 and C3.x2 sequences. A signal peptide was predicted in most of the sequences suggesting these are secreted but in others remain to be identified and they may have different cellular localizations. The TED domain contained a highly conserved thioester region that is responsible for attaching the complement proteins to the surface of the pathogen (17). In human, the thioester region contains a six aa motif, GCGEQN, which is totally conserved in the C3.1 proteins from cartilaginous fish, ray-finned fish and coelacanth (**Figure 9**). In C3.2 of the ray-finned fish, the third Gly (G) is mutated to Val (V), suggesting that functional divergence may have occurred between the gene duplicates (**Figure 9**). Moreover, zebrafish C3.1.5 contained two aa mutations and stickleback C3.1.2 contained a single aa modification suggesting that functional divergence may also occur between the species-specific duplicates.

### The Gilthead Sea Bream C3 Isoforms

In the gilthead sea bream, the C3.1 forms are highly similar and C3.1.5 and C3.1.8 shared the highest aa similarity (97%). The C3.2 form in gilthead sea bream shared approximately 51.7% aa similarity with the C3.1 isoforms. The shortest C3.1 form was C3.1.6 (1507 aa) but all other C3 isoforms (C3.1 and C3.2) had a similar size to human C3 with the exception of C.3.2 which was longer (1772 aa).

For gilthead sea bream C3.1, in five isoforms, C3.1.1, C3.1.2, C3.1.3, C3.1.5, C3.1.6, and C3.1.8 the thioester region identified in human C3 was conserved, but C3.1.4 and C3.1.7 contained aa mutations and C3.2 differed and was identical to C3.2 in other ray-finned fish (**Figure 10**, **Supplementary Figure 6**). Differences within other domains were also identified; C3.1.6 and C3.1.7 lacked a full-length ANATO domain; C3.2 had a longer ANATO domain (81 aa longer) compared to C3.1 in human and other fish; the 4ARG region that precedes the ANATO domain in human was conserved in gilthead sea bream C3.2 but modified to RRKR or RKKR in C3.1; C3.2 had a longer MG1 domain compared to the human and other gilthead sea bream C3s (**Supplementary Figure 6**). Similar to other teleosts, 24 cysteines located between MG6I and C345C were also conserved in the gilthead sea bream C3s. Additional, cysteine residues only conserved in the gilthead sea bream C3s were within MG1, MG6II and TED (**Figure 10**, **Supplementary Figure 6**). N-glycosylation sites were identified in MG7, CUB2, and MG8 domains and were conserved in other fish but not human (**Supplementary Figure 6**).

### Fish C4 and C5

Fish C4 had a similar protein organization to C3 and the predicted full-length C4 sequences were similar to human C4 (**Figure 11A**, **Supplementary Figure 4**). The deduced C4 proteins from cartilaginous fish through to tetrapods all possessed the thirteen major functional domains (**Figure 11A**, **Supplementary Figure 4**). The two homologue regions of the human C4 tetra-arginine (RRRR) processing sites, one after LINK domains and the other within the MG8 domains were also present in the fish sequences. In the teleost C4 proteins, a signal peptide sequence was predicted with the exception of the stickleback C4.1 (**Supplementary Figure 4**). The aa sequence of the C4 thioester domain, GCGEQT, in human was conserved in coelacanth and zebrafish C4.1. In other ray-finned fish and in the elephant shark the second Gly (G) was mutated to Ala (A) but more extensive changes existed in the stickleback C4.1 sequence (**Figure 11B**). The aa sequence of chicken C4.1 and C4.2 only differed at the last aa (S and T, respectively) and the C4.2 thioester motif was identical to the spotted gar and zebrafish C4.2. Within the catalytic domain the normally conserved C4.1 histidine (H), involved in the cleavage of the thioester to bind the target cells, was replaced by an aspartic acid (D) in C4.2. For C5 the deduced protein structure in fish and tetrapods was similar and no thioester domain existed (**Supplementary Figure 5**).

### Expression of the Complement System in Teleost

To assess the specialization of the complement system in fish the expression of *c3*, *c4*, and *c5* gene transcripts as well as other members of the complement system and *cfh* was assessed in teleost skin by analyzing available transcriptomes.

The sea bass (pelagic, temperate), rockcod (demersal, polar) and Senegalese sole (demersal, temperate) occupy different ecological niche presumably with different microbiota and it was hypothesized that this might be reflected by differences in complement expression in the skin. Analysis of available transcriptome data for sea bass, Senegalese sole and black rockcod and other fish species revealed that fewer components of the complement system were expressed in the sea bass skin relative to the other two species, although this is most likely linked to sequence coverage (**Table 3**). All three species expressed complement transcripts and *c4.1*, *c6*, and *c7b* were present in all the explored skin transcriptomes. Transcripts for *c3*, *c5*, *c8*, and *cfh* were found in the black rockcod and Senegalese sole. In the sea bass transcripts for *c1q* and *c1s* were identified and in the black rockcod only *c1s* was identified and in the sole only *c1q* was detected. Transcripts for several different *c3* genes were detected in the skin of the rockcod. Different transcripts of *cfh*, *cfh.1*, and *cfh*.*2* were detected in rockcod skin and a single form, *cfh.1*, was found in Senegalese sole skin and no *cfh* transcripts were found in sea bass skin (**Table 3**).

**Table 3:** Expression of complement system in teleost skin.

Complement component	*Sea bass*(*Dicentrarchus labrax*)	Black rockcod(*Notothenia coriiceps*)	Senegalese sole(*Solea senegalensis*)
*c1q* (a/b/c), *c1s*, *c1r*	*c1qb, c1s*	*c1s*	*c1qa*
*c/b2*	–	*c/b2*	–
*c3* (1/2)	*-*	*c3.1, c3.2*	*c3.1*
*c4* (1/2)	*c4.1*	*c4.1*	*c4.1*
*c5*	–	–	c5
*c6*	*c6*	*c6*	*c6*
*c7* (a/b)	*c7b*	*c7a, c7b*	*c7a, c7b*
*c8* (α/β/γ)	–	*c8γ*	*c8α, c8β, c8γ*
*c9*	–	–	–
*cfh* (a/b)	–	*cfha, cfhb*	*cfhb*
Assembled contigs	31.858	848.752	50.113

RNA-seq transcriptome (Illumina platform) assemblies of the European sea bass skin (GFJW00000000) (50), Senegalese sole skin (PRJEB29449) (51) and the de novo transcriptome assembly of the Antarctic black rockcod (Notothenia coriiceps) were analyzed. The number of assembled contigs in each library is given.

**Figure 12:**
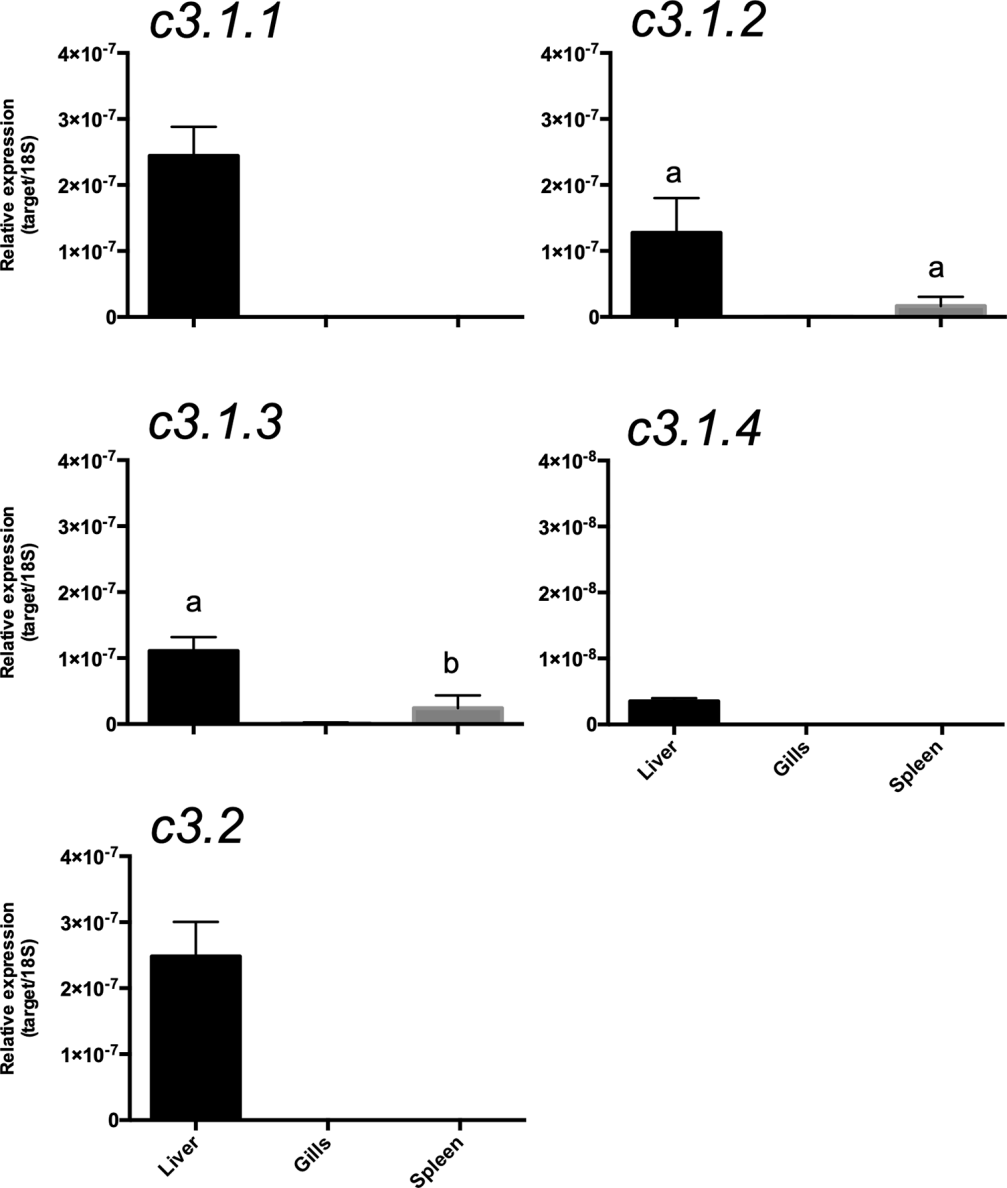
Tissue distribution of the *c3* transcripts in the gilthead sea bream (*Sparus aurata*). Gene expression levels were determined by quantitative PCR and normalized using *18s* as the reference. The results are represented as the mean ± SEM of three (n= 3) biological replicates. One-way Anova and Tukey’s multiple comparison test was used to identify significant differences in transcript abundance using Prism GraphPad v5 software. Bars with different letters are significantly different (p < 0.05).

To assess if multiple gene isoforms of *c3* were retained due to functional specialization linked to divergent tissue distribution, their expression pattern in immune-related tissue of the gilthead sea bream was characterized. Overall, *c3.1.1*, *c3.1.2*, *c3.1.3*, *c3.1.4, and c3.2* expression was mainly restricted to the liver and they were of very low abundance or undetectable in the spleen, gills or skin of the gilthead sea bream (**Figure 12**). In liver, *c3.1.1* and *c3.2* were most abundant and the least abundant was *c3.1.4*. Low but detectable levels of *c3.1.2* and *c3.1.3* transcripts were detected in the spleen.

## Discussion

The immune system in fish is proposed to have contributed to their evolutionary success in the face of innumerable aquatic pathogens (55). The expansion of *c3* and *cfh* genes in teleosts suggests that the alternative complement pathway was favored during evolution. The direct activation of *c3* by pathogens in the environment and the protection this confers may partly explain why teleosts are the most successful of the extant vertebrates (39). Overall, the results suggest that a more complex complement system and innate response evolved in the fish that inhabit a microbial rich aquatic environment compared to terrestrial vertebrates (56, 57) and this potentially increased their capacity to recognize a broader spectrum of pathogens.

**Figure 13:**
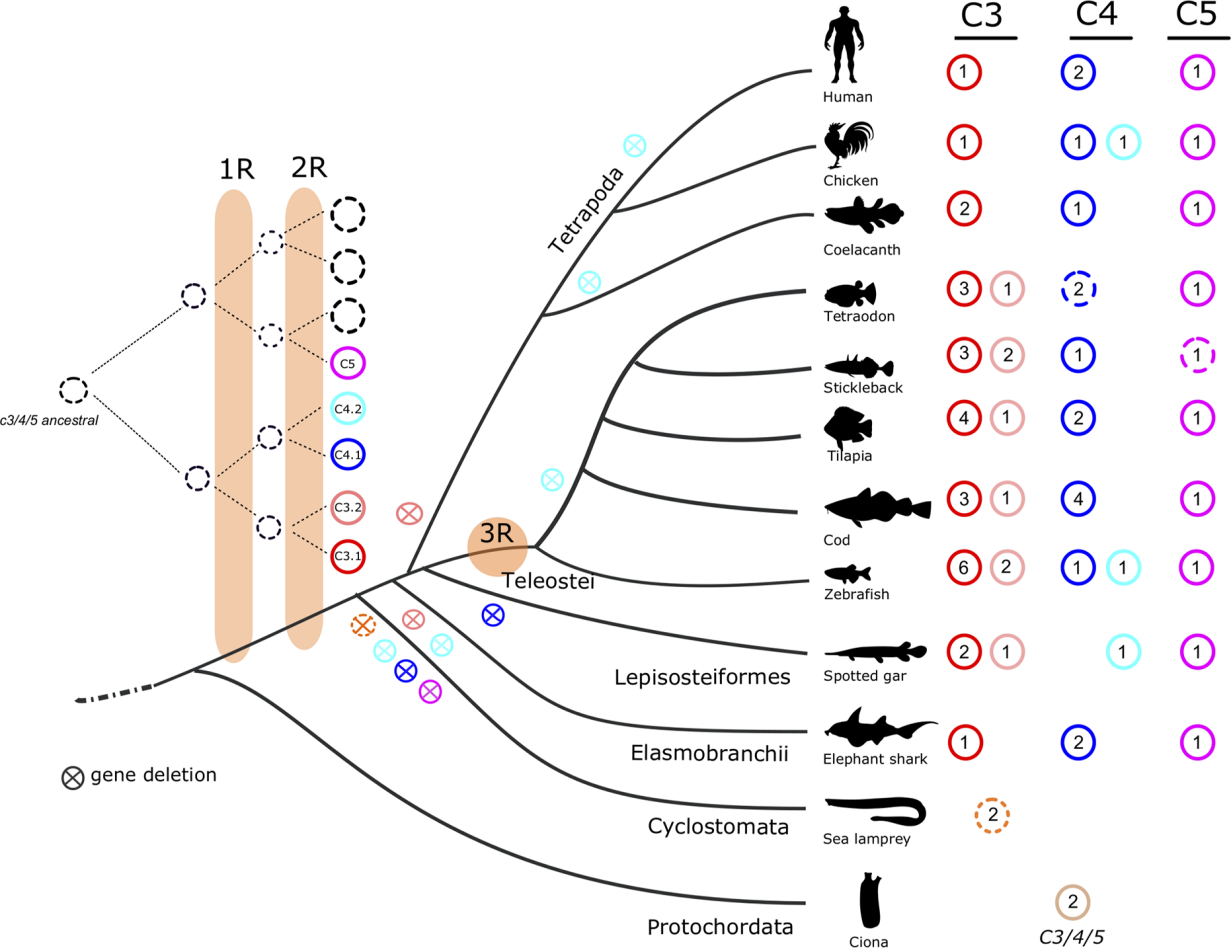
Proposed model for the evolution of the vertebrate *C3*, *C4*, and *C5* genes. The number of full-length *c3* (*c3.1* in red; *c3.2* in pink), *c4* (*c4.1* in dark-blue; *c4.2* in light-blue), and *c5* (purple) genes are presented with colored circles. Open circles indicate incomplete sequences. The cross within the circle signifies absence or elimination during evolution. The reconstruction model suggests that vertebrate *c3*, *c4*, and *c5* evolved from two independent ancestral molecules prior to the vertebrate radiation and that the duplicate vertebrate *c3* and *c4* genes resulted from the two rounds of genome tetraploidization (1R and 2R). The teleost specific genome duplication is indicated by 3R. The lamprey *c3* sequences are represented by a dashed orange circle as homology for gnathostome *c3.1* and *c3.2* is uncertain. The existence in tunicate (basal deuterostome) of *c3/c4/c5*-like molecules suggest that duplication of *c3/c4* and *c5* ancestral molecules occurred prior to the 1R and 2R.

The evolution of vertebrates was preceded by two successive major genome tetraploidization events 1R and 2R (57, 58) which contributed to increase gene diversity and genome complexity. These genome duplication events occurred very early at the vertebrate origin and most likely before the cyclostome-gnathostome divergence (59–61). Our phylogenetic and gene neighborhood analysis suggests the fish *c3* genes shared a common ancestral origin with the human and other tetrapod genes. The identification in the spotted gar (*c3.2*) of the duplicate family members previously proposed to be teleost specific (30) suggests that *c3* gene duplication occurred earlier than previously thought potentially at the root of the vertebrate radiation and only persisted in the ray-finned fish (**Figure 13**). In teleost genomes both *c3.1* and *c3.2* genes were found and the larger number of *c3.1* genes retrieved in relation to *c3.2* suggests they evolved at different rates within each species. The evolution of the duplicate *c3* genes is currently difficult to resolve due to their species-specific evolution and lack of conserved gene linkage. In lamprey and hagfish duplicate *c3* genes were also found and our phylogenetic analysis revealed that they radiated basal to *c3.1* and *c3.2* in the ray-finned fish. However, gene orthology between cyclostomes and gnathostome genes is uncertain due to the selective pressure under which lamprey and hagfish genomes have been evolving especially within the gene coding regions (62, 63).

The multiple gene copies of the teleost *c3.1* are arranged in tandem in the genomes of some species or in different chromosome region in others, suggesting gene evolution was distinct and probably a consequence of species-specific chromosome rearrangements (64). In zebrafish, in which the *c3* duplicates have previously been characterized the two *c3.2* proteins were associated with the regulation of the proinflammatory response, while the six *c3.1* copies had a different response (30). In general the predicted C3 proteins in teleosts are of a similar size to human C3, as revealed by the *c3* transcripts previously isolated from the rainbow trout and zebrafish and in the present study from gilthead sea bream (25, 26, 30). In the common carp (*Cyprinus carpio*) where multiple C3 isoforms have also been described functional divergence of their catalytic activities was reported (27). One C3 variant (C3-Q2) lacks most of the beta-chain and is not functional (27) and this phenomenon may be common in teleosts since many of the species analyzed in the present study had incomplete *c3* genes. Further work will be required to better characterize the incomplete *c3* genes (pseudogenes or genome assembly errors) and to better establish the functional diversity of the *c3* gene and protein complex in teleosts.

The gilthead sea bream had nine *c3* gene transcripts highly expressed in liver although as reported in other species, extra-hepatic expression also occurs (30, 65, 66). Sequence comparison of the gilthead sea bream *c3* isoforms revealed they have a similar length and structure, although C3.1.7 lacked the anaphylatoxin domain and C3.1.6 lacked both the anaphylatoxin domain and had a shorter C345C domain. These characteristics taken together with the different number of conserved cysteine residues and N-glycosylation sites in C3 isotypes emphasizes their potentially different structure and function. This idea is reinforced by studies of C3 proteins in gilthead sea bream plasma and five C3 isotypes were reported that were functionally and structurally divergent (67). Comparison of the gene transcripts identified in the present study with the short N-terminal sequence for the C3 plasma protein previously reported indicated they are likely to correspond to C3.1.1 (C3-3) and C3.1.2 (C3-4) and the remaining three proteins, which included the most abundant, C3-1 and C3-2 proteins, had in general the greatest similarity with our C3.1.3, C3.1.5 and C3.1.8 transcripts and it was not possible to assigned sequence correspondence. Previously published *c3* gene expression studies in immune challenged sea bream (**Table 4**) have assessed sea bream *c3.1.5* (HM543456 and CB184637) and *c3.1.8* (CX734936), that were not analyzed in our study. The sometimes-contradictory results reported for *c3* gene transcripts may be explained by differences in the *c3* gene amplified. The number of *c3* isoforms with distinct structures and expression in gilthead sea bream as well as in other teleost species highlights the need for further in-depth studies of *c3* in fish.

**Table 4:** Expression pattern determined by qPCR of the different gilthead sea bream c3 isoforms obtained in the present study compared with previous studies.

Gilthead sea bream c3 isoforms									
*c3.1.1*	*c3.1.2*	*c3.1.3*	*c3.1.4*	*c3.1.5*	*c3.1.6*	*c3.1.7*	*c3.1.8*	*c3.2*	*Ref.*
Li	Li, Sp	Li, Sp	Li	nd	nd	nd	nd	Li	Present study
–	–	–	–	–	–	–	Li, In, Hk	–	(68)
–	–	–	–	Go	–	–	–	–	(69)
–	–	–	–	–	–	–	Li, Sp, In, Hk	–	(70)
–	–	–	–	Li, In, Br	–	–	–	–	(71)
–	–	–	–	–	–	–	Li, In, Hk, Sk	–	(72)
–	–	–	–	–	–	–	Hk, In	–	(73)
–	–	–	–	–	–	–	Go	–	(74)^☐^
–	–	–	–	–	–	–	Sp,Sk	–	(75)^‡^
–	–	–	–	In, Gi, Sk	–	–	–	–	(66)^ʠ^
–	–	–	–	Li, Sp	–	–	–	–	(76)
				In, Hk			In, Hk		(77)^?^

Correspondence between the primers used to amplify c3 in previously published studies and the nine gilthead sea bream sequences identified in the present study. Tissues in which PCR amplification of c3 were detected liver (Li): spleen (Sp), intestine (In), head-kidney (Hk), gonad (Go), brain (Br), skin (Sk), gill (Gi).

nd: Not determined.

^☐^in vitro treatment with poly I:C.

^‡^injection with lysate and ToxA.

^ʠ^after biotic (Vibrio anguillarum bacterin), abiotic (air exposure), or combination of both stressors.

^?^Primers may amplify both c3.1.5 and c3.1.8.

The variable number of *c3* genes identified in our study and their different organization in teleost genomes suggests that they probably evolved under different pressures. The results of our study indicate that maintenance in the genome of multiple *c3* gene copies in teleosts is unlikely to be due to their divergent tissue distribution. However, variation in the sequence and structure of the deduced teleost C3 proteins and the functional diversity demonstrated in previous studies has presumably favored their maintenance in the genome. Furthermore, since the bacterial cell wall triggers the hydrolysis of C3 and activation of the alternative complement pathway, we hypothesize that the high diversity of microorganisms in aquatic systems potentially increased the pressure on *c3* isoform evolution in fish (78–83).

The existence of duplicate *c4* genes in fish and chicken suggests that gene duplication occurred prior to their divergence but that the duplicate *c4.2* gene did not persist in all fish species (**Figure 13**). Duplicate *C4* genes have previously been described in vertebrates including fish (54, 84) and were named C4 H-type and C4 D-type based on the replacement within the catalytic site of the conserved histidine (H) by an aspartic acid (D) (54). Both genes were proposed to result from two independent duplications: one in the cartilaginous fish lineage and a second in the bony vertebrate lineage that was followed by species-specific duplications in mammals (54). By analyzing the C4 gene environment from cartilaginous fish to human, an alternative evolutionary scenario was established in which C4.1 (previously known C4-type H) and C4.2 (previously known C4-type D) arose from a single gene/genome duplication that occurred early during the vertebrate radiation and prior to the gnathostome divergence (**Figure 13**). In the present study the persistence of both *C4* genes occurred in the genome of very few species and generally only the *C4.1* gene copy was retained and subsequently duplicated in a species-specific manner.

The insightful study of toll-like receptor (Tlr) evolution in cod (85), proposed that the acquisition of multiple isoforms was related to paleoclimatic events. By mapping the Tlr repertoire of 76 teleosts onto a time calibrated species phylogeny it was found that in Gadiformes, selection was towards a higher copy number optima for some, but not all the Tlrs. It was proposed that highly variable paleoclimatic arctic conditions and variable pathogen loads and pathogen community composition might explain Tlr gene evolution in Gadiformes. In contrast to the Tlr analysis, in our study the *c3* genes from Arctic Atlantic cod and Antarctic marbled and black rockcods, did not undergo large copy number expansion as occurred in other teleosts. Instead *c3* gene copy number tended to be higher in teleosts from warmer waters (tropical and subtropical regions). Based on our results it was difficult to identify a clear association between temperature and complement system evolution in the fish unlike was proposed for TLR evolution in Gadiformes. Nonetheless, in general the ectotherms had multiple *c3* genes (e.g. ray-finned fish, amphibians and reptile) compared to the single *c3* genes retrieved from the chicken and human genomes and further studies will be required to explain the basis of this difference. The other complement system members do not appear to be under the same evolutionary pressure as *c3* since gene copy number was similar between the different fish species analyzed (**Figure 1**). The alternative complement pathway is reported to be at least 60 times more active in teleosts than in mammals (38) and we hypothesize that this may be explained by the multiple *c3* genes and their protein products present in fish. The high C3 gene family expansion in fish relative to other vertebrates was also accompanied by an expansion of the regulatory protein Cfh and it will be of interest in the future to establish if specific partnerships between C3 forms and regulatory Cfh isoforms have arisen.

The importance of innate immunity in teleosts is not only associated with *c3* duplications but also with the expansion of other innate immune related genes including the toll-like receptors, NOD-like receptors (NLRs), RIG-I like receptors (RLRs), antimicrobial peptides (AMPs), lectin proteins, and interferons (INFs) (86–88). The functional significance of the species-specific *c3* gene duplication events identified in teleosts was not established but may be linked to the dominant role of the innate immune response (86). This notion seems to be supported by the less abundant *c3* species-specific duplications in cartilaginous fish (only two *c3* duplicates were found in whale shark and a single gene was retrieved from the elephant shark genome) in which alternative adaptive immune strategies have evolved (89). In fact, the elasmobranchs have a 10-times higher immunoglobulin repertoire, associated, in part, with the process of affinity maturation, when compared with teleosts, which have a relatively minor secondary antibody response (90–92). We hypothesize that the large expansion of the *c3* genes in teleosts may have compensated for their weaker secondary antibody response. This also presumably explains why the titre of the alternative complement pathway is several orders of magnitude higher in teleosts than in mammals (78, 93). Furthermore, the broad spectrum of recognition and activation of teleost C3 by red blood cells from a diversity of organisms has been linked to the differing specificity of the multiple C3 isoforms (93). The higher number of *c4* genes in cartilaginous fish relative to teleosts is intriguing. Multiple *C4* genes also exist in human and chicken that only have a single *C3* gene and it may be that in these species the classical or MBP-lectin complement pathways is of greater importance.

Adaptation of fish to multiple environments favors their contact with a diversity of pathogens and fish skin plays an important role in maintaining physiological homeostasis as it is the first physical barrier that protects against pathogens. Many immune factors including immunoglobulins (94, 95), lectins (96), antimicrobial peptides (97, 98), C-reactive proteins (98), proteases (100, 101), lysozymes (102), transferrin (TF) (95) and also several components of the complement system (95, 103–110) are secreted into the skin mucous and establish a specific chemical barrier (111–113). The contribution of complement to the protective function of the skin is clear and previously published studies aimed at characterizing the overall contribution of fish skin and mucous to immunity and the pathogen response revealed that members of the alternative (C3), classical (C1, C2, C4), and lytic (C5, C6, C7) pathways in skin are modulated by parasites, microorganisms and stressful conditions (**Table 5**). The control skin transcriptome data used in the present study revealed differences in complement pathway representation in adult sea bass, rockcod and sole (demersal, mud dwelling). However, the lower complement gene representation (e.g. *c3*, *c5*, *c8*, *c9* were missing) in the sea bass skin transcriptome is unlikely to be of biological relevance and is most likely due to the characteristics of the sequencing dataset, particularly since *c3* has previously been reported in the sea bass skin proteome (107). The generalized presence of the three complement cascade pathways in fish skin suggests they may be relevant biomarkers to assess the impact of the environment on teleost immune physiology.

**Table 5:** Complement system members in published transcriptome and proteome data available for teleost skin.

Complement	Teleost species	Study	Conditions	Reference
C4, C5, Bf/C2	*Cyprinus carpio*	Expression analysis	–	(103)
C1(q r, s),, C3, C4-B, C6, C7, C8 (α, β, γ), C9	*Larimichthys crocea*	Skin mucus proteome	Air exposure	(108)
C3, C1q	*Dicentrarchus labrax*	Skin mucus proteome	–	(107)
C3	*Salmo salar*	Infected skin proteome	Infected with sea lice *(Lepeophtheirus salmonis)*	(104)
B/C2, C3,C5, C6, Factor B	*Salmo salar*	Epidermal mucus proteome	Infected with sealice (*Lepeophtheirus salmonis*)	(105)
C3, C9	*Salmo salar*	Skin mucus proteome	Infected with amoebic gill disease	(106)
C3	*Oncorhynchus mykiss*	Skin mucus		(114)
C1s C2, C3, C4, C5, C7, C8(α, β, γ), C9	*Epinephelus coioides*	Skin transcriptome	Infected with *Cryptocaryon irritans*	(115)
C1q	*Sparus aurata*	Skin mucus proteome	–	(109)
C1, C3, C4, C5, C6, C7, C8	*Salmo trutta*	Skin transcriptome	–	(110)
C3	*Sparus aurata*	Skin mucus proteome	–	(116)

## Conclusion

Herein we provide a comprehensive evolutionary analysis of the complement system in teleosts compared to other vertebrates. Our phylogenetic analysis corroborates and extends previous *in silico* studies in which the early radiation of C5 and the later divergence of C3 and C4 was proposed based on a limited number of vertebrate species and the resemblance of the hagfish sequence to vertebrate C3/C4 (117). Our study confirmed that *c3* and *c4* shared common ancestry prior to the vertebrate radiation and that the duplicate *c3* and *c4* genes identified potentially arose during the two rounds of vertebrate genome duplications. In basal deuterostomes and other invertebrates putative *c3*/*c4*/*c5* genes exist that diverged from the same ancestral molecule as the vertebrate members (118–120). Our results based on sequence clustering confirm the standing hypothesis that the ancestral *c3*/*c4*/*c5* gene duplicated prior to the origin of the vertebrates which led to the emergence of the vertebrate *c5* ancestral gene and *c3/c4* ancestral genes (**Figure 13**). Both molecules expanded during the two rounds of genome tetraploidization (1R and 2R) and one originated the vertebrate *c5* and the other the *c3* (*c3.1* and *c3.2*) and *c4* (*c4.1* and *c4.2*) gene members which subsequently underwent distinct lineage and species-specific gene duplication and deletion events. In our analysis the previously identified hagfish and lamprey sequences grouped within the vertebrate C3 cluster suggesting C4 and C5-related forms do not exist (121).

A large expansion of the *c3* genes as well as the main regulatory factor of the alternative pathway, *cfh* was identified in all ray-finned fish genomes probably associated with lineage and species-specific duplications suggesting that the complement cascade in teleosts is more complex than in terrestrial vertebrates and that a variety of parallel pathways may exist probably to compensate for the less developed acquired immune response. Tissue expression of complement in the gilthead sea bream indicated that in common with other vertebrates duplicate *c3* genes are primarily expressed in the liver. The common tissue distribution of the *c3* gene transcripts in sea bream suggests that their maintenance in the genome is probably due to functional divergence as revealed by differences in their sequence and structure. The expression of multiple gene transcripts for the *c3* gene in teleosts, highlights the need for new studies to evaluate the response and role of the multiple genes in fish. To conclude, our study is the first comprehensive study that provides a detailed description and comparative evolutionary analysis of C3/C4/C5 and a regulatory factor of the alternative pathway, *cfh*, in deuterostomes with specific attention to fish. Furthermore, the expression and sequence analysis of the multiple C3 isoforms in teleosts highlights the need for functional studies to understand their role and explain why they persisted in the genome.

## Data Availability Statement

All datasets presented in this study are included in the article/**Supplementary Material** or are available in public repositories.

## Ethics Statement

All samples used were already available in the context of previous studies for which ethical approval was obtained.

## Author Contributions

DMP planned the study. BN and JCRC collected the data and performed the bioinformatic analyses. BN, JCRC, AVMC, and DMP integrated and discussed the datasets. BN, JCRC, and DMP wrote the manuscript. All authors contributed to the article and approved the submitted version.

## Funding

This work was financed by the European Union Horizon2020 Programme (PERFORMfish, grant n° 727610) and the Portuguese Foundation for Science and Technology (FCT) project to CCMAR (UIDB/04326/2020).

## Conflict of Interest

The authors declare that the research was conducted in the absence of any commercial or financial relationships that could be construed as a potential conflict of interest.

## Supplementary Material

Supplementary Table 1 Species names, accession numbers and genome localization of the fish and other vertebrate C3, C4, C5, and C1, C2, C6–C9 genes. Genes were named based on phylogenetic clustering (**Supplementary Figures 1**-**3**) and conservation of the gene environment (**Figures 4**-**6** and annotations available from the species genome assemblies).

Supplementary Figure 1 Phylogenetic trees of the fish C1, C2, C6-C9 genes. Accession numbers of the sequences are available in **Supplementary Table 1**. The trees were built with the BI method and posterior probability values are shown.

Supplementary Figure 2 Phylogenetic tree of the fish C3, C4 and C5 members with other vertebrates. The tree was built with the ML method and bootstrap branch support values are shown. The two major C3 clades were named C3.1 and C3.2 and duplicated genes for each species were numbered. The two C4 forms were named C4.1 and C4.2. Tree was rooted with the deduced protein sequences from human α-macroglobulin (A2M, ENST00000318602.12) and cluster of Differentiation 109 (CD109, ENST00000437994.6). Sequence accession numbers are available in **Supplementary Table 1**.

Supplementary Figure 3 Multiple sequence alignment of the vertebrate C3 deduced proteins. The thirteen protein domains were annotated according to the crystallographic structure of human C3 (48) and homology modelling of the predicted structure of an Antarctic teleost C3 (32). SP- signal peptide (highlighted in bold), MG- macroglobulin domain (MG1-MG8); LNK-link domain; ANATO- anaphylatoxin domain; α-NT- N-terminal region of the cleaved α-chain, CUB- complement C1r/C1s, Uegf, Bmp1 domain; TED- thioester-containing domain; SAR- short anchor region; C345C- carboxy-terminal domain. The cysteine residues are highlighted in yellow and the small, colored triangles of the same color represent a predicted disulphide bond for human C3.1 available from Uniprot. The conserved position of putative N-glycosylation sites (N-x-T/S, where x represents any amino acid) are highlighted in green. Sequence conservation is denotated by background color gradient: black- total conserved; white- no conservation. A schematic representation is available in **Figure 2**.

Supplementary Figure 4 Schematic representation of the predicted structural domains of C4 proteins (full-length and incomplete) from selected fish (spotted gar; three teleost’s the stickleback, sea bass, zebrafish; coelacanth; whale shark). The human and chicken sequences were included for comparisons. The thirteen conserved protein domains are annotated based on the crystallographic structure of the human C3 and represented by boxes with different colors (48). The chicken and fish domains were predicted based on a multiple sequence alignment and conserved aa position. The consensus aa sequence of the thioester motif involved in attachment of complement to the target cells (17) is indicated. The size (aa) of the sequences represented is indicated as well as the two four arginine residue processing sites at the end of the β- and α-chain. Full-length sequences include β-, α- and γ-chains. The signal peptide (SP) was predicted for some full-length sequences. MG- macroglobulin domain (1–8); LNK- link domain; ANATO- anaphylatoxin domain; α-NT- N-terminal region of the cleaved α-chain; CUB- complement C1r/C1s, Uegf, Bmp1 domain; TED- thioester-containing domain; SAR- short anchor region; C345C- carboxy-terminal domain.

Supplementary Figure 5 Schematic representation of the predicted structural domains of C5 proteins (full-length and incomplete) from selected fish (spotted gar; three teleost’s the stickleback, sea bass and zebrafish; coelacanth; whale shark). The human and chicken sequences were included for comparisons. Twelve conserved protein domains were predicted and are represented by boxes with different colors. The thioester-containing domain is absent from C5. The size (aa) of the sequences represented is indicated as well as the four arginine residue processing sites at the end of the β-chain. Full-length sequences include both β- and α-chains. The signal peptide (SP) was predicted for some full-length sequences. MG- macroglobulin domain (1-8); LNK- link domain; ANATO- anaphylatoxin domain; α-NT- N-terminal region of the cleaved α-chain; CUB- complement C1r/C1s, Uegf, Bmp1 domain; TED- thioester-containing domain; SAR- short anchor region; C345C- carboxy-terminal domain.

Supplementary Figure 6 Multiple sequence alignment of the gilthead sea bream deduced C3 isoforms with the human C3. The 13 identified protein domains were annotated according to the crystal structure of human C3 (48) and homology modelling of the predicted structure of Antarctic teleost C3 (32). The two C3 chains β- (residues 1–645 aa) and α- (residues 650–1641) are indicated. SP- signal peptide, MG- macroglobulin domain (MG1-MG8); LNK-link domain; ANATO- anaphylatoxin domain; α-NT- N-terminal region of the cleaved α-chain, CUB- complement C1r/C1s, Uegf, Bmp1 domain; TED- thioester-containing domain; SAR- short anchor region; C345C- carboxy-terminal domain. The cysteine residues are highlighted in yellow and the small, colored triangles of the same color represent a predicted disulphide bond in human C3.1 available from Uniprot. N-glycosylation sites (N-x-T/S, where x represents any amino acid) are highlighted in green.

Supplementary Data Nototheniid complement and Cfh deduced protein sequences used in the construction of the phylogenetic trees.
