# Supplementary material for: Specific Evolution and Gene Family Expansion of Complement 3 and Regulatory Factor H in Fish
Source: Front Immunol. 2020 Dec 14;11:568631. doi: 10.3389/fimmu.2020.568631 (PMC7768046; doi:10.3389/fimmu.2020.568631)
Supplement: Supplementary Figure 1 — Phylogenetic trees of the fish C1, C2, C6-C9 genes. Accession numbers of the sequences are available in Supplementary Table 1 . The trees were built with the BI method and posterior probability values are shown. [file DataSheet_2.zip › Supplementary Figure 1.PDF]

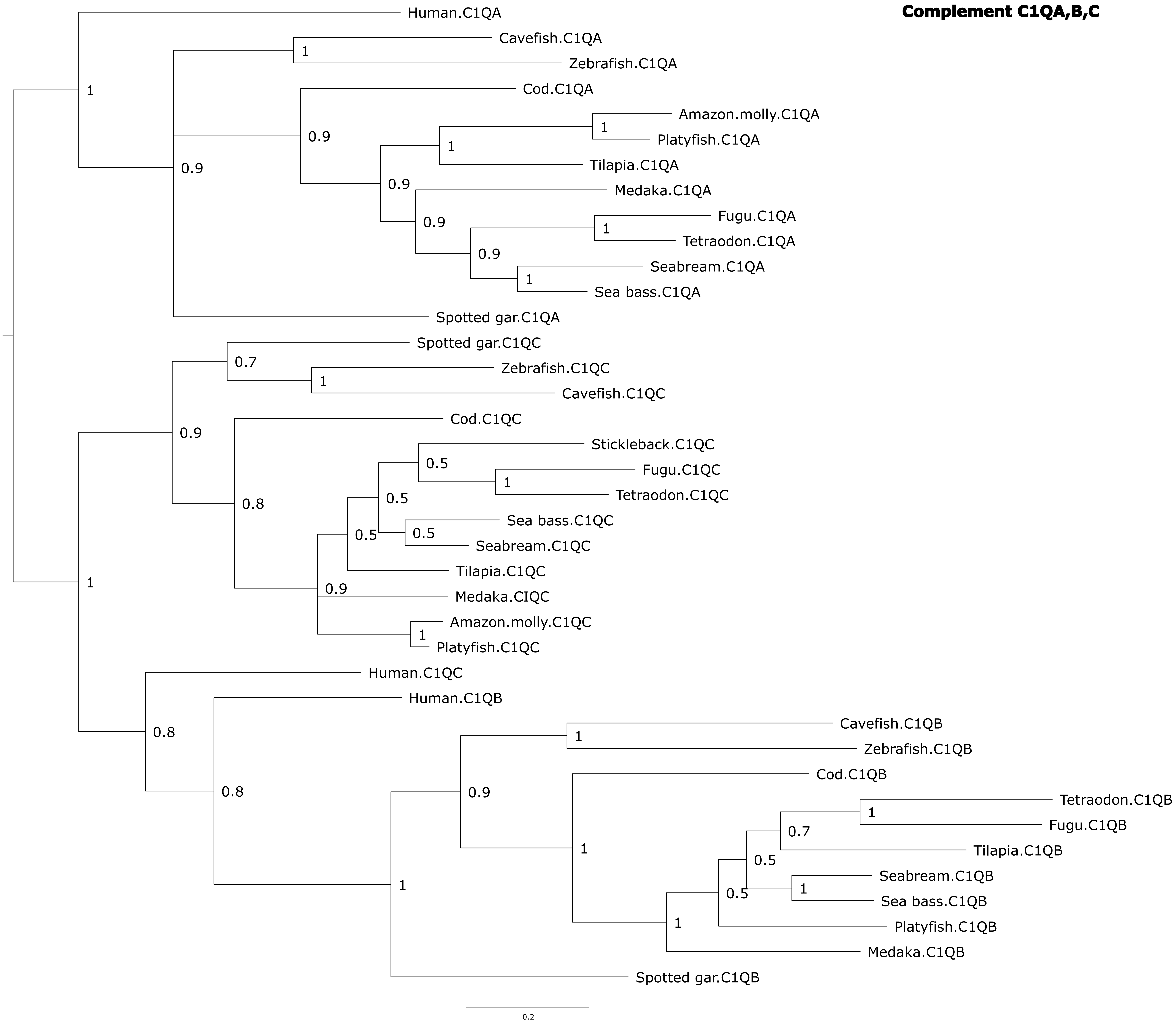
Complement C1QA,B,C
Human.C1QA
Cavefish.C1QA
Zebrafish.C1QA
Cod.C1QA
Amazon.molly.C1QA
Platyfish.C1QA
Tilapia.C1QA
Medaka.C1QA
Fugu.C1QA
Tetraodon.C1QA
Seabream.C1QA
Sea bass.C1QA
Spotted gar.C1QA
Spotted gar.C1QC
Zebrafish.C1QC
Cavefish.C1QC
Cod.C1QC
Stickleback.C1QC
Fugu.C1QC
Tetraodon.C1QC
Sea bass.C1QC
Seabream.C1QC
Tilapia.C1QC
Medaka.CIQC
Amazon.molly.C1QC
Platyfish.C1QC
Human.C1QC
Human.C1QB
Cavefish.C1QB
Zebrafish.C1QB
Cod.C1QB
Tetraodon.C1QB
Fugu.C1QB
Tilapia.C1QB
Seabream.C1QB
Sea bass.C1QB
Platyfish.C1QB
Medaka.C1QB
Spotted gar.C1QB
0.2

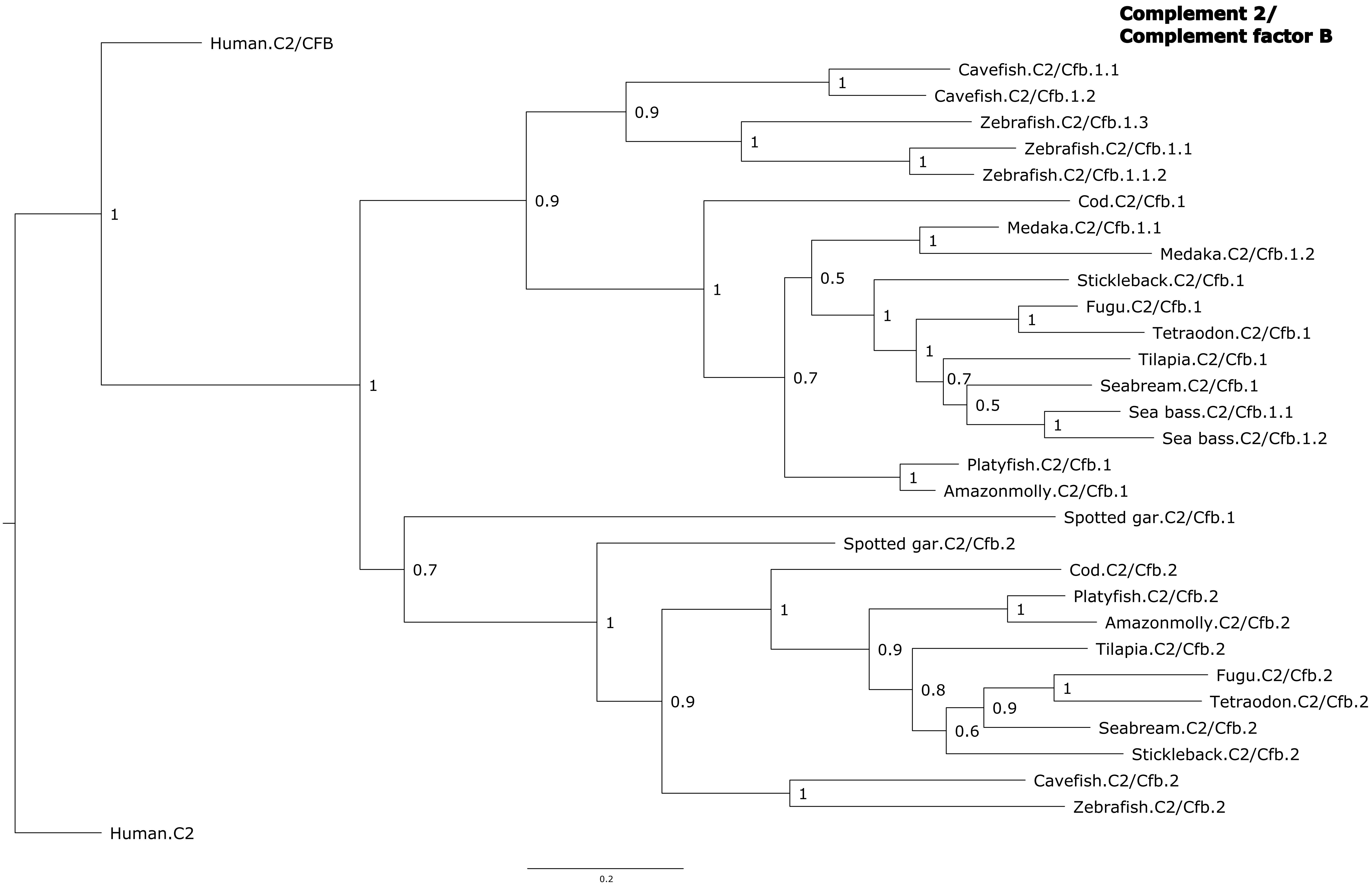

Complement 2/
Complement factor B
Human.C2/CFB
Cavefish.C2/Cfb.1.1
Cavefish.C2/Cfb.1.2
Zebrafish.C2/Cfb.1.3
Zebrafish.C2/Cfb.1.1
Zebrafish.C2/Cfb.1.1.2
Cod.C2/Cfb.1
Medaka.C2/Cfb.1.1
Medaka.C2/Cfb.1.2
Stickleback.C2/Cfb.1
Fugu.C2/Cfb.1
Tetraodon.C2/Cfb.1
Tilapia.C2/Cfb.1
Seabream.C2/Cfb.1
Sea bass.C2/Cfb.1.1
Sea bass.C2/Cfb.1.2
Platyfish.C2/Cfb.1
Amazonmolly.C2/Cfb.1
Spotted gar.C2/Cfb.1
Spotted gar.C2/Cfb.2
Cod.C2/Cfb.2
Platyfish.C2/Cfb.2
Amazonmolly.C2/Cfb.2
Tilapia.C2/Cfb.2
Fugu.C2/Cfb.2
Tetraodon.C2/Cfb.2
Seabream.C2/Cfb.2
Stickleback.C2/Cfb.2
Cavefish.C2/Cfb.2
Zebrafish.C2/Cfb.2
Human.C2
0.2

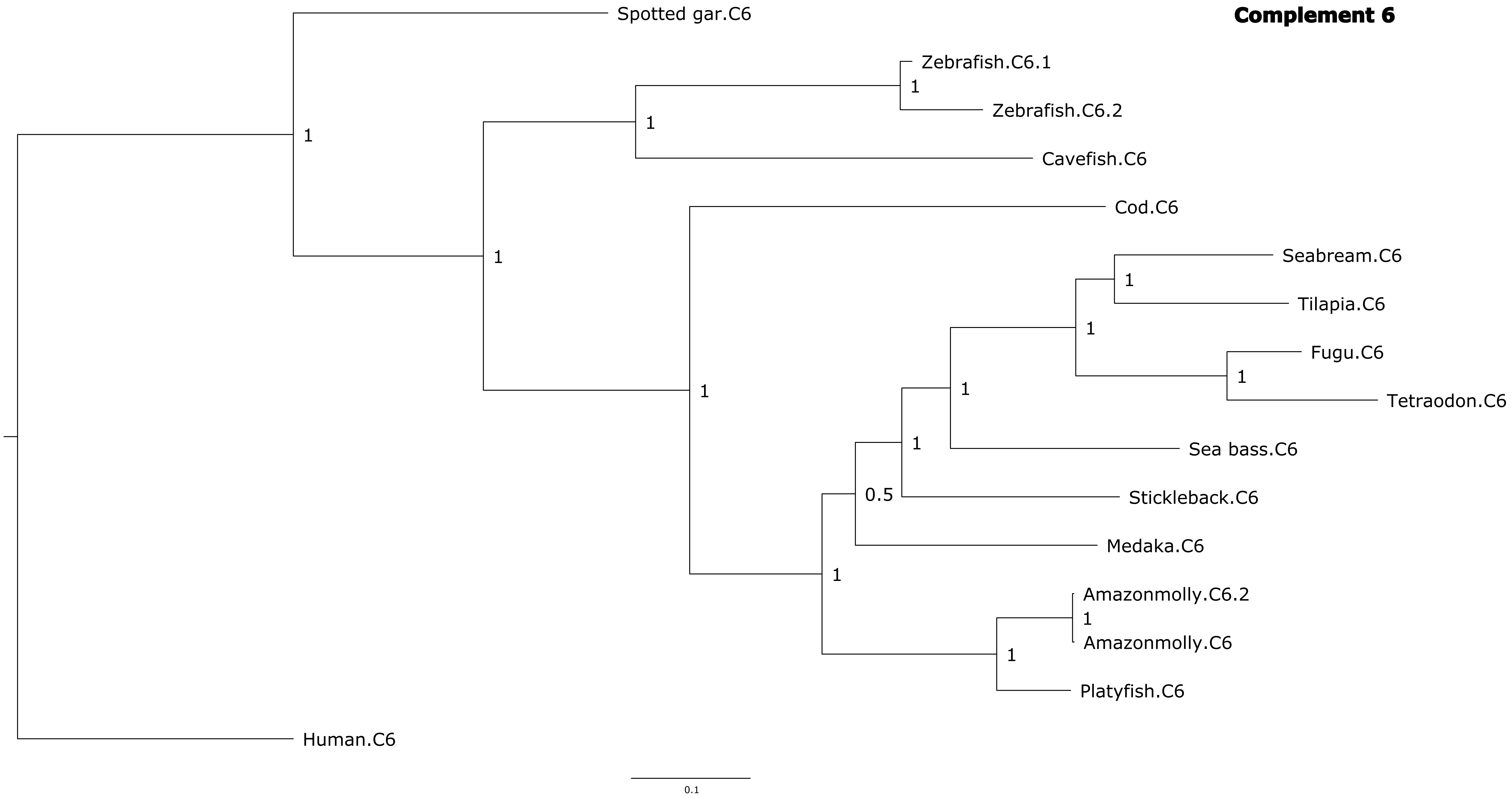
Complement 6
Spotted gar.C6
Zebrafish.C6.1
1
Zebrafish.C6.2
1
1
Cavefish.C6
Cod.C6
1
Seabream.C6
1
Tilapia.C6
1
Fugu.C6
1
Tetraodon.C6
1
1
1
Sea bass.C6
0.5
Stickleback.C6
Medaka.C6
1
Amazonmolly.C6.2
1
Amazonmolly.C6
1
Platyfish.C6
Human.C6
0.1

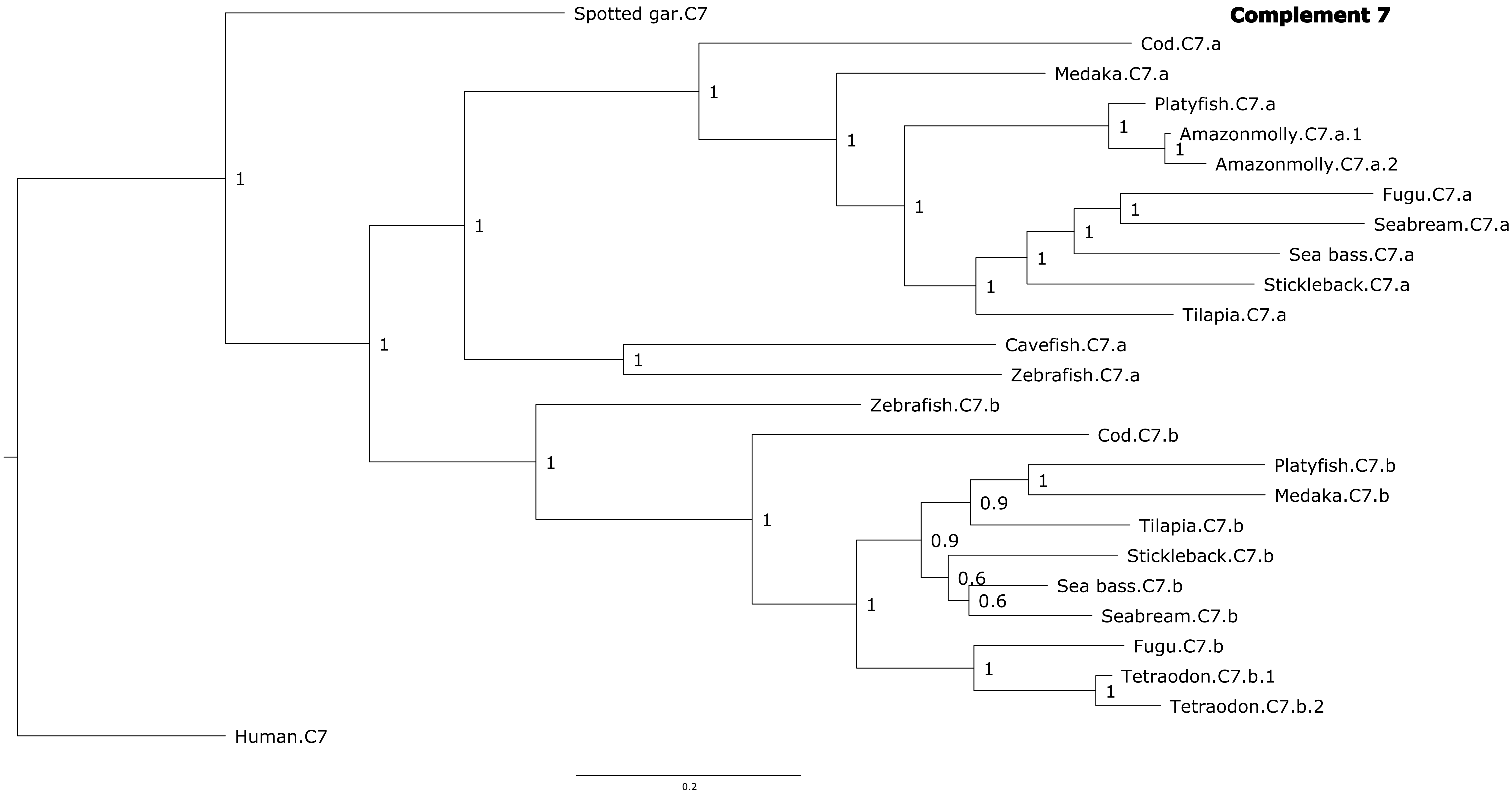
Complement 7
Spotted gar.C7
Cod.C7.a
Medaka.C7.a
Platyfish.C7.a
Amazonmolly.C7.a.1
Amazonmolly.C7.a.2
Fugu.C7.a
Seabream.C7.a
Sea bass.C7.a
Stickleback.C7.a
Tilapia.C7.a
Cavefish.C7.a
Zebrafish.C7.a
Zebrafish.C7.b
Cod.C7.b
Platyfish.C7.b
Medaka.C7.b
Tilapia.C7.b
Stickleback.C7.b
Sea bass.C7.b
Seabream.C7.b
Fugu.C7.b
Tetraodon.C7.b.1
Tetraodon.C7.b.2
Human.C7
0.2

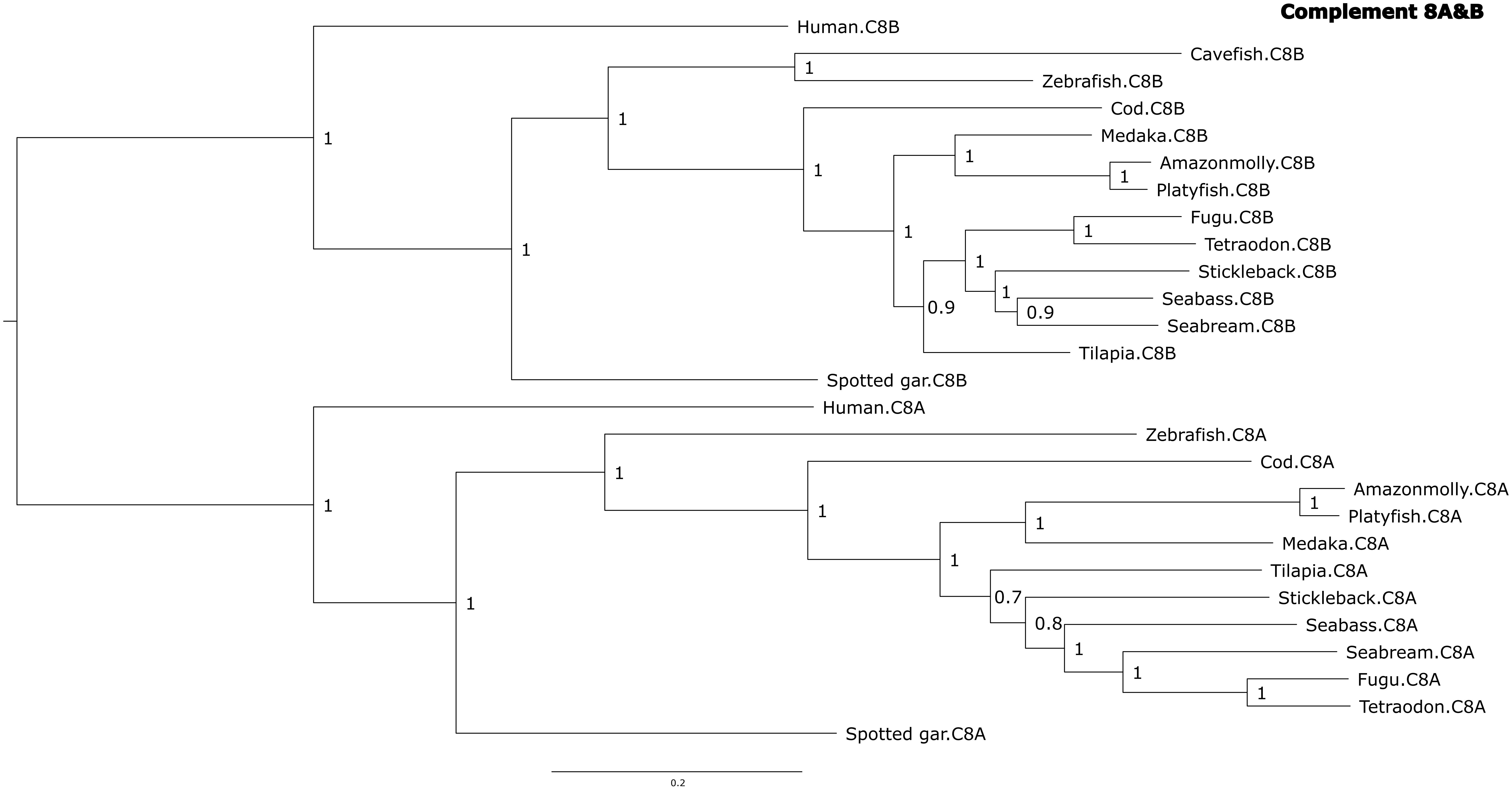
Complement 8A&B
Human.C8B
Cavefish.C8B
Zebrafish.C8B
Cod.C8B
Medaka.C8B
Amazonmolly.C8B
Platyfish.C8B
Fugu.C8B
Tetraodon.C8B
Stickleback.C8B
Seabass.C8B
Seabream.C8B
Tilapia.C8B
Spotted gar.C8B
Human.C8A
Zebrafish.C8A
Cod.C8A
Amazonmolly.C8A
Platyfish.C8A
Medaka.C8A
Tilapia.C8A
Stickleback.C8A
Seabass.C8A
Seabream.C8A
Fugu.C8A
Tetraodon.C8A
Spotted gar.C8A
0.9
0.7
0.8
0.2

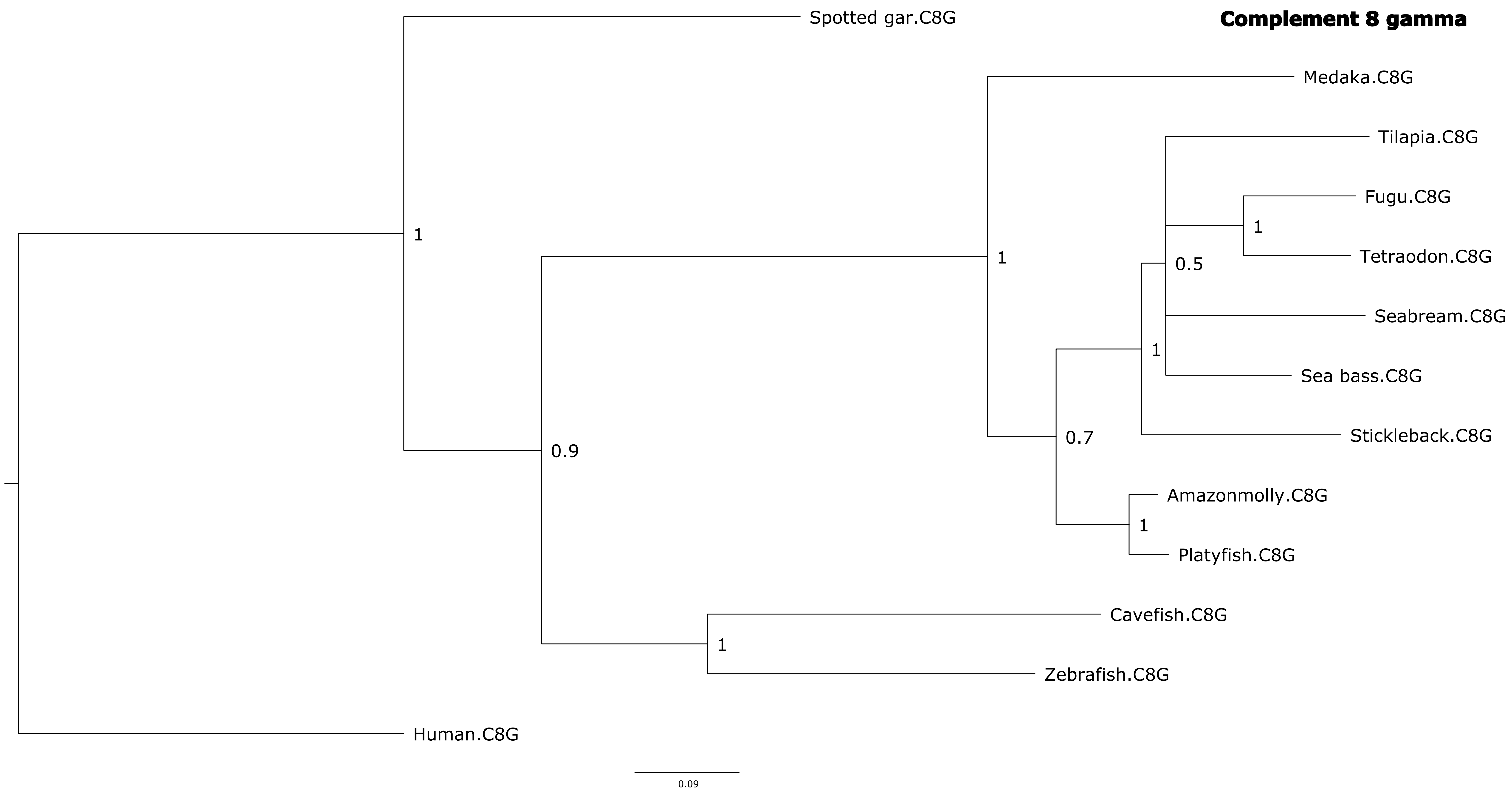

Complement 8 gamma
Spotted gar.C8G
Medaka.C8G
Tilapia.C8G
Fugu.C8G
1
Tetraodon.C8G
0.5
Seabream.C8G
1
Sea bass.C8G
Stickleback.C8G
0.7
Amazonmolly.C8G
1
Platyfish.C8G
1
1
0.9
Cavefish.C8G
1
Zebrafish.C8G
Human.C8G
0.09

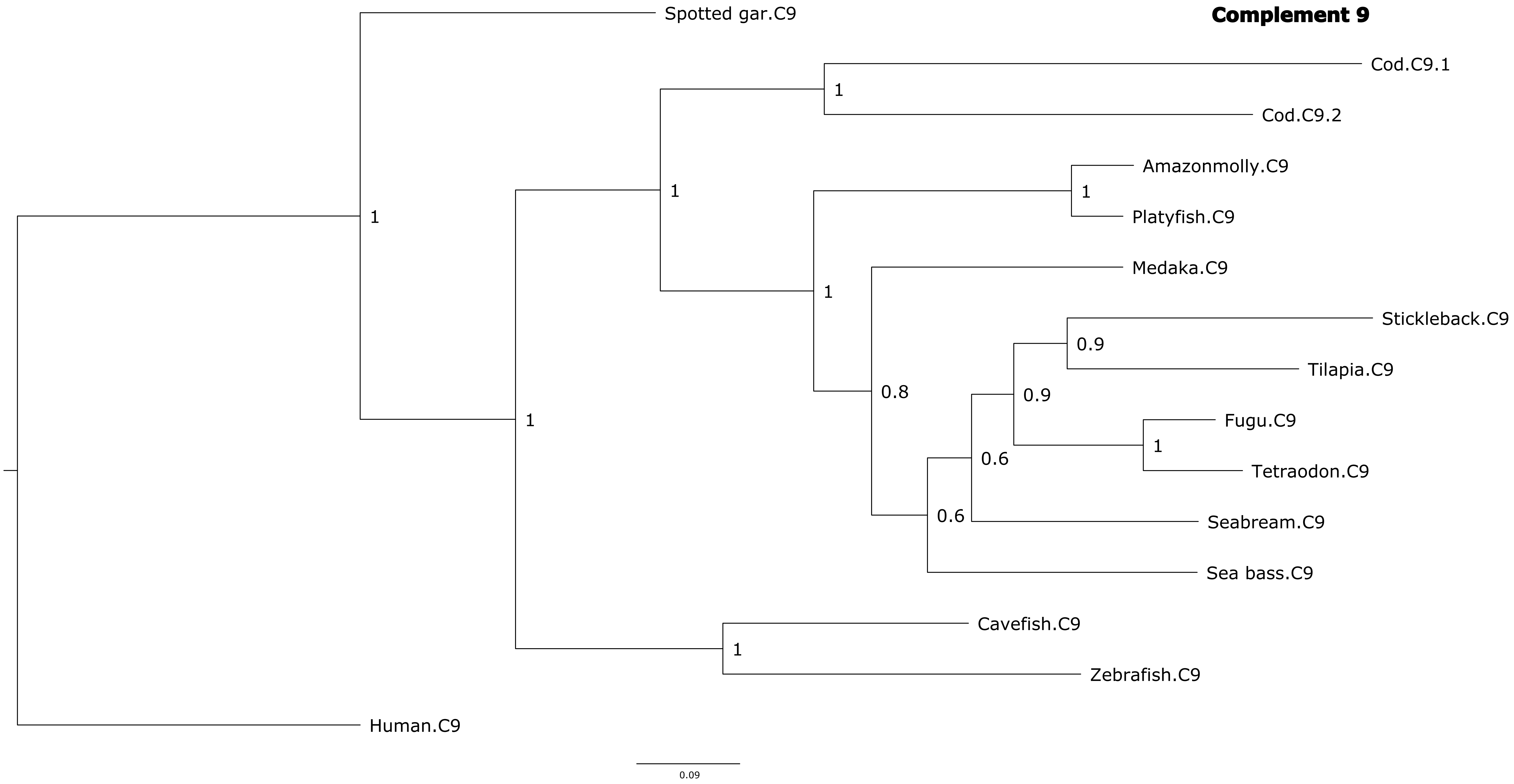

Complement 9
Spotted gar.C9
Cod.C9.1
Cod.C9.2
Amazonmolly.C9
Platyfish.C9
Medaka.C9
Stickleback.C9
Tilapia.C9
Fugu.C9
Tetraodon.C9
Seabream.C9
Sea bass.C9
Cavefish.C9
Zebrafish.C9
Human.C9
1
1
1
1
1
1
1
1
1
0.9
0.9
0.8
0.6
0.6
0.09
