# Supplementary material for: Specific Evolution and Gene Family Expansion of Complement 3 and Regulatory Factor H in Fish
Source: Front Immunol. 2020 Dec 14;11:568631. doi: 10.3389/fimmu.2020.568631 (PMC7768046; doi:10.3389/fimmu.2020.568631)
Supplement: Supplementary Figure 1 — Phylogenetic trees of the fish C1, C2, C6-C9 genes. Accession numbers of the sequences are available in Supplementary Table 1 . The trees were built with the BI method and posterior probability values are shown. [file DataSheet_2.zip › Supplementary Figure 3.PDF]

```
Human.C3.1        : ------------------------------------------------------------------------------------------ :   -
Chicken.C3.1.1    : MSPCCPLPPPAVLQCGPPPAAFPEMRKVPNIRAAPPSLQSRGRVGGGGGGGDTHTLDGDPTLISTPMGVPHPTAPISAPQVDPGPRYPMGD :  91
Coelacanth.C3.1.1 : ------------------------------------------------------------------------------------------ :   -
Coelacanth.C3.1.2 : ------------------------------------------------------------------------------------------ :   -
Spotted gar.C3.1.1: ------------------------------------------------------------------------------------------ :   -
Spotted gar.C3.2  : ------------------------------------------------------------------------------------------ :   -
Sea bass.C3.1.1   : ------------------------------------------------------------------------------------------ :   -
Sea bass.C3.1.2   : ------------------------------------------------------------------------------------------ :   -
Sea bass.C3.1.3   : ------------------------------------------------------------------------------------------ :   -
Sea bass.C3.2     : ------------------------------------------------------------------------------------------ :   -
Stickleback.C3.1.1: ------------------------------------------------------------------------------------------ :   -
Stickleback.C3.1.2: ------------------------------------------------------------------------------------------ :   -
Stickleback.C3.1.3: ------------------------------------------------------------------------------------------ :   -
Stickleback.C3.2.1: -----------------------------------------------------------------------------------------V :   1
Stickleback.C3.2.2: ------------------------------------------------------------------------------------------ :   -
whale shark.C3.1.2: ---------------------------------------------------------------------------------MFVPVKQ :   7
Lamprey.C3x1      : ------------------------------------------------------------------------------------------ :   -
Lamprey.C3x2      : ------------------------------------------------------------------------------------------ :   -
```

SP

β-chain

MG1

```
Human.C3.1        : MGPTSGPSLLLLLLTHLP-LALGSP-----------------------------------------------MYSIITPNIIRLESEET :  41
Chicken.C3.1.1    : PTVRALLWVTLGDVVVPTGLGVGYPLTNTAAIKDPPPPTPNPRPPRPHPRSAMGCCCCPSCSAFCCSMRSPPAQMVTVVTEAVIRLDIDEK : 182
Coelacanth.C3.1.1 : ------------------------------------------------------------------------------------------ :   -
Coelacanth.C3.1.2 : --MEAAGLLLSVLLLFFPAPSHSKS-----------------------------------------------FAVITAPNVIRVESEEM :  40
Spotted gar.C3.1.1: SILMFNLTLSNISRFYLYIFSFI-------------------------------------------------SYVITAPNVIRVSSEEN :  40
Spotted gar.C3.2  : TDRMAGVWLSLVLLASFFGLSLGEP-----------------------------------------------LYIIMAPNIFRLDSPEN :  42
Sea bass.C3.1.1   : ------------------------------------------------------------------------------------------ :   -
Sea bass.C3.1.2   : MSRTLLWLVASLAFASLTSLADGAP-----------------------------------------------LQVVSAPNLIRVGTPEN :  42
Sea bass.C3.1.3   : MSRTLLWLVASLAFASLTSLADGAP-----------------------------------------------LQVVSAPNLIRVGTPEN :  42
Sea bass.C3.2     : ---MGRWTLHSILFLVLICHANGQFDNIRRPPWRERFTPIGRYWDLHEIPERKTVSTAP-------------RFTILAPDLIRTDSQEN :  73
Stickleback.C3.1.1: MSRTMWWLVASVASTLLTCLANGAP-----------------------------------------------LKVVSAPNLIRVGTAEQ :  42
Stickleback.C3.1.2: MSRTMWWLVASVASTLLTCLANGAP-----------------------------------------------LKVVSAPNLIRVGTAEQ :  42
Stickleback.C3.1.3: ------------------------------------------------------------------------------------------ :   -
Stickleback.C3.2.1: GGAAEVNLVSLFSAIVSTSLAVC-------------------------------------------------TFTILAPDLIRTDSQEN :  41
Stickleback.C3.2.2: LVPIVGPSQLMLVLSTADKNQ--GP-----------------------------------------------RFTILAPDLIRTDSQEN :  40
whale shark.C3.1.2: TAIMRKTFLLLGLLAFLPAQFHCMQ-----------------------------------------------LYVITAPNVIRVEDEQN :  49
Lamprey.C3x1      : --MAAHALGLLLLLMSVGTSVTQDP-----------------------------------------------MVILSVESVILIGSDVN :  40
Lamprey.C3x2      : --MAIHALGLLLLLMYVGTSVTQDP-----------------------------------------------MVLLSVESVILAGSDVN :  40
```

MG1

```
Human.C3.1        : YVIEAHDAQ-GDVPVTVTVHDFPGKKLVLSSEKT--VLTPATNHMGNVTFTIPANREFKSEKGRNKFVTVQATFGTQVVEKVV----LVSL : 125
Chicken.C3.1.1    : YVLEAPGLS-APTEANLVQDFPQRRKVLFQVRK--QLNPAEGMMAIATVKVPV--KLLPPVVGKHFVSVVARVGQVTLEKVL----LVSL : 264
Coelacanth.C3.1.1 : ------------------------------------------------------------------------------------------ :   -
Coelacanth.C3.1.2 : VVVEVHNYD-AATEVTIIVQNYPQKQRSLFHVQR--TIDSSTGYQLSEKIKIPID-QEAKGSKDKQFVFIQVTSSWFTLEKVI----LLSF : 123
Spotted gar.C3.1.1: IMVEAHDYSGGSLKVDILVLDFQRKRQQLHKSHV--TINHAGNQFQALHKIKIPDN-SFKKESSSNQYVIIQARFEGEVLEKIV----LVSF : 124
Spotted gar.C3.2  : VFLEAQGTS-DTIHVTIKALMFPGR-QSLSETTV--ELNSANKHQALKALKIPSK-NLQRD--TNQMAYLSADFKGNIVESVV----LLSF : 122
Sea bass.C3.1.1   : ------------------------------------------------------------------------------------------ :   -
Sea bass.C3.1.2   : IFVECQDCTGGDIRVDIKVMNHPTRTKELATTSV--TITTANNFQEFGQLKIPAG-DFSKDPSIKQYVYIQARFPDRLLEKVV----LVSF : 126
Sea bass.C3.1.3   : IFVECQDCTGVDIRVDIKVMNHPTRTKELATTSV--TITTANNFQDFGQLMIPAG-DFSKDPSIKQYVYIQARFPDRLLEKVV----LVSF : 126
Sea bass.C3.2     : IYIQADGVS-NPITVSISIKDF-GRTTTLLEDSV--TINQENGFHVLKTIQLPSD-RLNREEKTNKFVYILKVTFGGFHSEERVL---MVSF : 156
Stickleback.C3.1.1: IFVECQDCTGGDINVIISVWSHPTKDEELANTAV--TINAGNHFQQLGRITIPAR-YFSREASTKHYVYIKAQFPDRVLEKVV----MLSF : 126
Stickleback.C3.1.2: IFVECQDCTGGDINVIISVLSHPTKEEELANTAV--TINAGNHFQQLGRITIPAG-DFSREASTKHYVYIKAEFPDRVLEKVV----MLSF : 126
Stickleback.C3.1.3: -------------------------------------TTYLALH-IKIPAG-DFSREASTKHYVYIKAQFPDRVLEKVV----MLSF :  44
Stickleback.C3.2.1: NYIQANGVS-TPVSVTITVMDFRSRAMLLRHAQCGQRVSCSQGDPDLIQ-QLPSD-GLNPEEKENKFVYILKVDFGGFHSEERVL---MVSF : 126
Stickleback.C3.2.2: IYIQANGVS-TPVSVTITVMDFRSRAMLLLDSI--TINVDNGFHALKAIQLPSD-GLNPEEKENKFVYILKVDFGGFHSEERVL---MVSF : 123
whale shark.C3.1.2: VVVEGHGIN-EDLKVDIR-LSFFTKSDIIFRTEG--IISSSNNYQISVPITIPRE-RIRIESTSHQYVVIEAQSSVFILVQTI----LVSY : 131
Lamprey.C3x1      : VLVDARGLT-EDVRVVVRAEEFLTKKQLATQTITLTQLDPAIATLKLG-FDIENP-DKTNSPSTKHHVRIVAKVESKSFNKEVTAHALLSY : 128
Lamprey.C3x2      : VLVDTRGLT-EDGSVMVRVEEFLTKKQLATQTIPLTQLDPAIATLKLG-FDIENP-DKTNSQSTKHHVRIVAKVESKSFNKEIMAHALLSF : 128
```

MG2

```
Human.C3.1        : QSGYLFIQTDKTIYTPGSTVLYRIFTVNHKLLPVGR--------TVMVNIENPEGIPVKQDSLSSQNQL-GVLPLS-WDIPELV------- : 199
Chicken.C3.1.1    : QSGHIFLQTDKPIYTPGSTVLCRLFALSHFMQPLLK--------TVIVEVKTPDNVIIKQVPVSSPMRN-GIFSIN-HNLPEVV------- : 338
Coelacanth.C3.1.1 : ------------------------------------------------------------------------------------------ :   -
Coelacanth.C3.1.2 : HTGYIFIQTDKPLYTPDSTVLYRLFTVTHELKPRKK--------SVIVEFQTPEGIVVKRNSVSSTDAE-LFYSGI-YEIPMIV------- : 197
Spotted gar.C3.1.1: LSGYIFVQTDKSIYTPTDTVLYRVFALTSRMIPCQT--------SVSIEIMNPDGIVVQRDVFFPSN---GIISKS-YSLPEIV------- : 196
Spotted gar.C3.2  : QSGYVFVQTDKPIYNPTDTVMYRAFVTSTAATAIDS--------SVTIEFKNPQGIVINSVLHGNVK-N-GILTTN-YKLPEVV------- : 195
Sea bass.C3.1.1   : ------------------------------------------------------------------------------------------ :   -
Sea bass.C3.1.2   : QSGYIFIQTDKTLYTPNSKVYYRMFALSPRMEPVERDPETQADTYIAIDIVTPEDIIIPLHPVSLKS---GIHSGR-YQLGAIV------- : 206
Sea bass.C3.1.3   : QSGYIFIQTDKTLYTPNSKVYYRMFALSPRMEPVERDPETQADASIAIDIVTPEDIIIPLDPVSLKS---GIHSGH-YQLGEIV------- : 206
Sea bass.C3.2     : HSGYIFIQTDKPIYNPGDTVRFRAFASSTSFKSFDS--------SVTIDIQNPDGVVVKQISRTSAAD--GIYTDT-LAISEFV------- : 229
Stickleback.C3.1.1: QSGYIFIQTDKTLYTPKDKIYYRMFAVTPRMEPVGADAQNQPEVSISIEIVTPENIVIPLDPVVLKS---GIHSGD-YQLPEIA------- : 206
Stickleback.C3.1.2: QSGYIFIQTDKTLYTPKDKILYRMFAVTPRMEPVETDAQNQPEVSIYIEIVTPEGVIVPRDPVGLIS---GIHSGF-YQLPEIA------- : 206
Stickleback.C3.1.3: QSGYIFIQTDKTLYTPKDKILYRMFAVTPRMEPVGADAQNQPQVSISIEIVTPEGVIVPRDPVSLIS---GIHSGD-YQLPEIARLVLYLF : 131
Stickleback.C3.2.1: HAGYIFIQTDKPIYNPGDTVRFRAFASSLSFKAFDS--------SVTIDVTNPDGVVVKQVSRVRPAD--GVLDDT-LLISEIV------- : 199
Stickleback.C3.2.2: HAGYIFIQTDKPIYNPGDTVRFRAFASSLSFKAFDS--------SVTIDVTNPDGVVVKQVSRVRPAD--GVLDDT-LPISEIV------- : 196
whale shark.C3.1.2: QLGHIFIQTDKPIYTPTQTVRYRLFSVNNEMKPTNI--------DAFVDFVNPQGVIVKRVEERSRNPS-SIIGST-FPIPKIA------- : 205
Lamprey.C3x1      : RSGHVVVQTDKPIYTPDEKVKYRMFPMNQKMSPIPGSQ------SVTVDIVNADGVIVERQIKTIKSTDEGIVDGTSFTIPAIS------- : 206
Lamprey.C3x2      : RSGHVVVQTDKPIYTPDEKVKYRMFPMNQKMSPILGSQ------SVTVDIVNADGVIVERQIKTIKSTDEGIVDGTSFTIPAIA------- : 206
```

MG2 MG3

```
Human.C3.1        : ---------------------NMGQVKIIRAYYENSPQQVFSTEFEVKEYVLPSFEVIVEPTEKFYYIYNEKGLEVTITARFLYGKKVEGTA : 269
Chicken.C3.1.1    : ---------------------SLGTVTITAKFEDSQDQVFSTQFEVKEYVLPSFEVTLDPQEKFLYIDRQEDFRVTITARYLYGKNIQGTA : 408
Coelacanth.C3.1.1 : ------------------------------------------------------------------------------------------ :   -
Coelacanth.C3.1.2 : ---------------------SLGVVNITARYEDASQEPFSTQFEIKEYVLPSFEVKIVPAQKPFRIDTKK-LEVAITCRYLYGKPISGSA : 266
Spotted gar.C3.1.1: ---------------------PFGTVKIVTKHEKTPQKEFTTDFEVKEYVLPSFEVKIEPQQPFFYIDGKE-LSVKITARFLYGSNVVGSA : 265
Spotted gar.C3.2  : ---------------------NEGIVQITAQLDT--QSTFITEFQVKEHVLPSFEVIINPRKKFHIDDKS-LVVEISASYVYGEEVKGSA : 262
Sea bass.C3.1.1   : ----------------------------------------------------------------------------------------MA :   2
Sea bass.C3.1.2   : ---------------------SPGLVKVVAKFQSNPQQSYYAEFEVKEYVLPSFEVKLIPASSFFYVDSEE-LKVNIKATYLFGEEVNGMA : 275
Sea bass.C3.1.3   : ---------------------SPGLWKVVAKFQSNPQQSYYAEFEVKEYVLPSFEVKLIPVSSFFYVDSEE-LTVNIKATYLFGEEVNGMA : 275
Sea bass.C3.2     : ---------------------NEGTVTVTAKFDHWEQNTFTSKFEVKKYVLPAFNVTITPRKSFLNIDDSE-LVIEISASYLYGEPVQGTA : 298
Stickleback.C3.1.1: ---------------------SSGLWKVVTRFRSNPQLSYSAEFEVKEYVLPSFEVKLKPTSSFFYVDGGE-FTVNIEAKYLFDEWVDGTA : 275
Stickleback.C3.1.2: ---------------------SPGLWKVVTRFRSNPQQSYSAEFEVKEYVLPSFEVKLKPTSSFFYVDSEQ-FTVNIEAKYLFDEWVDGTA : 275
Stickleback.C3.1.3: IFHISFFDIVLLSGVLLIYFFSFRLWKVVTRFHSNPHQSYSAEFEVKEYVLPSFEVKLKPTSSFFYVDSEE-FTVNIEAKYLFDEWVDGTA : 221
Stickleback.C3.2.1: ---------------------NEGKVTITAKFDHRQQNTFTSEFEVKKYVLPAFNVTITPKKSFLNLEDSE-LQVEISARYLYGEPVQGTA : 268
Stickleback.C3.2.2: ---------------------NEGKVTITAKFDHRQQNTFTSEFEVKKYVLPAFNVTITPKKSFLNLEDSE-LQVEISARYLYGEPVQGTA : 265
whale shark.C3.1.2: ---------------------NIGIVKITVSYRETSHVNYTTAFEVKEYVLPSFEVTLESKEKFFYVDDTK-LDVSITARFTYGKPVEGRA : 274
Lamprey.C3x1      : ---------------------KHGTVKIFARMSGAPNTNSSAEFDVREYILPTFEVKINPKQRVFHINDEE-FVVDITANYFNQEPVSGTA : 275
Lamprey.C3x2      : ---------------------KHGTVKIFARMSGAPNVNSSAEFDVQEYILPTFEVKVNPKQRVFHINDEE-FVVDITANYFNQELVSGTA : 275
```

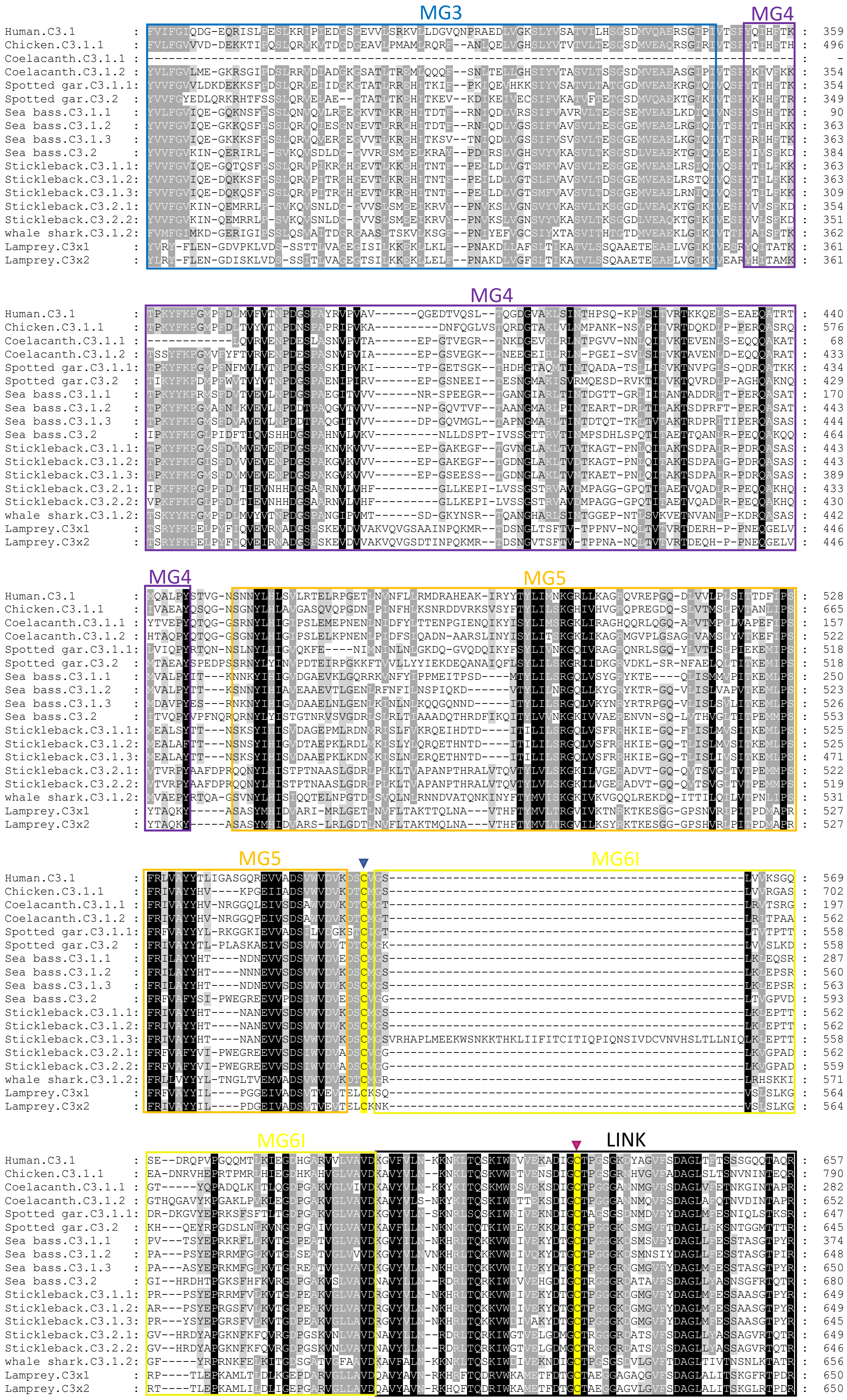

MG3
MG4
MG4
MG4
MG5
MG5
MG6I
MG6I
LINK

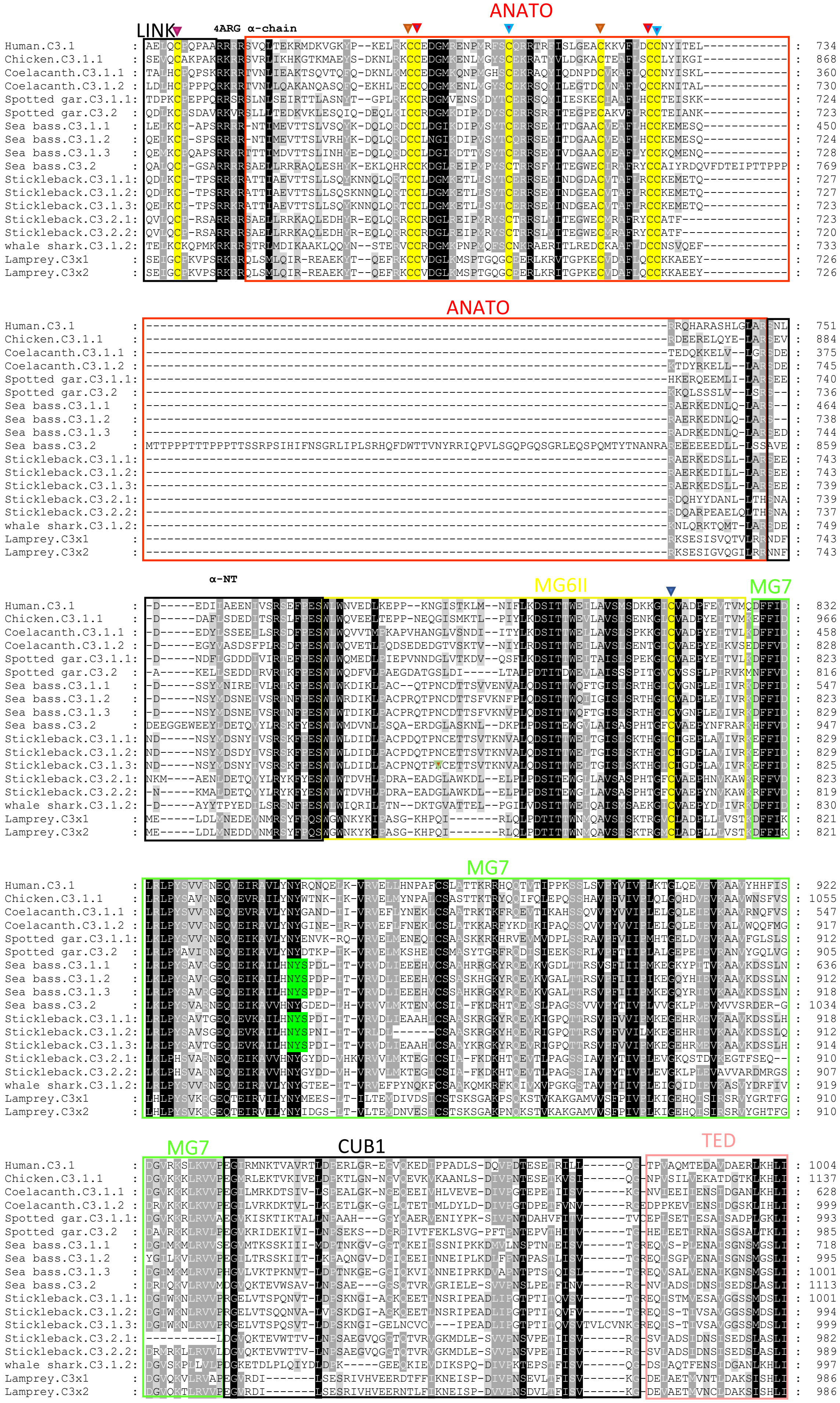
LINK
4ARG
α-chain
ANATO
ANATO
α-NT
MG6II
MG7
MG7
MG7
CUB1
TED

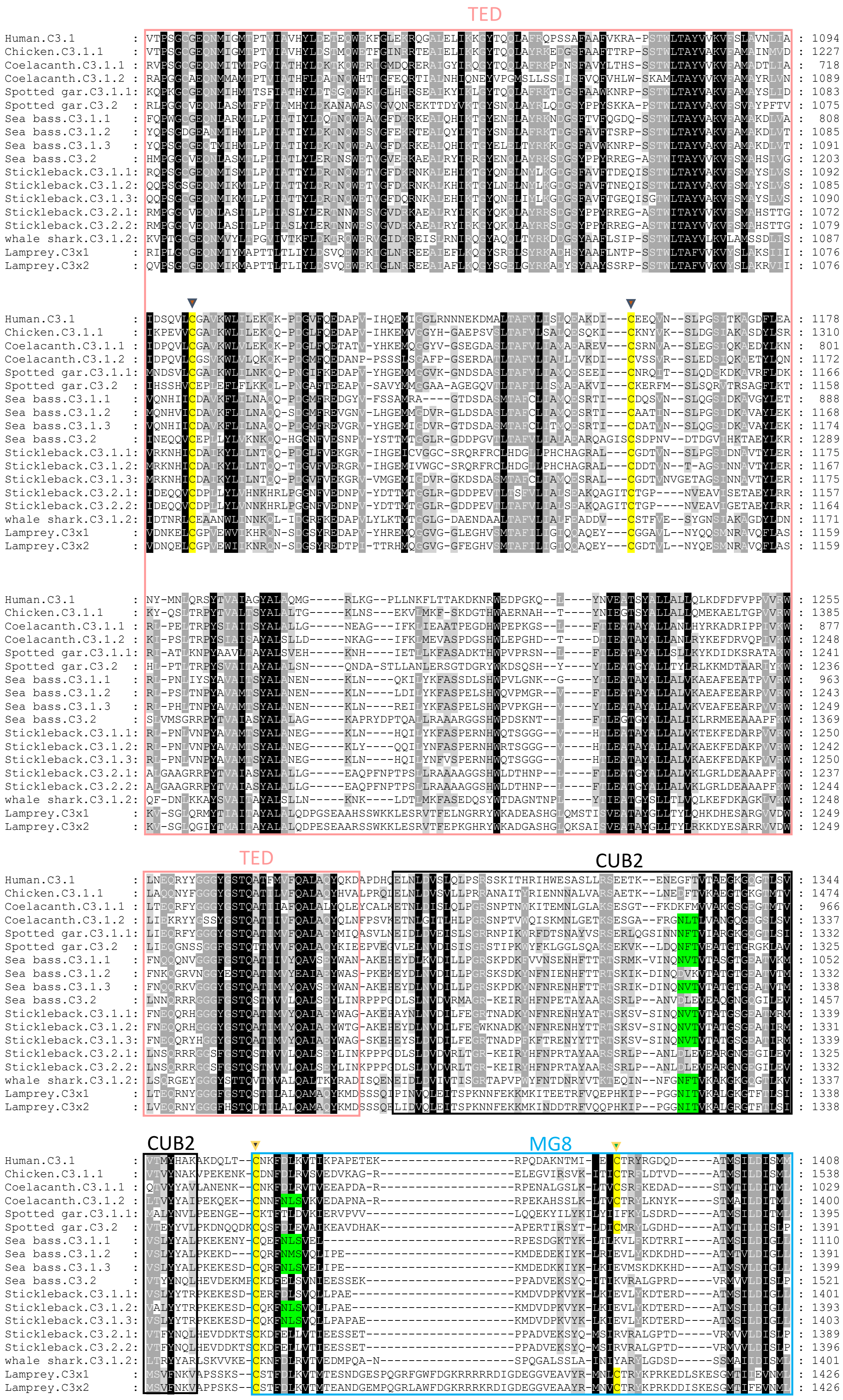
TED
TED
CUB2
CUB2
MG8
Human.C3.1
Chicken.C3.1.1
Coelacanth.C3.1.1
Coelacanth.C3.1.2
Spotted gar.C3.1.1
Spotted gar.C3.2
Sea bass.C3.1.1
Sea bass.C3.1.2
Sea bass.C3.1.3
Sea bass.C3.2
Stickleback.C3.1.1
Stickleback.C3.1.2
Stickleback.C3.1.3
Stickleback.C3.2.1
Stickleback.C3.2.2
whale shark.C3.1.2
Lamprey.C3x1
Lamprey.C3x2

MG8

```
Human.C3.1          : TGFAPDTDDL--KQLANGVDRYISKYELDKAFSDRNTLIIYLDKVSHSEDDCLAFKVHQYFNVELIQPGAVKVYAYYNL--EESCTRFYHP : 1495
Chicken.C3.1.1      : TGFSPDVQDL--KSLSEGVERYISKFEIDHALSNRSNLIIYLDKVSHQVEECIAFRAHQHFQVGLIQPASVTVYSYYKI--DDRCTRFYHP : 1625
Coelacanth.C3.1.1   : TGYSPDIGDL--NRLQNGVDRYISKYEMDKVLSAKGSLIIYLDKVSSKEDDCIALKLHQMYKVGLIQPAAVTVYEYYNT--ENHCTRFYHP : 1116
Coelacanth.C3.1.2   : SGYSPDTGDL--NMLQNGVEQYIELHETDKALSDKGSLIIYLDKVSFEEDDCIAFKVHQMYKVGLIQPAAITVYEYYNK--ENRCTKFYHT : 1487
Spotted gar.C3.1.1: SGFIPDAGDL--KRLSNGVDQYIQKYEMDKALSDKGSLILYLDKVSHKIPDRIAFKVHKINEVGLMQPAAVTIYEYYANVPENHCVKFYHP : 1484
Spotted gar.C3.2    : TGCVPSLENLQNQKLQNRVDRYIDHFNLDKELSERGSLIIHLYKVLN-ETQCLSITLHQMFEVKLIQPSSVTIYGYYTN--DIRCTRFYHP : 1479
Sea bass.C3.1.1     : TGFTANTNDL--NLLSKGPARIIARYELDSALSEGSSVTIYLNKVSNVQPEEITFRIHQQMRVGVLQPAAVSVYEYYDP---TPCVKFYHP : 1196
Sea bass.C3.1.2     : TGFTVNTNDL--SLLSKGRARTISKYNMKTVQSERGSLIIYLDKVSHTRPEEIMFRMHQKLKVGVLQPAAVSVYEPNHQY-TTPCVKFYHP : 1479
Sea bass.C3.1.3     : TGFTVDTNDL--NLLSKGRARTIAKYEMDTVLSERGSLIIYLDKISHTQPEEITFRIHQQMKVGVLQPAAVSVYEYYD---QTPCVKFYHP : 1485
Sea bass.C3.2       : TGFTPENSDL--EMLSNSVDHYINNFQIVDNLSDRGSLIIHLFKVSHREPEILIFRLQQSFKVGLLQPSSVTVYEYYNP--DHRCSRTYSP : 1608
Stickleback.C3.1.1: TGFTADINDL--NRLSKGPARTIANYEMNTVLSDRGSLIIYLNKVSHTLPEEITFRVHQTMKAAALQPAAVSVYDYYD---EKRCVKFYHP : 1487
Stickleback.C3.1.2: TGFTADINDL--NQLSKGPARTIAKYEMNTVLSDRGSLIIYLNKVSHTLPEEITFRVHQTMKAAALQPAAVSVYDYYD---EKHCLKFYHP : 1479
Stickleback.C3.1.3: TGFTADINDL--NRLSKGPARTIANYEMNTVLSDRGSLIIYLNKVSHTLPEEITFRVHQTMKAAALQPAAVSVYDYYDA--EKRCVKFYHP : 1490
Stickleback.C3.2.1: TGFSPETSDL--EKLSNSVDRYINHFQVVDNLSDRGSLIIHLFKVSHKEPEVLIFRLQQRFRVGLLQPSSVTVYEYYNP--DHRCSHTYTP : 1476
Stickleback.C3.2.2: TGFSPETSDL--EKLSNSVDRYINHFQVVDNLSDRGSLIIHLFKVSHKEPEVLIFRLQQRFRVGLLQPSSVTVYEYYNP--DHRCSHTYTP : 1483
whale shark.C3.1.2: TGFSPDLDDL--NSLQNGVDRYISRYELDKSLSMHGSLIIYLNSVSHTEDTQVGFKIHQFYKVGLIQPASVKVYEYYNK--ENSCSKFYNV : 1488
Lamprey.C3x1        : TGFLPDKNDL--IQLKDSVDKYISYYEITDSV-----LIIYLDKVPSTENYCFAFKIKQMLRSDMIQPVTASVYDYYSP--QDKCTRFYNL : 1508
Lamprey.C3x2        : TGFIPDKNDL--IQLKNAVDKYISNYEITDSV-----LIIYLDTVPSTEDYCFAFKIKQMLKSDMMQPVTASVYDYYSP--QDKCTIFYNL : 1508
```

C345C

```
Human.C3.1          : EKEDGKLNKLCRD--ELCR-CAEENCFIQK--SDDKVTLEERLDKACEPG----VDYVYKTRLVKVQLSNDFDEYIMAIEQTIKSGSD-EV : 1576
Chicken.C3.1.1      : DKAGGQLRKICHG--EVCR-CAEENCFIRV-KKDNPITVNERIDLACKPG----VDYVYKVKVVATEETPSHDNYIMSILTVIKMGTD-EN : 1707
Coelacanth.C3.1.1   : TKESGLLSKICQA--EVCR-CAEEKCGALQ-KHGTQISIEERTDEACAVG----VDYVYKAKFVDLNETDSHDTYVMQILEVIKAGSDKGE : 1199
Coelacanth.C3.1.2   : SKESGSLNKICQG--EVCR-CAAEKCGALQ-KEDDTISAFDRSEKACEVG----VDYVYKVKFVNRTEYDSHDAYIMEIVETIKQGSDGKI : 1570
Spotted gar.C3.1.1: EKETGTLNKICHG--DVCK-CAEDNCYILK-QNNPDIDQNIRFEKACEAG----MDYVYKAKLIDSSLTAVYDQYTMLIEDVIKEGTD-EG : 1566
Spotted gar.C3.2    : VKKQAQLGRICRD--SVCR-CAEESCSALK-KKDEEITVDRRLIKACTA-----TNYVFKAKLIKRNPSSSYEEYIMEIEAVLKQGDE-LF : 1560
Sea bass.C3.1.1     : ERRAGQLLQLSRN--DVYRECAEENCSMQK---KGNIDNDDRTAKACESTETTKIDFVYKVELQKFEDGLSTDVYTMRVLKVIKEGTTDVG : 1282
Sea bass.C3.1.2     : ERRAGQLLRLCRN--DVCT-CAEENCSMQK---KNNISNDERTAKTCETQVNSKIDFVYKVRLQEFTDGLSTDIYTVRVMEVIKEGNDDVG : 1564
Sea bass.C3.1.3     : ERRAGELLQLCRN--DVCTVCAEENCSMQK---KGNIDNDQRTAKACESTETTKIDFVYKAELRKFEDGLSTDVYTMRVLKVIKEGTTDVG : 1571
Sea bass.C3.2       : REDKEGLTQICRD--NVCR-CTQGDCCVAK-TDDENFSNKERETFACKS-----LHHVFQVKVLNVTQS-YYDKYEMEITQVIKLGLESGV : 1689
Stickleback.C3.1.1: EREAGMLTRLCKKDQDECT-CAEENCSMQK---KDQVSNDQRTEKICETEVNNKIDFAYKVKVELSEEDSSIDVNTVRVLKTIKEGSKDVG : 1574
Stickleback.C3.1.2: EREAGMLTRLCTNPQDACT-CAEENCSMQK---KDQVSNDQRTEKICETE-ENKIDFVYKVKVELSEEDPSIDVNTVRVLKTIKEGSTDVG : 1565
Stickleback.C3.1.3: EREAGMLTRLCKKDQDECT-CAEENCSMQK---KDQVSNDQRTEKICETEVNNQIDFAYKVKVELSEEDPSIDVNTVRVLKTIKEGSKDVR : 1577
Stickleback.C3.2.1: REDREELSQICRN--DACR-CAQGDCCVSR-SDSENVPHQERETFACKT-----LHHVFKVKVLNVSQS-YYDKYEMEITQVIKLGES-IV : 1556
Stickleback.C3.2.2: REDREELSQICRN--DACR-CAQGDCCVSR-SDSENVPHQERETFACKT-----LHHVFKVKVLNVSQS-YYDKYEMEITQVIKLGES-IV : 1563
whale shark.C3.1.2: DENNAMLSKICRG--DVCK-CVEGSCLSVK-LPEHRISYRDRHERSCEHG----IDYVYKVTFRKKEKRDNYINYEMQVLIVIKQGTDDVY : 1571
Lamprey.C3x1        : PGGYVELSPLCQN--DLCQ-CVEVSCPAKKPKFDTSITVLHRQEAACVAG----IDYAYVGIVDNRTEVGSFVYYTVNIQTVIKSGQDQAI : 1592
Lamprey.C3x2        : PGGYVQLSPICQN--DLCQ-CVEVSCPAKKPKFDTSITVLHRQEAACVAG----IDYAYVGIVDNRTEVGSFVYYTVNIQTVIKSGQDQAI : 1592
```

C345C

```
Human.C3.1          : QVGQQRTFISPIKCREALKLEEKKHYLMWGLSSDFWGE-----KPNLSYIIGKDTWVEHWPEEDECQDEE-NQKQCQDLGAFTESMVVFGC : 1661
Chicken.C3.1.1      : PGGSNRTFVSHKQCRDALSLQKGQDYLVWGLASDLWVT-----GSRFSYLISKDTWLEAWPLEESCQDAD-LQPLCQDFTEFSDNMVLFGC : 1792
Coelacanth.C3.1.1   : PKDQRRNFISHKICRESLNLQLDKEYLVMGHSTDLWDT-----ATETNYIIGGNTWIEWWPTEAECQNPS-HQNLCNDFFQFADQMLVLGC : 1284
Coelacanth.C3.1.2   : PEKETREFISHKACKDALSLQLNKEYLVMGYSADLWDN-----TAKMKYLITERTWIEWWPTEVECQDSS-LRELCNNFFAFKEDMLIRGC : 1655
Spotted gar.C3.1.1: VQGKRRQFVAPSNCRRSSSFEKSKTYLIMGKINDLMKTSNGNMGKKWLYLLGGGTWLEWWPSDDECQKAQ-FQSTCENIKEVTLEIKAFGC : 1656
Spotted gar.C3.2    : SLPVQRMFLSHVACRDTLGLREGQKYFIIGR-----------------------------PQDQLCPKTD-TDP----------------- : 1604
Sea bass.C3.1.1     : PRGKQRTFLSPMHCRQALDLREGKTYLIMGTSKDIKHDDQ---DQLYLYVLGERTWIEYWPSEAECQTEE-YGNTCRGLEALEQGYGLFGC : 1369
Sea bass.C3.1.2     : PVGKLRIFLSYPHCREALDLRRGKTYLIMGTSRDIFIDEQ---SQTYQYVLGERTWIEYWPTEAECQIQK-HTAICSGIKDMAQQYKLKGC : 1651
Sea bass.C3.1.3     : PQGKLRTFLSYPHCRQALDLRTGKTYLVMGTSKDIYKDEENQSVTQYQYVLGERTWIEYWPSEAECQTEE-HGSTCRGMEELEQQYELFGC : 1661
Sea bass.C3.2       : EVGQKRLFMSHGGCRDGLSLKLGSQYLIIGP------------------------------------------------------------ : 1720
Stickleback.C3.1.1: PVNKLRTFISLPHCRESLDLRLGKTYLIMGSSQDITADEN---DQSFQYFLGGRTWIEYWPTDAECQKDE-YRPTCTGIENMVDQYETFGC : 1661
Stickleback.C3.1.2: PVNKLRTFISLAHCRESLDLRLGKTYLIMGSSRDITADEN---DQAFQYFLRERTWIEYWPTDAECQKDE-YRPTCTGFENMVDQYQTFGC : 1652
Stickleback.C3.1.3: PVNKLRTFISLPHCRESLDLRLGKTYLIMGSSRDITAKEN---DQSFQYFLGGRTWIEYWPTNAECQKDE-YRPTCAGIENMVDQYETFGC : 1664
Stickleback.C3.2.1: EVGQRRLFMSHGGCRDGLVLNQGSQYLIMGPTEDQWNADAD--TGRSVYVLGKDTWVERWPSPTECSSTDGLSDKCRSLKDAATELSVNGC : 1645
Stickleback.C3.2.2: EVGQRRLFMSHGGCRDGLVLNQGSQYLIMGPTEDQWNADAD--TGRSVYVLGKDTWVERWPSPTECSSTDGLSDKCRSLKDAATELSVNGC : 1652
whale shark.C3.1.2: PQN-IRELIIHANCEEIFNVELENDYLLMGQVIDLWKG-----DERISYVIGDGSWIEWWPNDVQCQNRQ-FARLCQNLEEFREILMVNGC : 1655
Lamprey.C3x1        : QPTAIRLFIVTRSCDGRLGMETPRQYLLMGRKGETKDK-----NDKFQYVLDSTSWVEQWPVDEKCNQPN-VQAFCAIKREYEFSMQIQGC : 1677
Lamprey.C3x2        : QPKATRLFIVTRSCDGRLGIETTRQYLLMGRKGETKDK-----NDNFQYVLDSSSWVEQWPVDEKCNQPN-MHAFCAIKREYQFSMQIQGC : 1677
```

```
Human.C3.1          : PN : 1663
Chicken.C3.1.1      : PT : 1794
Coelacanth.C3.1.1   : PN : 1286
Coelacanth.C3.1.2   : LN : 1657
Spotted gar.C3.1.1: PQ : 1658
Spotted gar.C3.2    : -- :    -
Sea bass.C3.1.1     : QQ : 1371
Sea bass.C3.1.2     : QQ : 1653
Sea bass.C3.1.3     : QQ : 1663
Sea bass.C3.2       : -- :    -
Stickleback.C3.1.1: QQ : 1663
Stickleback.C3.1.2: QQ : 1654
Stickleback.C3.1.3: QQ : 1666
Stickleback.C3.2.1: RL : 1647
Stickleback.C3.2.2: RL : 1654
whale shark.C3.1.2: DY : 1657
Lamprey.C3x1        : SS : 1679
Lamprey.C3x2        : S- : 1678
```
