# Supplementary material for: Specific Evolution and Gene Family Expansion of Complement 3 and Regulatory Factor H in Fish
Source: Front Immunol. 2020 Dec 14;11:568631. doi: 10.3389/fimmu.2020.568631 (PMC7768046; doi:10.3389/fimmu.2020.568631)
Supplement: Supplementary Figure 1 — Phylogenetic trees of the fish C1, C2, C6-C9 genes. Accession numbers of the sequences are available in Supplementary Table 1 . The trees were built with the BI method and posterior probability values are shown. [file DataSheet_2.zip › Supplementary Figure 6.PDF]

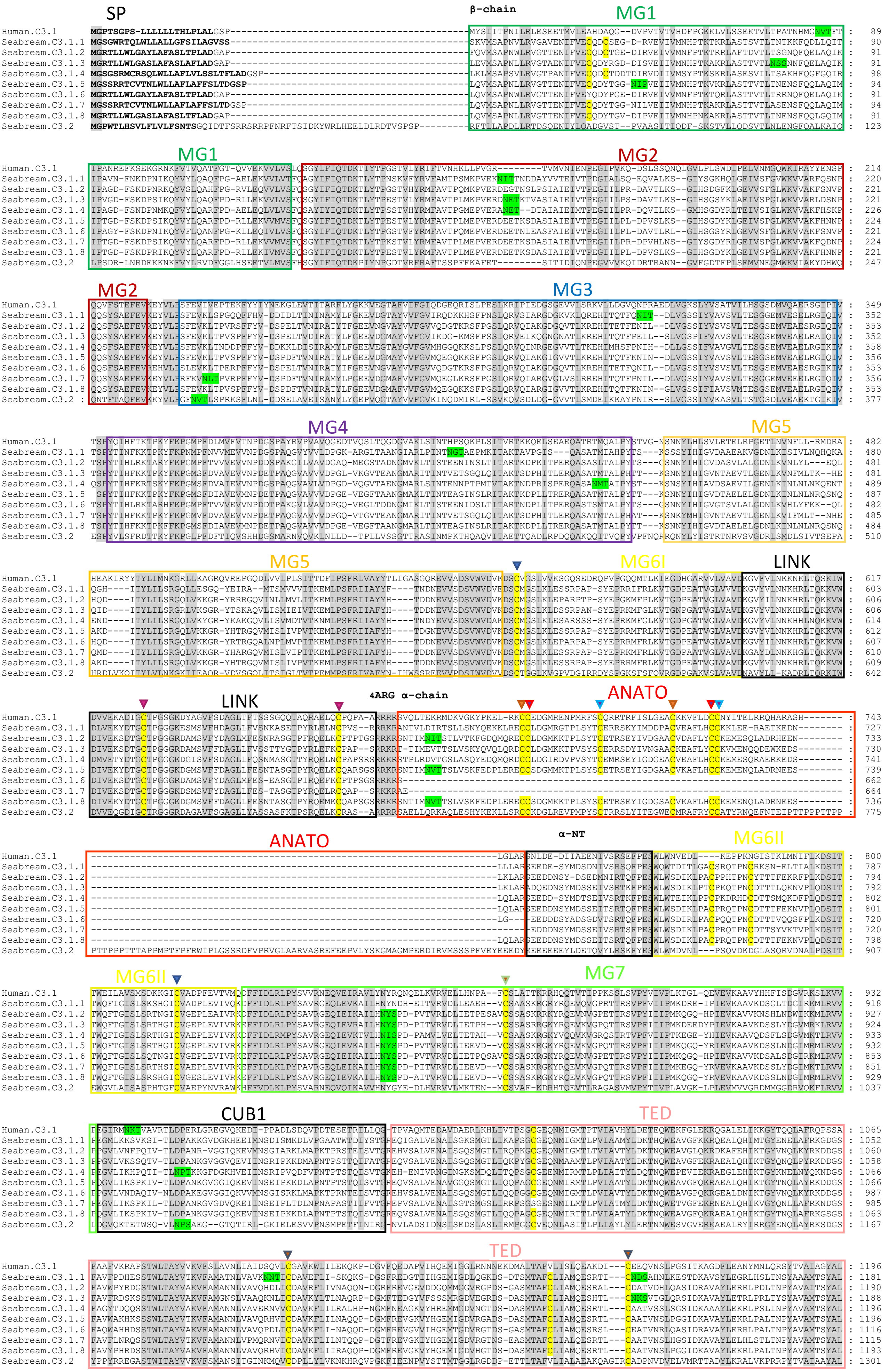
SP
β-chain
MG1
MG2
MG3
MG4
MG5
MG6I
LINK
4ARG α-chain
ANATO
α-NT
MG6II
MG7
CUB1
TED
Human.C3.1
Seabream.C3.1.1
Seabream.C3.1.2
Seabream.C3.1.3
Seabream.C3.1.4
Seabream.C3.1.5
Seabream.C3.1.6
Seabream.C3.1.7
Seabream.C3.1.8
Seabream.C3.2

```
                                                                        TED                                                          CUB2
Human.C3.1        AQMGRLKG--PLLNKFLTTAKDKNRWEDPGKQLYNVEATSYALLALLQLKDFDFVPPVVRWLNEQRYYGGGYGSTQATFMVFQALAQYQKDAPDHQELNLDVSLQLPSRSSKITHRIHWESASLLRSEETKE-NE : 1328
Seabream.C3.1.1   ANENKLNK--QILFKFRPT--GSTHWPVRDTQLFTLEATAYALLALVRAKDFENARPVVRWFNQQQIDGGGYGSTQATIMVYQAIAEYWIGANE-EEYEVNVDIKIPGRSKPEKFNFNRENHYTTRTAKLNKINQ : 1311
Seabream.C3.1.2   ANENKLNL--EILKNFVSS--ESSHWPVPRGHIYTLEATAYALLALVKVKALEDAKPVVRWFIKQQKVGGGYGSTQATIMVYQAVAEYWAYAKE-EEYNLNVDISLPGRSKPEKYNFNRDNHYTTRTSRINNINE : 1320
Seabream.C3.1.3   ANANKLDR--TILYKFASP--DSSHWPVPYSNLYTLEATAYALLALVKVKAFEDAKPVVRWFNEQLKVGGGYGSTQATIMVYQAVAEYWANAEE-EQYDLKLDILLPTKSDPEKYIFNRGNHYATRTSKINDINQ : 1318
Seabream.C3.1.4   ANENKLNQ--EILFKFASR--DLSHWQVPKGRVYTLEATAYALLALVKAQAFDDARPVVRWFNKQLRENGGYGSTQATIMVYQAVAEFWTSPKG-EEYDLNVDILLPGRSKPTKYNFNRDNLYATRTSKINNINQ : 1326
Seabream.C3.1.5   AQENKLNR--EILYNFISP--EMSHWPVPKGRVYTLEATAYGLLALVKAKAFEDARPVVRWFNKQQKVGGGYGSTQATIMVYQAVAEYWASAKE-EEYNLDVDILLPGRSKPDKFNFNRNNHYATRTSKTNDINE : 1326
Seabream.C3.1.6   ANENKLNL--EILKNFVSP--ESSHWPVPKGHIYTLEATAYALLALVKVKALEDAKPVVRWLIKQQKVGGGYGSTQATIMVYQAVAEYWAYAEE-IEYNLNVDILLPGKPKAEKYNFNRDNHYTTRTSRIDSINE : 1246
Seabream.C3.1.7   ANENKLNR--EILYNFVSP--ELSHWPVSKGRVYTLEATAYALLALVKAKAFEDARPVVRWFNQQQRVGGGYGSTQATIMVYQAVAEYWATAEE-QEYNLDVDILLPETSKADKYNFNRKNFHATRTSKFTAINK : 1245
Seabream.C3.1.8   AQENKLNR--EILYNFIST--ERSHWLVPKGRVYTLEATAYGLLALVKAKAFEDARPVVRWFNKQQKVGGGYGSTQATIMVYQAVAEYWASAQE-EEYNLDVDILLPGRSKPDKYNFNKNNHYATRTSKTNDINA : 1323
Seabream.C3.2     ALMGNPQQYNPTDYLLRASVAGRSHWPDSHNTLFTLEATGYALLALVKLGQMEAAAAPFKWLNGQRRRGGGFGSTQSTMVVLQALSEYLIHNPS-NQLSLDVDVRMTGR-KEIRYHFNPQTAYVARTSRLPA-NL : 1433

                        CUB2                                                                                MG8
Human.C3.1        GFTVTAEGKGQGTLSVVTMYHAKAK-DQLT-CNKFDLKVTIKPAPETEKRPQDAKNTMILEICTRYRG-DQDATMSILDISMMTGFAPDTDDLKQLANGVDRYISKYELDKAFSDRNTLIIYLDKVSHSEDDCLA : 1460
Seabream.C3.1.1   DLTVSATGSGEATVKMVSLYYALPE-ETESDCQKFNLSVQLKP-----ENMNTDEKTYKLIITILYKDEERDATMTIVDIGMLTGFIADQTDLKALSDGHARTISKFEQNTVLSARGSLIIYLNKVSHTEPEEIA : 1440
Seabream.C3.1.2   NVTVTATGTGEATVTMVSLYYTLPT-EKESDCQKFNVSVQLIQ-----DRNDDDMKSYKLRIDVLYKD-MRDATMSILDIGLLTGFTANTDDLNLLSKGHARTIANYEMNTVLSERGSLIIYLDKVSHKRPEEIT : 1448
Seabream.C3.1.3   NVKVTANGTGEATVTVVSLYYALPK-ENENNCQKFDMSVQLIS-----DGMDENGEIYKLKIEVLFKDKVHDASMSILDIGLLTGFNVDTNDLKLLSEGHARIIAKYEINKALSKKGSLIIYLDKVSNTQKEEIT : 1447
Seabream.C3.1.4   RVKVSAMGKGEATVTMVSLYYALPK-EKDSDCQKFNLSVELIP-----EKMDEDEKIYRLKILVLFKDKKRDAAMSLLDVGLLTGFTANTNDLSLLSKGRARTISKYTMVTADSERGSVIIYLDKVSHTRPEEIT : 1455
Seabream.C3.1.5   NVTVTATGSGEATVTMVSLYYALPS-EKESDCQKFNMSVQLIP-----DKNDGVETSYKLRIEVLYKDKERDATMTILDIGLLTGFTVDRNDLDLLSKGRARIISKYEMNTLLSEKGSLIIYLDKVSHTRPEEIT : 1455
Seabream.C3.1.6   NVTVTATGTGEATVTMVSLYYTLPT-EKESDCQKFNVSVQLIP-----DRNDDDMKSYKLRIDVLYKDEERDATMSILDIGLLTGFTANTEDLNLLSKGRARTIANYEMNTVLSERGSLIIYLDKVSHTRPEEIT : 1375
Seabream.C3.1.7   DVKVTAMGNGEATVTMVSLYYALPT-QIESYCQKFNLSVQLLP-----DKITADETIYRLRIEVLYKDNERDATLSVLDIGLLTGFTIDTNDLDLLSKGRARIIARYEMNTVLSERGSLIIYMDKVSHTQPAEIT : 1374
Seabream.C3.1.8   NVTVTATGSGEATVTVVSLYYALPS-EKENDCQKFNMSVQLIP-----DKNDGVETSYKLRIEVLYKDKERDATMTILDIGLLTGFTVDRNDLDLLSKGRARIISKYEMNTLLSEKGSLIIYLDKVSHTRPEEIT : 1452
Seabream.C3.2     DLEVVARGNGQGILEVVTYYNQLHEVDEKIPCNHFELSVTIEE--SSEKPPADVEKSYQITIKVRSLG-PRDVRMVVLDISLPTGFTPENSDLEMLSNSVDRYINNFQIVDNLSDRGSLIIHLFKVSHKVPEILI : 1565

                         MG8                 SAR                                   C345C
Human.C3.1        FKVHQYFNVELIQPGAVKVYAYYN----LEESCTRFYHPEKEDGKLNKLCRDELCRCAEENCFIQKSD-DKVTLEERLDKACE----PGVDYVYKTRLVKVQLSNDFDEYIMAIEQTIKSGS------------ : 1573
Seabream.C3.1.1   FKIHQKMAVGVLQPAAVSIYEYYDHQHNNQTPCVKFYHPERREGKLLKLCTNE-CTCAEENCSMQKKE--KISNDERTPKICETQQNSKIDFAYKVKLEDFTEKLSTDIYKMRIVDIIKEGSDECQPGKNGSRTI : 1572
Seabream.C3.1.2   FRIHQKFDVGVLQPAAVSVYEYYD-----QTPCVKFYRPERVAGELLRLCRGDECTCAEEDCTKQKKG--KISNEERTAKVCESTETSKTDFVYKVRLEKLKDSQSTDIYTMQVLDVIKEGSER----------- : 1565
Seabream.C3.1.3   FKIHQKFKVGVLQPAAVSVYEYYD-----QTPCVKFYHPERRAGQLMRLCRGDECTCAEEDCSMQKKG--KISNEDRKAMACESTLTSITEFVYKVRLEEFTDGLSTDIYIMQVLDVIKEGSF------------ : 1563
Seabream.C3.1.4   FRIHQKLNVGVLQPAAVSVYEHNDGRHSNQTHCMKFYHPERRAGQLLRLCRGDVCICAEENCSMQKKG--NISNADRTAKVCETELNSRIDFAYKVRLEEFTDGLSTDIYTVRVLEVIKEGN------------ : 1575
Seabream.C3.1.5   FRIHQTFKVGVLQPAAVSVYEYYE-----QTPCVKFYHPERRAGHLLRLCRGDECTCAEENCSMQKKG--KVSNEQRTAMACESTAISKIDFVYKVRLEEFTDSQSTDIYTMRVLEVIKEGSY----------- : 1571
Seabream.C3.1.6   FRVHQQFNVGVLQPAAVSVYEYYD-----QTPCVKFYRPERVAGKLLRLCRGDECTCAEENCTKLKKD---RSNADRNAKVYERTETSKTDFVYKVRMEELTVSQSTDIYTMQVLEVIKKGGY----------- : 1490
Seabream.C3.1.7   FRIHQRFNVGVLQPAAVSVYEYYE-----QTPCVKFYHPERRNGQILRLCRNDECICAEEDCGMQRKG--PISSVERRAKASEITPASRIDSVFKVRVENFTDGMSTDIYTARIVQVIKEGS------------ : 1489
Seabream.C3.1.8   FRIHQTFKVGVLQPAAVSVYEYYE-----QTPCVKFYHPERRAGHLLRLCRGDECTCAEENCSMQKKG--KVSNEQRTAMACESTAISKIDFVYKVRLEEFTDSQSTDIYSMQVLEVIKEGSY----------- : 1568
Seabream.C3.2     FRLQQNFKVGLLQPSSVTVYEYYN----PDHRCSRTYTPSEDKEELTKICRDNVCRCAHGDCCVSKTDSENFPNKDRETFAC-----ATLHHVFQVKVLNVSQSY-YDKYEMEITQVIKLGV------------ : 1677

                                                  C345C
Human.C3.1        -DEVQV--GQQRTFISPIKCREALKLEEKKHYLMWGLSSDFWG-EKPN--LSYIIGKDTWVEHWPEEDECQDE-ENQKQCQDLGAFTESMVVFGCPN----- : 1663
Seabream.C3.1.1   CDVGPL--DKLREFLSFQHCRDGLNLKIGKTYLIMGVAQDIRK-DEENQSFQYVLGERTWIEYWPTAAECQTA-EYRPICLGLEDMVEAYTIFQCLAK---- : 1666
Seabream.C3.1.2   -VVLQDGQNQTRSFLSYQHCRASLDLKEGKTYLLMGTSDDIYR-DHQDDSFQYVLGEKTWIEYWPTEPECETD-IYKPTCSGMEE-VKQHALTGCKL----- : 1658
Seabream.C3.1.3   -DVVDV--NNNITFLSYQHCRESLDLKKGKTYLVMGTSRDMYR-DEQNAVFQYILGERTWIEYWPTNAECQNV-NYRPTCLGMEDLEEVVKIFGCQAK---- : 1656
Seabream.C3.1.4   -DVGPL--GKLRTFLSNPHCREALDLRKGKTYLIMGDS-ERRT-DEQSQAYEYVLGERTWVEYWPTEAECKSQ-KHKLTCSGMLEMVQKYTQHGCEQ----- : 1666
Seabream.C3.1.5   -DVGPQ--NQVRAFLSYQHCRESLDLTEGKTYLIMGTSKDIHR-DEQNQSFQYVLGERTWIEYWPTEAECQTD-IHRPTCLGMEELVQQYALFGCEH----- : 1663
Seabream.C3.1.6   ------------------SCLNCL--------------------------------------YYPL--------VFSLKCV------------------------ : 1507
Seabream.C3.1.7   ---GSRFLGKPVTFISHPHCRAALDLRPGKTYLIMGGSKDIHR-DEQSRSYQYVPSKRTWIEYWPTDAECQTA-AYRPTCLGIDDLVNLYALFRCL------ : 1580
Seabream.C3.1.8   -DVGPQ--NQVRAFLSYQHCRESLDLTEGKTYLIMGTSKDIHR-DEQNQSFQYVLGERTWIEYWPTEAECQTE-IHRPTCLGMEELVQQYALFGCEH----- : 1660
Seabream.C3.2     -EAGVE-AGQTRFFMSHGNCRDGLKLEQGSQYLIIGPKEDQWNIDSDTNKYIYMLGKDTWVERWPSSAECSSNSTLEAKCKSLDDAAYELSVNACSL----- : 1772
```
