# Supplementary figures and images for: Specific Evolution and Gene Family Expansion of Complement 3 and Regulatory Factor H in Fish
Source: Front Immunol. 2020 Dec 14;11:568631. doi: 10.3389/fimmu.2020.568631 (PMC7768046; doi:10.3389/fimmu.2020.568631)

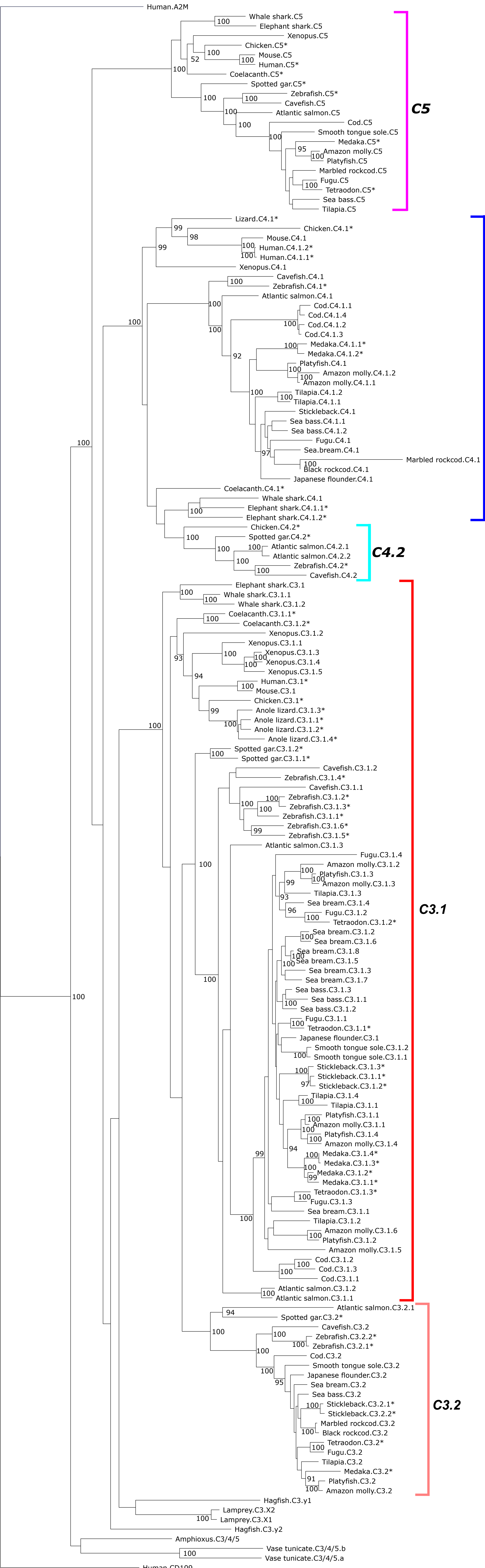

C5

C4.1

C4.2

C3.1

C3.2

Supplement: Supplementary Figure 1 — Phylogenetic trees of the fish C1, C2, C6-C9 genes. Accession numbers of the sequences are available in Supplementary Table 1 . The trees were built with the BI method and posterior probability values are shown. [file DataSheet_2.zip › Supplementary Figure 2.PDF]
