# Supplementary material for: Specific Evolution and Gene Family Expansion of Complement 3 and Regulatory Factor H in Fish
Source: Front Immunol. 2020 Dec 14;11:568631. doi: 10.3389/fimmu.2020.568631 (PMC7768046; doi:10.3389/fimmu.2020.568631)
Supplement: Supplementary Data — Nototheniid complement and Cfh deduced protein sequences used in the construction of the phylogenetic trees. [file DataSheet_1.docx]

**Supplementary Data**

>Marbled rockcod.C3.2

GFQSVTMARWTLANFLFLLLLCSSESSDRNVLRRPSWINRFTPIEHFWELHERPRLRVTVSSEPRFTVLAPDLLRADSQENIFLQAEGLSSPVTVSISILDYSKSKTFLQDSVTLNLENGYHTLKTLQLSSNGLKREEKSHKYVYLKVHFVGYHTIEKILMVSFHSGYIFIQTDKPIYKPGDTVRFRVFTSSLDFKAFNSSVTIDIQNPDGVVVKQVSRIQAFDGVFADTFPLSEIVNEGQWTVIAKFDHREQNTFTSQFQVKKYVLPAFNVTLTPKKSFLDLDDTELEVEIWARYLYGEPVQGTAYVVFGVKINQEMRRLPGVMQVTDLDGGVVKLSIEELKRAHPNIRSLVGSSVYVKASVLTKSGSDLVEAEKTGIKIVESPYAISFKDVPKYFKPGLPLDFTIQVSQHDGSPARNVIVKVTFLDTPLVVSSATTRATVNMPPQQHAQTITAETVQAGLRPEQQAKQQITVQPFAALRSRKPNYLYISTGTNTVSVGDSLSLKMSITVADPTHRQYITQITYLVVNKGKIIVAERLDVAGQLITSVGLHITPEMMPSFRFVAFYSIPWDEGHEVVPDSIWVDVEDSCAGGLNVGPVDGIGRDYTPGKSFSFQIRGDPGAKVNLVAVDNAVFLLNRDRLTQGKIWGTVEHGDIGCTRGGGQNALAVLSDAGLLFASSTGLETLIRQALKCPGSARRRRSAEKLQRKAELESHYKEKLQRRCCKDGLREIPMPYSCTRRSLYITEGWECMKAFRYCCATYRNQEFNTEVPTTPQPFTTPPPTTTPQPMPTFPIFFLRQMVPNAEEIRPFSREIGSRQFPQQRPRPDADSLLWFTARKNFIEKEEEVEADEEEEWEYLDETEVSLRSKFYESWLWTDVDLPTKADRDGLASANVDHPLPDSITEWGVLASSSPHTGFCVAKPYNVKAWKPFFVDLRLPYSVARNEQVEIKAVVHNYGDGELHVRVVLMKTEGMCSIAFKDRHTQEVTLAAGASVALPYTIVPLVVGKLPLEVMVVARDAMGGDRIQKLLNVVMDGVQKTEVWSAVLNPSAEGGTQTVSVPTVNLDSVVPNSAPETFINVRGNVLADSIDNSISEDSLASLIRMPGGCVEQNLASITLPLIATLYLETTNSWESVGVQRKAEALRYIRRGYENQLAYRKSDGSYPPYTREGASTWITAYVVKVFSMAHSIVGINEQQVCEPLLYLVNNKHKMSWGNFVEDNPVYSTTMTGGLRGDDPETTLTAFVLIALAEAKHAGISCVSSRVKVVIRKTAEYLKRALVTPGRRPYTVAIASYALALLGKDQNYNPTQSLLRAAAPGGSHWLDSKNTLFSLEGTGYALLALVKLGRMEEAAAPFKWLNSQRRRGGGFGSTQSTMVVLQALSEYLIHKPPPADLILDVDVKMRGRREIRYHFNSENSYAARSSRLPAGLDLEVEARGNGQGILEVVTYYNQLHEVDEKMPCKDFELKVNIEESSEKPPADVEKSYQITIKVRALGPRDVRMVVLDISLPTGFTPENSDLEMLSNSVDHYINDFKIVDNLSDRGSLILHLFKVSHKEPEILIFRLQQRFKVGLLQPSSVTVYEYYNPDHRCTHTYSPREDREELTQICRDNICRCTQGDCCVPKSESESFLNKERETFACTTLHHVFQVKVLSVTQSYYDKYEMEITKVIKLGVESGVEVGQKRVFMSHGGCRDDLNLQPGSQYLIMGPKVDQWNIDTDTNRFIYMLGKDTWVERWPTPAECSSRPELQAKCKSLHDTAEELSVNACRL

>Marbled rockcod.C4.1

HPYAVAITAYCLAVCMPQGTDHSSALRRLQTLATEVENGCNQRTANDSPQNQKADAITVETAAYTLLAAVELKQTKMADKTACWLTTQENYFGGFKSSQDTIMALEALAEYELKRNTSPEANLIAEFTVPGKRDIAKLTLKNKKERVETDLKKLAGNNIIVKLTGNGDTKLKIVKAYYLLDSKDHCDKVSISVRVEGKVKYTAEIVENYDYYEDYSVNKEKEVRVARSAIEWFDARTRNRRDLDNNLQSENTVTYNVCVSHSLDRNLTGMAIADITLLSGFQVETQDLDRVSTTLYLLFVINILRAVGGASFHFHSCILSFNTRQAKGFEK

>Marbled rockcod.C5

FAVRMKVCVLLMCVCGFFWRTEAESRSYLITAPLALRLDAVETVLLQLFGFTEEVTLHVFLKTSMASVNGVLVREVVTLNAQNQHQAQARVKLHPGQLDKSVSHVILHVQSAEINQHLSIPVIRTNGFLFVQTDKPLYNPHQAVKVRAFSLNQELRPANRSVFLTFKDPDRTTVDIVEMIDVNNGIPSMQNPFKIPIKPKLGIWSIEAAYSDDFTTTARADFEVKEYVLPSFYILVEPETNYVSSGNFKSFNFKVSARYVHGAPVAEGEVFLRYGYVSGTNPPVIIPSSVSRERLSSAGDLDVTVNMEKVLSKHDGPRDLDSLVGKYLYIAVLLREDTGGITQEAEFAAVKFLKSPYRLSLVSTPPFIKPGLPYNIQVVVKDHLDQPVNRVKVRLVERQLFKKGGASEDLPCPLSSVSQSDGIAVFICNTPREGVRALLKFQTEVPSLPAASQALLVLEALAFHSPNQRYLYIDPPMPGSSLEAGHFGNIKVYSTSPSYVPIRALSYLVLSKGKVVDFGSQKFVSSHDHRQVLNFEVTPAMVPSIRLLVYYILFGEGTSELVADSVWLDVKGKCVNDLQTDLSYSSGVYKPKQNLHLNIRTNQDGLVALSAVDSALFALKPNYRDPVSMVMSHLERSDLGCGGGGGRDSADVFRLAGLTFITNANAQPSAISAACTAAVRPRRALTEEDKVKKAESYGQVKSCCEHGMRYIPKSVTCHQFSVQRFRKHPRCRQVFRACCEFMQQDLDQDQDLVLGRHELGAEFDVAPSLVRSFFPESWMWEVQRVSSGKTPVTRPLPDSLTTWDIRAVGVFSNGVCAADTVQVSVALPLSVDIPLPYQLVRGEQLELSGSVYNQQLDNIQFCVTLTAGPGLCLQESQPGGWGLQSTACTWRHLSAGGVGKVEFTLLGLEPGEHTLTFTLKTKGGGRDVLEKKLRVVPEGVKKEVFSGGRLDPQGLYGSEKRVVQLRNKLPTNIVPNTAVERMITINGEVLGDILAVLHSPEGLRQLINLPAGSGEMEAGGLLLRAQVYLYLESSRRWEALGGDIEKSSADLRRSIADGLVSLSSFRREDSGYSMWVNREASTWLTALVVRTLSLVDSVVSLDHQKLSESVSWLIRDQQQQDGSFRDLSSNRPNRIMAAGTPALDRSVYLTSFVLIALHRATSIKDPILQLRFHDDSMSSAVNYITQHASGVKSVYVRAVATFALTLRDASSPIASELMISLEKLARDKGHPAELRYWQEASVAADWLKPDESSGLTVETTAYVLLTVLLKGRIPYANPILTWLTQDQHYGEGFYSVQDTVLTLESLTEYSRVVPRAVLDQDINVRYGRKGPLGRVQLSQSRPVATPIQVTKEDAITVSTGYGKGVSNVKLKTVYYETTPSAQNCNFHLTIEAVGPNNSTNAGMTAPHLVACAKYKPPPNELFTESSLTVMKIQLPTGVEAFLEDLRQFRDFQEPSISHFELQGNTVIVLADSVPSEDFLCIGFRIRMGFRVEGASESLFSVYEPQDKGSECTRQFSYQQQKLQRLCVDEKCQCMTAACAAYRGNMDPTLTVNKRTTETCRPHITYAYRVTVKSSAAEGDFMSYTATVEEILKNTDTQLEAVSSGSDVELVKKVTCSSVDLQNNKQYLVMGAGGSEVTLSQGFKYRLPMDSEALVDLWPTVCSSPECTDYISHL

EDFALDLQLTGCPNSS

>Black rockcod.C3.2

MNTSSDLVEAEKTGIKIVESPYAISFKDVPKYFKPGLPLDFTIQVSQHDGSPARNVIVKVTFLDTPLVVS

SATTRATVNMPPQQRAQTITAETVQAGLRPEQQAKQQITVQPFAALSSRKPNYLYISTGTNTVSVGDSVS

LKMSITVADPTHRQYITHITYLVVNKGKIIVAERLDVAGQLLTSVGLHITPEMMPSFRFVAFYSIPWDEG

HEVVPDSIWVDVEDSCAGGLNVGPVDGIARDYTPGKSFSFQIRGDPGAKVNLVAVDNAVFLLNRDRLTQG

KGGLRGDDPEITLTAFVSIALTEAKQARISCIGFIETVIFNKTAEYLKRALETPGRRPYTVAIASYALALLGKDQNYNPTQSLLRAAAPGGSHWPDTKNTLFTLEGTGYALLALVKLGRMAEAAAPFKWLNSQRRRGGGFGSTQSTMVVLQALSEYLIHKPPPADLILDVDVKMRGRREIRYHFNPENSYAARSSRLPAGLDLEVEARGNGQGILEVVTYYNQLHEVDEKMPCKDFELKVNIEESSEKPPADVEKSYQITIKVRALGPRDVRMVVLDVSLPTGFTPENSDLEMKEPEILIFRLQQRFKVGLLQPSSVTVYEYYNPDHRCTHTYSPREDREELTQICRDNICRCTQGDCCVPKSESENFLNEERETFACTTLHHVFQVKVLSVTQSYYDKYEMEITQVIKLGVESGVEVGQKRVFMSHGGCRDDLNLQPGSQYLIMGPKVDQWNIDTDTNRFIYMLGKDTWVERWPTPAECSSRPELQAKCKSLHDTAEELSVNACRL

>Black rock.C4.1

MERYIFSILFLILTVEPAASTDNGFFISAPGVFHVGVNEKVFVQMGKSHFNIPVTLYLELETGGLLSNKI

TTTCTEDNKIQTVELKLRTDLVADLPPEVRKTLKYLTLVAVSPAFSVRKSTKVLVSKRRGKIFIQTDQPI

YNPTRKVNYRIFTLDHAYRPSEDVIQISVINAAGNRVMRSQRSAKGGILKGNFPIPDVSKMGTWKITAHY

ENDEDNAASREFKVKKFVLPSFEVNIAMKQRYILLNAEEFNFTILARYSHGEKVKGAYHCQFGVVVKETT

LGERMKPVFIRRLELTGSVQDGTAAATLQMAELRNQLQIQQNKSISELQQSGAQIYLGVFVTNIQSGEIQ

ETEVYLPIISHKYTVDLSRTRKYFLPGYPLDVVAVVRHPDGSPAAGVPVKMEVKPEESWHGITDQEGAVF

HVFNIQNEDRITVKVSADGLQVKKVIQKASSPSNCFLYLSITHRMYSVGETLTVNFNTINAPTQGLIYYM

VFSRGILINQGSVILGTSVRQNLPITSDMVPSFRLIGYCYNQNGDIIADSVWVDVMDECQIKAKVETKGP

FTPGKRSVLEIDLDGQRAKVALLAVDKAFYGLNADNKLTAKQVFSSMQSFDHGCTYGGGADPASVLIDAG

LSFFSQSDSKWRKSLSCNSQAGRQGRSVNLQQEMMSLKSNFSKEEFQECCVQGFSLIPMRRTCLERVKRI

NLVEAKPGCAEAFFKCCLEGERLRKKKMIEETQDELGRTASTEDIEDFFLDTTAQYIRQFFPPSFAFTEF

EVNGKGSYNLALPDSITMWEIQVVTLSAATGFCVVKPSEVRAFKSVFVSLKMPYSVKKYEQLSISPVIYN

YGDDTLQLAVHMEQTEGLCSPGSATTTAFVNITVEPQSSQFVSFSAVPMVIGSIPIKIRLYDIGNEWGID

AIEKTLNVLTEGLEKRVEVTYVQKFVGRSSKNFTYDGTLPDDVVPNSLSNIFISAEGDGFGSSHAENLLS

PQKVSRLIVLPTGCLEQTMSKLAPTVSALRYLDLSRQWFDLPAGTRDDALDKIEEGYIRILGYKKPNGSY

GAWGTVPSSNWVTALVVKVLSLVAQRQAMAFGQQGRQARVVPEKEIRLSVGYLISVQNSDGSYRDPHPVL

HKGVLEDQEDKASMTAFINLALYRSLDFLNSELRNNVEASISRSKTYLRSQPEELKHPYAVAITAYCLAV

CMPQGTDHSSALRRLQTLATEVENGCNQRTANASPQNQRADAITVETAAYTLLAAVELKQTKMADKTACW

LTTQENYFGGFKSSQDTIMALEALAEYELKRNTSPEANLIAEFTVPGKRDIAKLTLKNKKERVETDLKKL

AGNNIIVKLTGNGDTKLKIVKAYYLLDSKDHCDKVSISVRVEGKVKYTAEIVENYDYYEDYSVNKEKEVR

VARSAIEWFDARTRNRRDLDNNLQSENTVTYHVCVSHSLDRNLTGMAIADITLLSGFQVETQDLDRLTLL

PEQYIAHYEATYGRVVLYFNKVLESKECISFDATQTVPIALLQPAPAVFYDYYEPDRRCTVFYSAPKRST

MISKLCSEDVCQCAERPCHKIQNTFQRRPRMTKYVRLQHACFFPVVDYAYSVEVLNVTMKSNFELYSVNV

TDVLRSHGDILVSENSVRVFAKRLHCKGQLDLGKQYLIMGKDGATTDSNGKMQYLLDSNTWVERKPLKDT

CKKSAYRSACTEFDTFTEDYKIDGCRQ
